# Arid3b suppresses CD8 + T cell infiltration and function in microsatellite-stable colorectal cancer via Runx3

**DOI:** 10.1038/s41467-026-73241-7

**Published:** 2026-05-15

**Authors:** Shuo Wang, Sen Hou, Ce Luo, Haorui Zhang, Yiteng Jin, Rui Zhang, Yanping Zhao, Xiaoyu Xiong, Rui Guo, Chao Wang, Yudi Bao, Liang Wen, Deng Pan, Yingjiang Ye, Zexian Zeng, Zhidong Gao

**Affiliations:** 1 Department of Gastroenterological Surgery, Peking University People’s Hospital, Beijing, China; 2 Laboratory of Surgical Oncology, Peking University People’s Hospital, Beijing, China; 3 Center for Quantitative Biology, Academy for Advanced Interdisciplinary Studies, Peking University, Beijing, China; 4 Peking-Tsinghua Center for Life Sciences, Academy for Advanced Interdisciplinary Studies, Peking University, Beijing, China; 5 Tsinghua-Peking Center for Life Sciences, Tsinghua University, Beijing, China; 6 Department of Obstetrics and Gynecology, Seventh Medical Center of Chinese PLA General Hospital, Beijing, China; 7 Tsinghua-Peking Center for Life Sciences, Department of Basic Medical Sciences, Tsinghua University, Beijing, China; 8 Peking University Chengdu Academy for Advanced Interdisciplinary Biotechnologies, Chengdu, China

**Keywords:** Immunoediting, Colorectal cancer, Cytotoxic T cells, Gene regulation in immune cells

## Abstract

Microsatellite-stable/proficient mismatch repair (MSS/pMMR) colorectal cancer (CRC) is characterized by a cold tumor microenvironment, with limited CD8⁺ T cell infiltration and poor responsiveness to immune checkpoint inhibitors (ICIs). Here, using an in vivo CRISPR/Cas9 screen in a CMT93 cell-derived murine tumor model, we identify *Arid3b* as a key negative regulator of CD8⁺ T cell infiltration and antitumor activity. Genetic ablation of *Arid3b* in CD8⁺ T cells significantly enhances their intratumoral accumulation and promotes robust tumor control. Mechanistically, *Arid3b* deficiency upregulates *Runx3*, driving a tissue-resident memory-like phenotype and effector function. Notably, the benefits conferred by *Arid3b* deficiency are abrogated upon *Runx3* deletion, indicating a RUNX3-dependent mechanism. Together, targeting ARID3B could offer a promising strategy to reshape the tumor microenvironment and sensitize MSS CRC to immunotherapy.

## Abstract (web summary)

Microsatellite-stable/proficient mismatch repair colorectal cancers are resistant to immune checkpoint blockade immunotherapy due to their immune suppressive tumour microenvironment. Here the authors show in mouse models that the transcription factor Arid3b contributes to the suppression of CD8 + T cell infiltration and activity in these tumours via suppressing Runx3, a known regulator of tissue resident memory phenotype in CD8 + T cells.

## Introduction

Colorectal cancer (CRC) accounts for ~1.9 million new cases and 930,000 deaths annually, ranking third in incidence and second in cancer-related mortality worldwide^1^. Although immune checkpoint inhibitors (ICIs) targeting the PD-1/PD-L1 axis have transformed the therapeutic landscape for the subset of CRC patients with microsatellite instability-high (MSI-H) or deficient mismatch repair (dMMR) tumors, these benefits are largely restricted to this minority group, which constitutes only about 15% of CRC cases^2–4^. In contrast, the majority of CRCs, characterized by microsatellite stability (MSS) and proficient mismatch repair (pMMR), exhibit limited responsiveness to ICIs, highlighting a critical therapeutic gap and underscoring the need to elucidate the mechanisms driving immune evasion in these cold tumors.

The response to ICIs in CRC is closely related to the immunological landscape of the tumor microenvironment (TME)^5,6^. MSI-H/dMMR tumors typically harbor a high tumor mutational burden (TMB), abundant neoantigens, and robust infiltration of tumor-infiltrating lymphocytes (TILs), features that collectively contribute to their favorable response to PD-1/PD-L1 blockade^7–9^. Conversely, MSS/pMMR tumors are generally devoid of these immunogenic features and are instead characterized by low TMB, poor CD8⁺ T cell infiltration, and a suppressive immune milieu^10,11^. Notably, CD8⁺ T cell density within tumors has been consistently associated with improved clinical outcomes in CRC^12,13^, underscoring the importance of identifying strategies to enhance CD8⁺ T cell infiltration in MSS tumors as a pathway toward overcoming ICI resistance.

*ARID3B*, a member of the AT-rich interaction domain (ARID) family, is a chromatin-associated protein that regulates gene transcription through binding to AT-rich DNA regions^14–16^. During embryonic development, ARID3B is essential for neural tube closure and placental vascularization, with knockout mice exhibiting embryonic lethality due to profound developmental abnormalities^17^. Beyond its developmental roles, ARID3B has emerged as a context-dependent modulator of tumorigenesis. It functions as an oncogene in several malignancies, including neuroblastoma, breast, ovarian, and colorectal cancers, where it promotes tumor growth, angiogenesis, and metastasis^18–21^. Intriguingly, in gastric cancer, ARID3B has been reported to exert tumor-suppressive functions^22^. Despite these insights into its roles in cancer biology, the function of ARID3B in T cell-mediated immunity remains unknown.

The advent of CRISPR/Cas9-based genetic screening has enabled systematic dissection of gene function across diverse biological contexts^23^. In particular, in vivo CRISPR screens have proven instrumental in uncovering regulators of immune cell behavior within the TME, offering insights into tumor–immune interactions and revealing novel therapeutic targets^24^. While recent studies have leveraged this platform to enhance antitumor immunity, its application in identifying intrinsic regulators of CD8⁺ T cells in immune-excluded tumors remains limited^25,26^.

In this study, we perform an in vivo CRISPR/Cas9 screen targeting genes in CD8⁺ T cells within a CMT93-derived MSS colorectal tumor model to identify regulators that limit T cell infiltration. We identify *Arid3b* as a key suppressor of CD8⁺ T cell tumor infiltration and antitumor function. Genetic ablation of *Arid3b* in CD8⁺ T cells leads to enhanced intratumoral accumulation and improved tumor control. Mechanistically, *Arid3b* deficiency results in upregulation of *Runx3*, a transcription factor essential for tissue residency and effector differentiation of CD8⁺ T cells. Our findings reveal the *Arid3b–Runx3* axis as a critical checkpoint regulating T cell exclusion in MSS CRC and offer a potential strategy to reprogram the cold tumor microenvironment to restore immunotherapeutic sensitivity.

## Results

### In vivo CRISPR screening identifies *Arid3b* as a negative regulator of CD8⁺ T cell infiltration

**Fig. 1:**
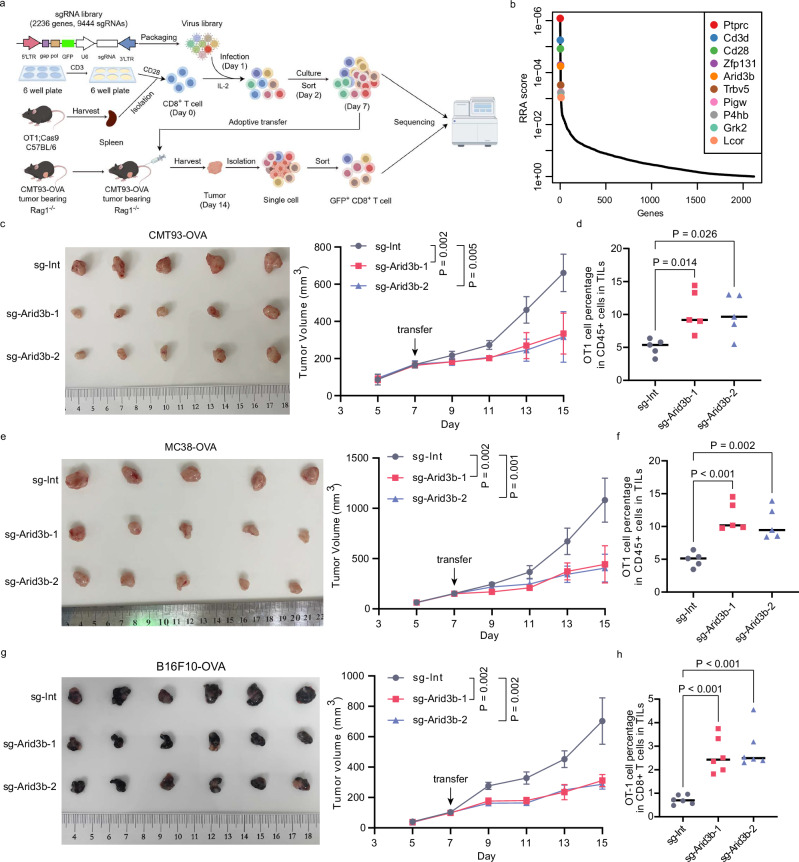
In vivo CRISPR screening identifies *Arid3b* as a key negative regulator of CD8⁺ T cell infiltration in MSS colorectal cancer. **a** Schematic overview of the in vivo CRISPR/Cas9 screening strategy targeting CD8⁺ T cells in a CMT93 tumor model to identify regulators of T cell infiltration. Created by Figdraw (ID: OURWUc8e8a). **b** Top candidate genes ranked by robust rank aggregation (RRA) score from MAGeCK analysis result, highlighting genes whose deletion in CD8⁺ T cell associated with enhanced CD8⁺ T cell tumor infiltration. **c**, **e** Representative tumor images and growth curves of CMT93-OVA (**c**) and MC38-OVA (**e**) tumors in *Rag1−/−* mice following adoptive transfer of OT-I; Cas9 CD8⁺ T cells transduced with control sgRNA (sg-Int) or *Arid3b*-targeting sgRNAs (sg-*Arid3b*-1/sg-*Arid3b*-2) (*n* = 5 mice). **d**, **f** Quantification of transferred OT-I; Cas9 CD8⁺ T cells within the tumor-infiltrating lymphocyte population (CD45⁺) from CMT93-OVA (**d**) and MC38-OVA (**f**) tumors (*n* = 5 mice). **g** Representative tumor image and growth curve of B16F10-OVA tumor in wild-type C57BL/6 mice following adoptive transfer of sg-Int or sg-*Arid3b* OT-I; Cas9 CD8⁺ T cells (*n* = 6 mice). **h** Frequency of adoptively transferred CD8⁺ OT-I; Cas9 T cells within tumor-infiltrating CD8⁺ T cells from B16F10–OVA-bearing mice (*n* = 6 mice). Data are shown as means ± SEM. P value was determined by one-tailed two-way ANOVA with correction using Geisser–Greenhouse method (**c**, **e**, **g**) or one-way ANOVA with Dunnett’s multiple comparisons (**d**, **f**, **h**). Source data are provided in Source Data file.

To systematically identify genes that restrict CD8⁺ T cell infiltration into the tumor microenvironment of immune-cold colorectal cancers, we conducted an in vivo CRISPR/Cas9 screening using a syngeneic mouse model. CMT93-OVA, a microsatellite-stable (MSS) murine colorectal cancer cell line, was subcutaneously implanted into female *Rag1−/−* mice to establish tumors. In parallel, CD8⁺ T cells were isolated from the spleens and lymph nodes of female OT-I; Cas9 mice, activated ex vivo with anti-CD3/CD28 antibodies, transduced with a pooled sgRNA library targeting immune-regulatory genes, and cultured with recombinant murine IL-2. A total of 2236 genes (9444 sgRNAs) representing membrane proteins, transcription factors, and enzymes were evaluated using a gRNA pool comprising 4 gRNAs per gene and 500 negative control gRNAs. Edited T cells were adoptively transferred into tumor-bearing mice via tail vein injection on day 7 post-tumor implantation (Fig. 1a).

Targeting key negative regulators enhanced T-cell infiltration, proliferation, and survival within tumors, leading to enrichment of the corresponding gRNAs. Seven days after adoptive transfer, tumors were harvested and processed into single-cell suspensions. Transferred CD8⁺ T cells were identified by GFP expression and sorted for downstream analysis. sgRNA abundance in these tumor-infiltrating CD8⁺ T cells was compared with pre-injection input cells using high-throughput sequencing (Fig. 1a). Interestingly, the Model-based Analysis of Genome-wide CRISPR/Cas9 Knockout (MAGeCK)^27^ revealed *Arid3b* as one of the top candidates enriched in intratumoral CD8⁺ T cells, indicating its potential role as a negative regulator of T cell infiltration (Fig. 1b).

To validate the functional role of *Arid3b*, we selected two sgRNAs with the highest enrichment scores to knock out the gene in CD8⁺ T cells. Western blot analysis confirmed efficient depletion of ARID3B protein (Supplementary Fig. 1a). These edited T cells were then transferred into female *Rag1−/−* mice bearing CMT93-OVA. Compared with control sgRNA (sg-Int)–treated cells, both sg-*Arid3b*−1 and sg-*Arid3b*−2 T cells significantly suppressed tumor growth in the CMT93 tumor (Fig. 1c and Supplementary Fig. 1b). This phenotype remains consistent when extended to the male *Rag1−/−* mice bearing colorectal MC38-OVA (Fig. 1e and Supplementary Fig. 1c). Flow cytometric analysis revealed a notable increase in the frequency of *Arid3b*-knockout CD8⁺ T cells among TILs (Fig. 1d, f). To assess whether these findings extend to an immunocompetent setting, we performed similar experiments using wild-type female C57BL/6 mice bearing B16F10-OVA tumors. Adoptive transfer of *Arid3b*-deficient CD8⁺ T cells again resulted in enhanced tumor control and a higher proportion of infiltrating CD8⁺ T cells compared to control T cells (Fig. 1g, h and Supplementary Fig. 1d). Together, these results identify *Arid3b* as a key suppressor of CD8⁺ T cell infiltration and demonstrate that its genetic ablation enhances T cell accumulation within tumors.

### Arid3b suppresses CD8⁺ T cell effector function, migration, and tumor infiltration

To further characterize the role of *Arid3b* in CD8⁺ T cell biology, we investigated its effects on T cell activation, exhaustion, effector function, cytotoxicity, and migratory capacity. We first assessed early activation and exhaustion status by examining the expression of the activation marker CD69 and the exhaustion marker TIM-3. Neither knockout nor overexpression of *Arid3b* altered CD69 expression in CD8⁺ T cells, indicating no significant effect on initial activation (Supplementary Fig. 2a, b). In contrast, *Arid3b* knockout led to a modest but consistent reduction in the proportion of TIM-3⁺ cells, while *Arid3b* overexpression significantly increased TIM-3 expression (Supplementary Fig. 2c, d), suggesting a role in promoting T cell exhaustion.

**Fig. 2:**
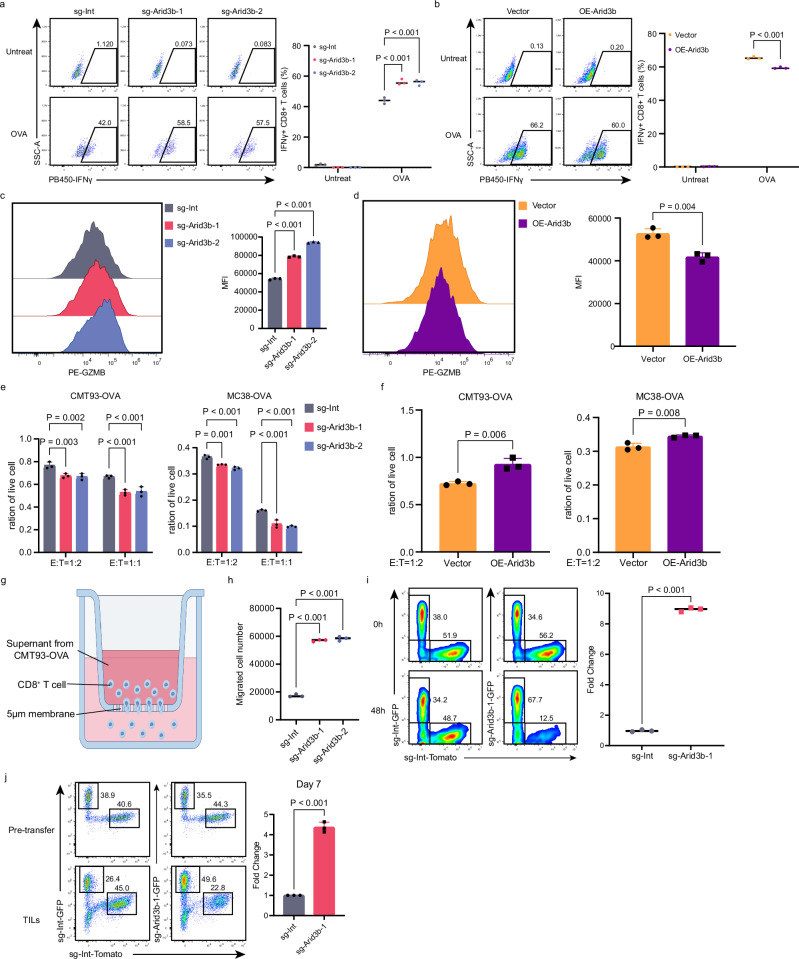
*Arid3b* suppresses mouse CD8⁺ T cell effector function and migration. **a**, **b** Flow cytometric analysis of IFN-γ production in CD8⁺ T cells following *Arid3b* knockout (**a**) or overexpression (**b**), with or without OVA peptide stimulation (*n* = 3 biologically independent samples). **c**, **d** Expression of granzyme B (GZMB) in CD8⁺ T cells following *Arid3b* knockout (**c**) or overexpression (**d**), assessed by flow cytometry (*n* = 3 biologically independent samples). **e**, **f** T cell-mediated cytotoxicity assay evaluating the killing capacity of CD8⁺ T cells against CMT93-OVA and MC38-OVA target cells following *Arid3b* knockout (**e**) or overexpression (**f**) (*n* = 3 biologically independent samples). E: T denotes the effector-to-target cell ratio. **g** Schematic representation of the CD8⁺ T cell transwell migration assay. Created by Figdraw (ID: IYRYUa12d3). **h** Quantification of migrated CD8⁺ T cells following *Arid3b* knockout, determined by flow cytometry (*n* = 3 biologically independent samples). **i** Competitive migration assay: Tomato⁺ sg-Int CD8⁺ T cells were mixed 1:1 with GFP⁺ sg-Int or sg-*Arid3b* CD8⁺ T cells and added to the upper chamber of a transwell system. The GFP⁺/Tomato⁺ ratio in the lower chamber was quantified by flow cytometry after 24 h (*n* = 3 biologically independent samples). **j** In vivo competition assay: Tomato⁺ sg-Int CD8⁺ T cells were mixed 1:1 with GFP⁺ sg-Int or sg-*Arid3b* CD8⁺ T cells and transferred into mice bearing subcutaneous CMT93-OVA tumors. Seven days post-transfer, the GFP⁺/Tomato⁺ ratio among tumor-infiltrating lymphocytes (TILs) was analyzed and normalized to the sg-Int control group (*n* = 3 mice). Data are shown as means ± SEM. *P* value was determined by two-way ANOVA with Sidak’s multiple comparisons (**a**, **b**, **e**), one-way ANOVA with Dunnett’s multiple comparisons (**c**, **h**) or unpaired two-tailed Student *t* test (**d**, **f**, **i**, **j**). Source data are provided in Source Data file.

Next, we evaluated the impact of *Arid3b* on effector cytokine production. *Arid3b* deletion in CD8⁺ T cells markedly increased IFN-γ and granzyme B (GZMB) expression following antigen stimulation, whereas *Arid3b* overexpression suppressed their production (Fig. 2a–d). These findings suggest that *Arid3b* restricts CD8⁺ T cell effector function. We then examined whether *Arid3b* modulates the cytotoxic capacity of CD8⁺ T cells. Arid3b-knockout or overexpressing CD8⁺ T cells were co-cultured with OVA-expressing tumor targets (CMT93-OVA, MC38-OVA, or B16F10-OVA). *Arid3b* deficiency significantly enhanced tumor cell killing across all lines, while its overexpression diminished cytotoxic activity (Fig. 2e, f and Supplementary Fig. 2e, f), indicating a cell-intrinsic role of *Arid3b* in cytotoxicity.

To determine whether *Arid3b* influences T cell migration, we performed a transwell assay using CMT93-OVA–conditioned medium as a chemoattractant (Fig. 2g). *Arid3b*-deficient CD8⁺ T cells exhibited significantly enhanced migration compared to control cells (Fig. 2h). To reduce batch variability, competitive migration assays were conducted. Specifically, control tdTomato⁺ sg-Int CD8⁺ T cells were mixed at a 1:1 ratio with either GFP⁺ sg-Int or GFP⁺ sg-*Arid3b*−1 CD8⁺ T cells and loaded into the upper chamber of the transwell. In the sg-Int–GFP/control group, the GFP:Tomato ratio in the lower chamber remained approximately 1, whereas in the sg-*Arid3b*−1–GFP/control group, the GFP:Tomato ratio was significantly elevated, indicating enhanced migratory capacity of *Arid3b*-deficient cells (Fig. 2i). To validate these findings in vivo, we adoptively transferred 1:1 mixed populations of GFP⁺ and tdTomato⁺ CD8⁺ T cells into CMT93-OVA tumor-bearing male mice. Flow cytometric analysis of tumor-infiltrating lymphocytes seven days post-transfer revealed that *Arid3b*-knockout CD8⁺ T cells preferentially accumulated in tumors compared to control cells (Fig. 2j).

Of note, we selected another top-enriched gene from the original screen, *Znf131(Zfp131)*, for preliminary functional validation. First, we confirmed the knockout efficiency of the ZNF131 protein in CD8⁺ T cells by Western blot analysis (Supplementary Fig. 2g). Upon antigen-specific stimulation, *Znf131*-knockout CD8⁺ T cells exhibited significantly elevated levels of IFN-γ and GZMB compared to controls (Supplementary Fig. 2h, i). Furthermore, in co-culture assays with OVA-expressing tumor cell lines (CMT93-OVA, or MC38-OVA), *Znf131* deficiency markedly enhanced the tumor-killing capacity of CD8⁺ T cells across all tested models (Supplementary Fig. 2j, k). These results reinforce the validity of our screening approach and indicate that *Znf131*, like *Arid3b*, functions as a negative regulator of CD8⁺ T cell effector function.

### Integrative analysis of RNA-seq, ATAC-seq, and ChIP-seq reveals Arid3b-dependent regulation of CD8⁺ T cell activation and effector programs

**Fig. 3:**
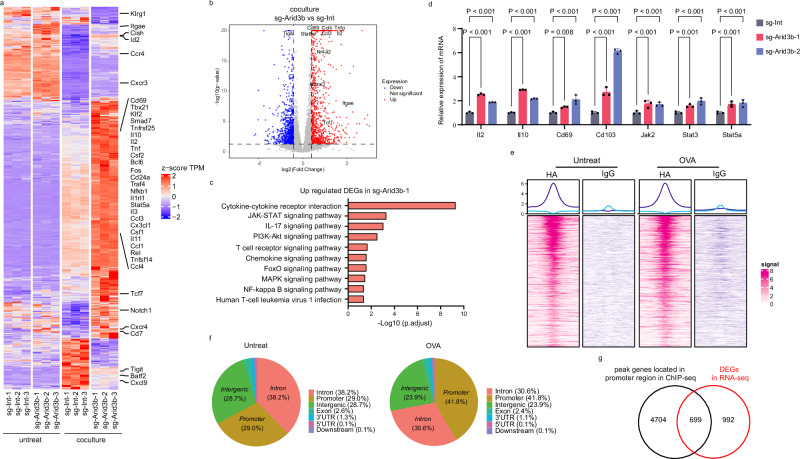
Transcriptomic and epigenomic profiling reveal activation and effector signatures in *Arid3b*-deficient CD8⁺ T cells. **a** Heatmap of differentially expressed genes (DEGs) in sg-*Arid3b* and sg-Int CD8⁺ T cells co-cultured with or without CMT93-OVA cells for 4 h (*n* = 3 biologically independent samples). **b** Volcano plot depicting DEGs between sg-*Arid3b* and sg-Int CD8⁺ T cells after 4-h co-culture with CMT93-OVA cells. The −log_10_ (adjusted *P* value) is plotted against the log_2_ (fold change). **c** KEGG pathway enrichment analysis of genes significantly upregulated in sg-*Arid3b* CD8⁺ T cells following CMT93-OVA co-culture. **d** RT-qPCR analysis of *Il2*, *Il10*, *Cd69*, *Cd103*, *Jak2*, *Stat3*, and *Stat5a* mRNA expression in sg-*Arid3b*-1/2 and sg-Int CD8⁺ T cells after 4-h co-culture with CMT93-OVA cells (*n* = 3 biologically independent samples). **e** Heatmap of ChIP-seq signal intensity showing genome-wide binding profiles in sg-*Arid3b* and sg-Int CD8⁺ T cells (*n* = 3 biologically independent samples). **f** Pie chart illustrating the genomic distribution of *Arid3b* ChIP-seq peaks, categorized by genomic features (e.g., promoter, intron, exon, intergenic regions). **g** Venn diagram showing the overlap between genes with Arid3b ChIP-seq peaks in promoter regions and DEGs identified by RNA-seq. Data are shown as means ± SEM. *P* value was determined by one-way ANOVA with Dunnett’s multiple comparisons (**d**). Source data are provided in Source Data file.

To gain a comprehensive view of the transcriptional program regulated by Arid3b and to identify potential downstream effectors that could explain the phenotypic changes, we performed RNA sequencing (RNA-seq), assay for transposase-accessible chromatin with high-throughput sequencing (ATAC-seq), and chromatin immunoprecipitation sequencing (ChIP-seq) analyses. CD8⁺ T cells transduced with sg-*Arid3b* or sg-Int sgRNAs were co-cultured with or without CMT93-OVA tumor cells for 4 h, and total RNA was extracted for transcriptomic profiling (*n* = 3 per group). RNA-seq identified substantial gene expression changes upon tumor antigen engagement. Using a threshold of *P* < 0.05 and absolute log_2_FC larger than 1, co-culture with CMT93-OVA induced 3954 differentially expressed genes (DEGs) in sg-Int cells (1940 upregulated, 2014 downregulated) and 4,729 DEGs in sg-*Arid3b* cells (2348 upregulated, 2381 downregulated). Of these, 3291 DEGs overlapped between the two groups, representing 83.2% and 69.6% of the respective DEG sets, suggesting shared core responses to tumor antigen exposure (Fig. 3a). Notably, direct comparison between sg-*Arid3b* and sg-Int groups revealed a more modest transcriptional shift in the absence of tumor co-culture (381 DEGs), but an amplified differential profile under co-culture conditions (1691 DEGs), highlighting the regulatory role of *Arid3b* during tumor-specific activation.

Key upregulated genes in *Arid3b*-deficient CD8⁺ T cells under tumor co-culture included *Cxcr3* (a chemokine receptor mediating T cell trafficking), *Cd69* (an early activation marker), *Il2* (a central regulator of T cell proliferation and effector differentiation), and memory-related genes such as *Tcf7*, *Bcl6*, and *Itgae* (*Cd103*). In addition, *Jak2*, *Stat3*, and *Stat5a* were elevated, suggesting enhanced signaling downstream of cytokine receptors. Conversely, downregulated genes included *Klrg1* (a marker of terminal differentiation) and *Tigit*, which is associated with T cell dysfunction (Fig. 3b). KEGG pathway enrichment of upregulated genes in sg-*Arid3b* CD8⁺ T cells revealed significant enrichment in pathways related to cytokine–cytokine receptor interactions, JAK–STAT signaling, IL-17 signaling, PI3K–AKT signaling, T cell receptor signaling, and general cytokine-mediated communication (Fig. 3c). These transcriptional findings were validated by RT-qPCR, which confirmed increased expression of *Il2*, *Il10*, *Cd69*, *Cd103*, *Jak2*, *Stat3*, and *Stat5a* in sg-*Arid3b*−1 and sg-*Arid3b*−2 CD8⁺ T cells following tumor antigen exposure (Fig. 3d). Consistent with RNA-seq findings, flow cytometry further confirmed that *Arid3b* knockout decreased TIGIT expression, whereas overexpression of *Arid3b* upregulated TIGIT levels (Supplementary Fig. 3a, b).

Next, to assess whether ARID3B regulates gene expression by remodeling chromatin accessibility, we performed ATAC-seq on *Arid3b*‑knockout and control CD8⁺ T cells under both untreated and OVA peptide‑stimulated conditions (Supplementary Fig. 3c). Strikingly, genome‑wide chromatin accessibility profiles remained largely unchanged upon loss of *Arid3b*, with no broad or drastic shifts in either condition. This result suggests that ARID3B does not function as a global remodeler of chromatin architecture, but rather may repress transcription through more precise mechanisms. For instance, it could recruit modifying enzymes such as KDM4C to locally alter histone modification states (e.g., H3K9me3 and H3K27me3) without substantially altering DNA accessibility^15^.

To investigate the direct transcriptional targets of ARID3B, we performed ChIP-seq in CD8⁺ T cells overexpressing HA-tagged *Arid3b*, using anti-HA or IgG control antibodies, in the presence or absence of OVA peptide stimulation. Heatmap visualization revealed differential binding patterns (Fig. 3e). Genomic annotation of ARID3B binding sites showed dynamic redistribution upon OVA stimulation, with a shift toward promoter regions (41.8%) compared to unstimulated cells, where binding was more evenly distributed among intronic (38.2%), promoter (29.0%), and intergenic (28.7%) regions (Fig. 3f). To link DNA binding with gene regulation, we identified 699 genes with ARID3B-bound promoter regions under antigen-stimulated conditions that also overlapped with DEGs identified by RNA-seq (Fig. 3g). These target genes were further subjected to protein–protein interaction network analysis using the STRING database, providing a molecular framework through which Arid3b modulates transcriptional programs associated with activation, effector function, and memory potential.

### Arid3b represses *Runx3* transcription via direct promoter binding through the ARID domain

**Fig. 4:**
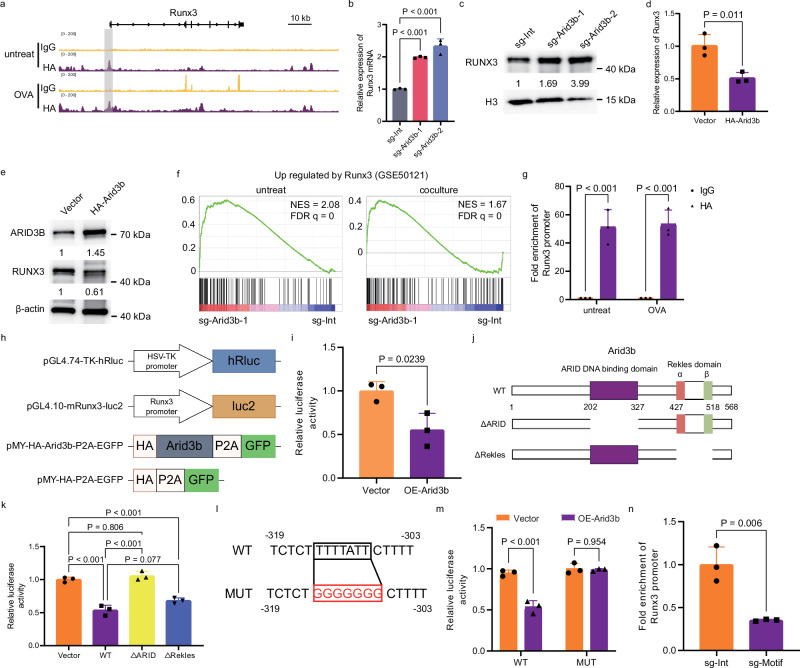
ARID3B directly represses *Runx3* transcription through promoter binding. **a** ChIP-seq tracks showing ARID3B binding at the *Runx3* locus in OT-I; Cas9 CD8⁺ T cells with or without OVA peptide stimulation. **b**, **c** Quantification of *Runx3* mRNA (**b**) and protein (**c**) levels in sg-*Arid3b*-1/2 and sg-Int OT-I; Cas9 CD8⁺ T cells following 4-h OVA stimulation (*n* = 3 biologically independent samples). **d**, **e** Quantification of *Runx3* mRNA (**d**) and protein (**e**) levels in vector and HA–*Arid3b*–overexpressing OT-I; Cas9 CD8⁺ T cells following 4-h OVA stimulation (*n* = 3 biologically independent samples). **f** GSEA showing enrichment of the *Runx3*-upregulated gene signature (from GSE50121) in sg-*Arid3b*-1 versus sg-Int OT-I; Cas9 CD8⁺ T cells based on RNA-seq data. **g** ChIP-qPCR analysis of ARID3B binding at the *Runx3* promoter in vector or HA–*Arid3b*–overexpressing OT-I; Cas9 CD8⁺ T cells (*n* = 3 biologically independent samples). **h** Schematic diagram of the dual-luciferase reporter assay. Created by Figdraw (ID: PWOPRc13aa). **i** Relative luciferase activity of the *Runx3* promoter in 293 T cells transfected with vector or HA-*Arid3b* (*n* = 3 biologically independent samples). **j** Schematic representation of wild-type and truncated ARID3B protein constructs. Created by Figdraw (ID: ITITUaa474). **k** Relative luciferase activity of the *Runx3* promoter in 293T cells transfected with vector, wild-type ARID3B, or truncated ARID3B constructs (*n* = 3 biologically independent samples). **l** Nucleotide sequences of the wild-type and mutant *Runx3* promoter regions used in reporter constructs. **m** Luciferase activity of wild-type or mutant *Runx3* promoter in 293T cells transfected with vector or HA-*Arid3b* (*n* = 3 biologically independent samples). **n** ChIP-qPCR analysis of ARID3B enrichment at the *Runx3* promoter in OT-I; Cas9 CD8⁺ T cells. Cells were transfected with virus expressing either control sgRNAs (sg-Int) or sgRNAs flanking the TTTTATT motif (sg-Motif) (*n* = 3 biologically independent samples). Data are shown as means ± SEM. *P* value was determined by one-way ANOVA with Dunnett’s multiple comparisons (**b**), one-way ANOVA with Tukey’s multiple comparisons (**k**), two-way ANOVA with Sidak’s multiple comparisons (**g**, **m**), or unpaired two-tailed Student *t* test (**d**, **i**, **n**). Source data are provided in Source Data file.

Given the central role of *Runx3* in regulating CD8⁺ T cell differentiation and tissue residency, we next investigated whether *Arid3b* modulates *Runx3* expression. ChIP-seq analysis revealed that HA-tagged ARID3B binds directly to the promoter region of *Runx3* under both unstimulated and OVA-stimulated conditions in CD8⁺ T cells compared with IgG controls (Fig. 4a). To address the potential issue of non-specific antibody binding, we performed ChIP-qPCR under both unstimulated and OVA-stimulated conditions, targeting a housekeeping gene not regulated by Arid3b as a negative control. Consistent with the ChIP-seq data, no significant enrichment of ARID3B was detected at the promoter region of this housekeeping gene (Supplementary Fig. 4a, b). This rules out the possibility of non-specific antibody binding in the ChIP assays.

To validate the functional consequence of this interaction, we examined *Runx3* expression following *Arid3b* perturbation. Under antigen-stimulated conditions, knockout of *Arid3b* using two independent sgRNAs significantly increased both mRNA and protein levels of *Runx3*, while overexpression of *Arid3b* suppressed its expression at both levels (Fig. 4b–e).

To assess the downstream functional relevance of *Runx3* upregulation following *Arid3b* knockout, we leveraged the GSE50121 dataset, which profiles transcriptomic changes in CD8⁺ T cells from wild-type and *Runx3*-deficient mice after IL-2 stimulation. From this dataset, we defined a *Runx3*-upregulated gene signature consisting of 170 genes downregulated upon *Runx3* deletion. Gene set enrichment analysis (GSEA) of our RNA-seq data revealed that this signature was significantly enriched in *Arid3b*-deficient CD8⁺ T cells, regardless of co-culture with tumor cells (Fig. 4f). These results support a model in which *Arid3b* represses *Runx3* expression and its associated transcriptional program.

ChIP-qPCR further confirmed that HA-tagged ARID3B was strongly enriched at the *Runx3* promoter under both basal and OVA-stimulated conditions, with over 50-fold enrichment relative to IgG controls (Fig. 4g). To directly assess the transcriptional effect of ARID3B on the *Runx3* promoter, we constructed a luciferase reporter plasmid (pGL4.10-mRunx3-luc2) containing the murine *Runx3* promoter. Co-transfection of this reporter with an *Arid3b* overexpression construct in 293T cells significantly reduced luciferase activity compared to vector control, indicating that ARID3B represses *Runx3* promoter activity (Fig. 4h, i). To dissect the functional domain responsible for this repression, we generated truncated mutants of ARID3B lacking either the ARID (amino acids 202–327) or the REKLES (amino acids 427–518) domain (Fig. 4j). Dual-luciferase assays revealed that the ARID3B wild-type and ∆REKLES constructs both significantly suppressed *Runx3* promoter activity. In contrast, the ∆ARID mutant failed to exert any repressive effect, and luciferase activity was significantly higher than that of wild-type ARID3B (Fig. 4k). These results indicate that the ARID domain is essential for promoter binding and transcriptional repression of *Runx3*.

Using the JASPAR database^28^, we identified a putative ARID3B-binding motif (TTTTATT) located 314–308 bp upstream of the *Runx3* transcription start site. To validate its functional relevance, we generated a mutant *Runx3* promoter construct in which the TTTTATT motif was replaced with GGGGGGG (Fig. 4l). Dual-luciferase reporter assays showed that ARID3B overexpression significantly suppressed luciferase activity driven by the wild-type promoter but not the mutant construct, confirming that the TTTTATT motif is required for ARID3B-mediated repression of *Runx3* transcription (Fig. 4m). To validate the functional relevance of the TTTTATT motif in a physiological context, we performed CRISPR/Cas9-mediated deletion of this specific element within the endogenous Runx3 promoter in primary CD8⁺ T cells. A plasmid expressing two sgRNAs that flank the TTTTATT motif in the endogenous Runx3 promoter was designed to generate a precise excision. ChIP-qPCR analysis using an anti-HA antibody revealed a significant reduction in ARID3B enrichment at the Runx3 promoter in cells harboring the deleted motif (sg-Motif) compared to control cells (sg-Int) (Fig. 4n). This result demonstrates that the TTTTATT motif located at 314–308 bp upstream of the Runx3 transcriptional start site is essential for ARID3B binding to the endogenous Runx3 promoter in primary CD8⁺ T cells, confirming its role as the functional ARID3B-binding element in a native chromatin and cellular environment.

### Arid3b regulates CD8⁺ T cell tissue residency and antitumor immunity via Runx3

RUNX3 is a well-established transcription factor that promotes cytotoxicity, tissue-residency, and tumor infiltration in CD8⁺ T cells^29–31^. To gain initial insight into the function of *Runx3* in CD8⁺ T cells, we knocked out *Runx3* using two sgRNAs, with knockout efficiency confirmed by western blot analysis (Supplementary Fig. 5a). Following *Runx3* deletion, expression of the activation marker CD69 was reduced (Supplementary Fig. 5b). Upon antigen stimulation, the production of GZMB and IFN-γ was also diminished (Supplementary Fig. 5c, d). Furthermore, *Runx3*-deficient CD8⁺ T cells exhibited impaired cytotoxic activity against CMT93-OVA and MC38-OVA tumor cell lines (Supplementary Fig. 5e, f). Migration assays revealed a significant decrease in the migratory capacity of *Runx3*-knockout CD8⁺ T cells (Supplementary Fig. 5g). To assess whether the observed effects were confounded by alterations in cell viability, we performed Annexin-V and DAPI staining. The results indicated that *Runx3* knockout did not affect the survival of CD8⁺ T cells (Supplementary Fig. 5h). Collectively, these findings demonstrate that *Runx3* plays a critical role in regulating CD8⁺ T cell activation, effector function, cytotoxicity, and migration, independent of effects on cell viability.

**Fig. 5:**
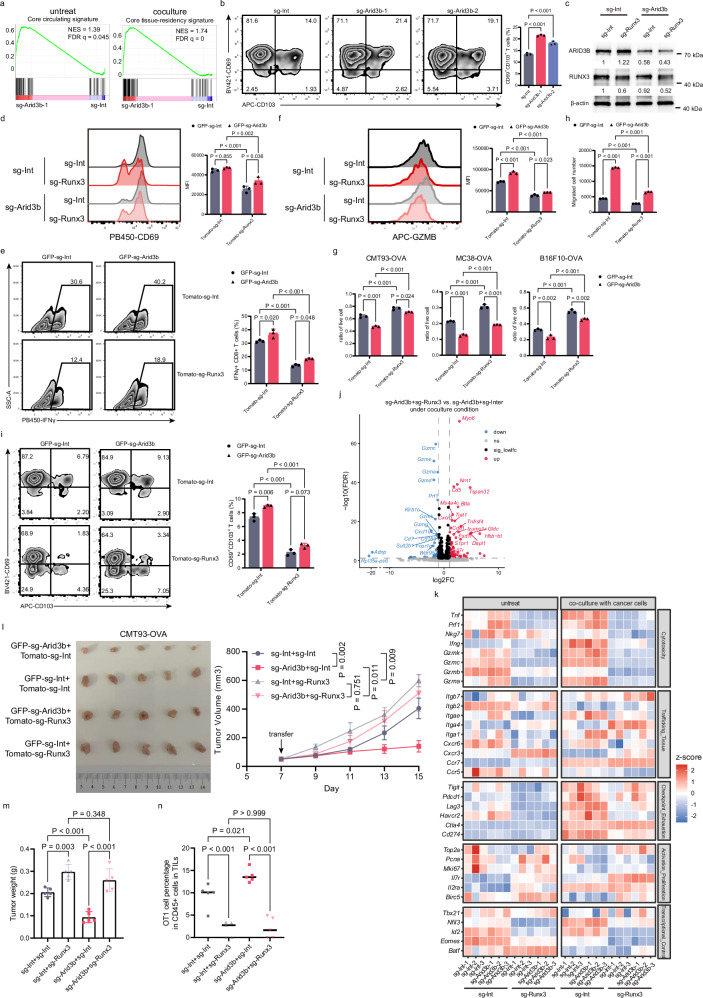
ARID3B regulates CD8⁺ T cell tissue residency and antitumor activity via RUNX3. **a** GSEA of circulating and tissue-resident CD8⁺ T cell gene signatures in sg-*Arid3b*-1 compared to sg-Int CD8⁺ T cells, based on RNA-seq data. **b** Flow cytometric analysis of CD69⁺CD103⁺ CD8⁺ T cells in sg-Int or sg-*Arid3b* group. **c** Western blot analysis of ARID3B and RUNX3 protein levels. **d**, **f** Expression of CD69(d) or GZMB(f) in *Arid3b/Runx3* double‑knockout (DKO), *Arid3b*‑KO, *Runx3*‑KO, and control CD8⁺ T cells. **e** Frequency of IFN-γ⁺ cells among CD8⁺ T cells with *Arid3b* and *Runx3* double knockout. **g** T cell-mediated cytotoxicity assay evaluating the killing capacity of DKO, *Arid3b*‑KO, *Runx3*‑KO, and control OT-I; Cas9 CD8⁺ T cells. E:T = 1:1. **h** Quantification of migrated DKO, *Arid3b*‑KO, *Runx3*‑KO, and control CD8⁺ T cells. **i** Flow cytometric analysis of CD69⁺CD103⁺ populations in DKO, *Arid3b*‑KO, *Runx3*‑KO, and control CD8⁺ T cells. **j** Volcano plot depicting DEGs between DKO and *Arid3b*‑KO CD8⁺ T cells after 4-h co-culture with CMT93-OVA cells. **k** Heatmap of expression levels of genes associated with specified functional programs in DKO, *Arid3b*‑KO, *Runx3*‑KO, and control CD8⁺ T cells. **l**, **m** Representative tumor images and quantification of tumor volume (**l**) and tumor weight (**m**) in *Rag1−/−* mice bearing CMT93-OVA tumors following adoptive transfer of OT-I; Cas9 CD8⁺ T cells with *Arid3b* and *Runx3* double knockout (*n* = 5 mice). **n** Quantification of transferred OT-I; Cas9 CD8⁺ T cells with *Arid3b* and *Runx3* double knockout among tumor-infiltrating lymphocytes (CD45⁺) in CMT93–OVA–bearing mice (*n* = 5 mice). For (**b**, **d**, e–**i**, **k**), *n* = 3 biologically independent samples. Data are shown as means ± SEM. *P* value was determined by one-way ANOVA with Dunnett’s multiple comparisons (**b**), one-way ANOVA with Tukey’s multiple comparisons (**m**, **n**), two-way ANOVA with Tukey’s multiple comparisons (**d**–**i**) or one-tailed two-way ANOVA with correction using Geisser–Greenhouse method (**l**). Source data are provided in Source Data file.

To investigate whether *Arid3b* regulates these key functional features through *Runx3*, we first assessed transcriptional signatures associated with CD8⁺ T cell residency and circulation. Using gene sets defined by Milner et al. (121 genes for tissue-residency; 93 for circulating T cells)^31^, we performed GSEA on RNA-seq data. Under unstimulated conditions, the core circulating signature was modestly enriched in *Arid3b*-deficient CD8⁺ T cells (NES = 1.39, FDR *q* = 0.045). However, following tumor antigen stimulation, the core tissue-resident signature was strongly enriched in sg-*Arid3b* cells (NES = 1.74, FDR *q* = 0), suggesting that *Arid3b* deletion promotes a tissue-resident memory (T_RM)-like phenotype upon antigen engagement (Fig. 5a). Flow cytometric analysis confirmed that *Arid3b*-knockout CD8⁺ T cells exhibited a significantly increased proportion of CD69⁺CD103⁺ T_RM-like cells compared to controls (Fig. 5b).

To determine whether these phenotypic effects were mediated through *Runx3*, we generated CD8⁺ T cells with a double knockout of *Arid3b* and *Runx3*. Western blot analysis confirmed the efficient knockout of both target genes in the respective single- and double-knockout cells (Fig. 5c). Notably, while *Arid3b*-knockout did not significantly elevate CD69 expression compared to control cells, CD69 levels in the double-knockout cells were significantly higher than those in *Runx3*-knockout cells, suggesting that ARID3B can modulate CD69 expression in the absence of RUNX3 (Fig. 5d). Moreover, the elevated IFN-γ production conferred by *Arid3b* deletion was similarly reversed by concurrent *Runx3* knockout (Fig. 5e). This reversal pattern was consistent for GZMB expression (Fig. 5f). Furthermore, the enhanced cytotoxic activity against CMT93-OVA, MC38-OVA, and B16F10-OVA tumor cells, as well as the increased migratory capacity observed in Arid3b-deficient CD8⁺ T cells, were both abrogated upon additional *Runx3* knockout (Fig. 5g, h). Flow cytometry revealed that *Runx3* deletion abolished the increase in CD69⁺CD103⁺ cells observed in the *Arid3b*-knockout setting (Fig. 5i).

To dissect the specific contribution of *Runx3* to the transcriptional program driven by *Arid3b* deficiency, we performed RNA-seq on CD8⁺ T cells under untreated conditions or after 4‑h co‑culture with CMT93‑OVA tumor cells. The experimental groups included *Arid3b/Runx3* double‑knockout, *Arid3b*/*Int*‑knockout (*Arid3b*‑KO), *Runx3*/*Int*‑knockout (*Runx3*‑KO), and *Int*/*Int*‑knockout (control) cells. A volcano plot of DEGs between the *Arid3b*‑KO and double‑knockout groups revealed a broad transcriptional reversal upon additional *Runx3* deletion (Fig. 5j). Strikingly, pathway‑focused analysis demonstrated that the DEGs associated with cytotoxicity, trafficking tissue, checkpoint exhaustion, and activation proliferation, observed in *Arid3b*‑KO cells, were consistently abolished in the double‑knockout group, irrespective of whether cells were co‑cultured with tumor cells or remained untreated (Fig. 5k). These findings indicate that *Runx3* is essential for sustaining the transcriptional rewiring induced by *Arid3b* knockout.

Finally, we evaluated the functional consequence of this axis in vivo. Female *Rag1−/−* mice bearing CMT93-OVA tumors were adoptively transferred with CD8⁺ T cells of indicated genotypes. Compared to controls, *Runx3* deletion significantly impaired tumor control, with increased tumor volume and weight observed in both the sg-Int and sg-*Arid3b* backgrounds (Fig. 5l, m). Flow cytometric analysis of tumor-infiltrating lymphocytes revealed that the frequency of adoptively transferred CD8⁺ T cells was significantly reduced following *Runx3* deletion, even in the context of *Arid3b* deficiency (Fig. 5n).

### ARID3B interacts with RUNX3 to regulate its transcription via domain-specific binding

**Fig. 6:**
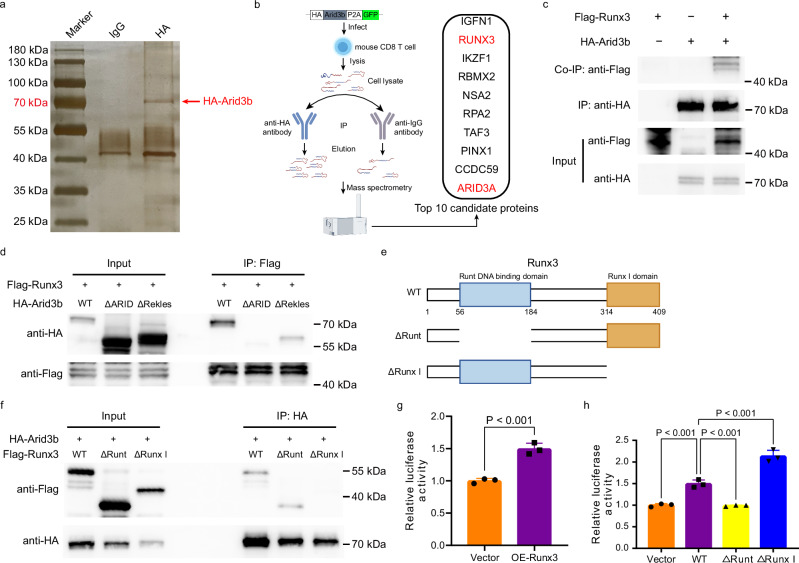
ARID3B interacts with RUNX3 through domain-specific binding. **a** Silver staining of SDS-PAGE–separated proteins immunoprecipitated from CD8⁺ T cells using anti-HA or control IgG antibody. Experiments were repeated three times independently with similar results. **b** Schematic workflow of immunoprecipitation-mass spectrometry (IP/MS) to identify HA-tagged ARID3B-interacting proteins. Top 10 enriched candidate proteins are listed. Created by Figdraw (ID: UPU265d8). **c** Co-immunoprecipitation (Co-IP) demonstrating the physical interaction between HA-tagged ARID3B and Flag-tagged RUNX3 in CD8⁺ T cells. HA antibody is used for the detection of HA-tagged ARID3B. Flag antibody is used for the detection of Flag-tagged RUNX3. Blot was repeated three times with similar results. **d** Co-IP assay showing the interaction between HA-tagged truncated ARID3B constructs and Flag-tagged full-length RUNX3 in CD8⁺ T cells, indicating domain-specific association. HA antibody is used for the detection of HA-tagged full-length and truncated ARID3B. Flag antibody is used for the detection of Flag-tagged RUNX3. Blot was repeated three times with similar results. **e** Schematic representation of full-length and truncated RUNX3 constructs used in Co-IP assays. Created by Figdraw (ID: IWOATf488c). **f** Co-IP demonstrating the interaction between HA-tagged full-length ARID3B and Flag-tagged truncated RUNX3 in CD8⁺ T cells. HA antibody is used for the detection of HA-tagged ARID3B. Flag antibody is used for the detection of Flag-tagged full-length and truncated RUNX3. Blot was repeated three times with similar results. **g** Luciferase reporter assay measuring *Runx3* promoter activity in 293T cells transfected with vector control or *Runx3* overexpression plasmid (*n* = 3 biologically independent samples). **h** Relative luciferase activity of the *Runx3* promoter in 293T cells transfected with vector, wild-type RUNX3, or truncated RUNX3 constructs (*n* = 3 biologically independent samples). Data are shown as means ± SEM. *P* value was determined by one-way ANOVA with Tukey’s multiple comparisons (**g**) or unpaired two-tailed Student *t* test (**h**). Source data are provided in Source Data file.

To further explore the mechanism by which ARID3B regulates *Runx3*, we sought to identify ARID3B-interacting proteins in CD8⁺ T cells. Immunoprecipitation followed by mass spectrometry (IP/MS) revealed *Runx3* as one of the top interactors, ranking second among enriched proteins (Fig. 6a, b). Given our earlier finding that *Arid3b* directly represses *Runx3* transcription, this result raised the possibility of a direct protein–protein interaction between ARID3B and RUNX3.

To validate this interaction, we co-transfected CD8⁺ T cells with HA-tagged *Arid3b* and Flag-tagged *Runx3* expression plasmids. Co-immunoprecipitation (Co-IP) using anti-HA antibodies confirmed the interaction: Flag-RUNX3 co-precipitated with HA-ARID3B, indicating a specific physical association between the two proteins (Fig. 6c).

To identify the domain of ARID3B responsible for this interaction, we generated truncation mutants lacking either the ARID domain or the REKLES domain (Fig. 4j). Co-IP experiments showed that the ARID3B wild-type and ∆REKLES proteins retained the ability to bind RUNX3, whereas deletion of the ARID domain abolished this interaction (Fig. 6d). These findings identify the ARID domain as essential for mediating ARID3B–RUNX3 binding.

We next mapped the corresponding interaction domain within the RUNX3 protein. RUNX3 contains two key functional domains: the N-terminal RUNT domain (amino acids 56–184), which is required for DNA binding, and the C-terminal Runx I domain (amino acids 314–409), which mediates interactions with transcriptional co-regulators. Co-IP experiments using Flag-tagged *Runx3* truncation constructs and HA-ARID3B showed that deletion of the RUNT domain did not affect binding to ARID3B, whereas deletion of the Runx I domain abolished the interaction (Fig. 6e, f). These results indicate that ARID3B binds specifically to the Runx I domain of RUNX3.

Given that ARID3B both represses *Runx3* transcription and physically interacts with the RUNX3 protein, we hypothesized a feedback regulatory mechanism involving RUNX3 autoregulation. Dual-luciferase reporter assays demonstrated that RUNX3 overexpression significantly increased activity of the *Runx3* promoter, suggesting that RUNX3 positively regulates its own transcription (Fig. 6g).

To delineate the contribution of each RUNX3 domain to this autoregulation, we performed luciferase reporter assays using RUNX3 truncation mutants. Deletion of the RUNT domain (*Runx3* ΔRUNT) abolished the enhancement of promoter activity, indicating that DNA binding via the RUNT domain is essential for autoregulation. Interestingly, deletion of the Runx I domain (*Runx3* ΔRunx I) led to a marked increase in promoter activity relative to both wild-type RUNX3 and control, suggesting that the Runx I domain normally serves a repressive role in this feedback loop (Fig. 6h). Taken together, these findings reveal a dual regulatory mechanism in which ARID3B not only represses *Runx3* transcription through promoter binding but also physically interacts with RUNX3 via the ARID–Runx I domain interface.

### Characterization of ARID3B in Human T Cells

**Fig. 7:**
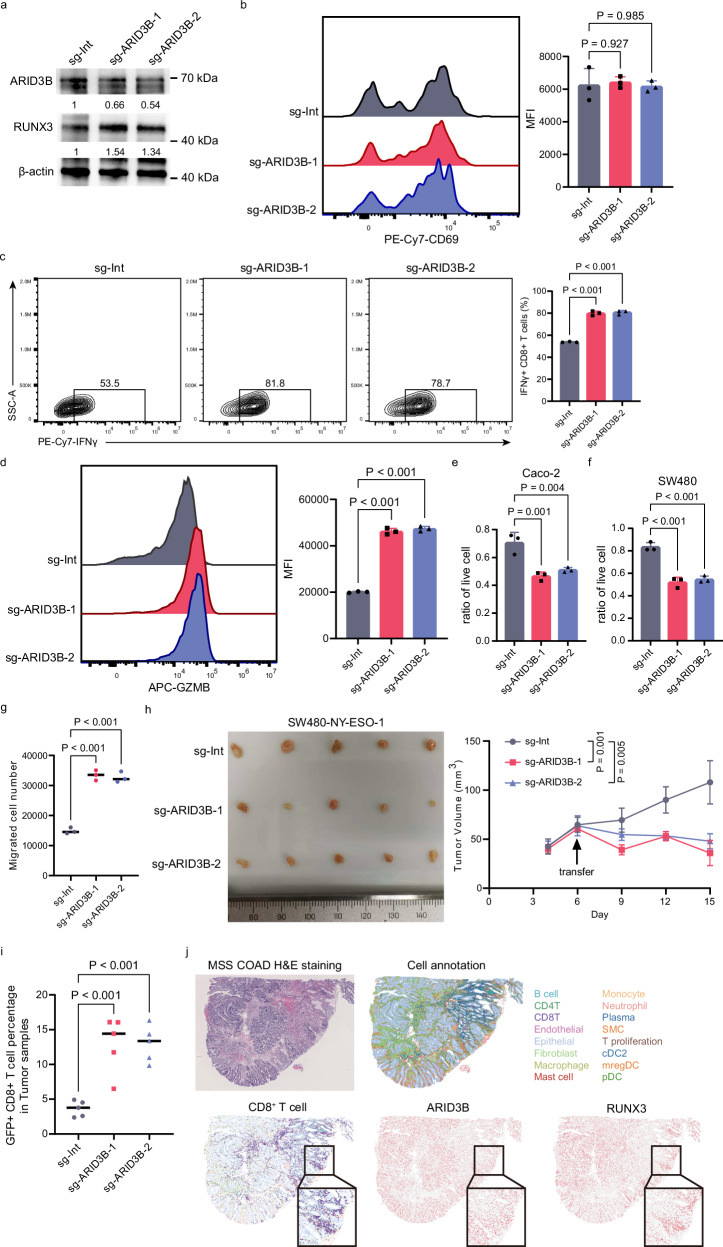
Characterization of *ARID3B* in human CD8^+^ T Cells. **a** Western blot analysis of ARID3B protein levels in human CD8⁺ T cells transduced with control sgRNA (sg-Int) or *ARID3B*-targeting sgRNAs (sg-*ARID3B*-1 or sg-*ARID3B*-2). Blot was repeated three times with similar results. **b** Expression of CD69 in human CD8⁺ T cells transduced with sg-Int or sg-*ARID3B*-1/2, assessed by flow cytometry (*n* = 3 biologically independent samples). **c** Frequency of IFN-γ⁺ cells in human CD8⁺ T cells transduced with sg-Int or sg-*ARID3B*-1/2, assessed by intracellular cytokine staining (n = 3 biologically independent samples). **d** Expression of GZMB in human CD8⁺ T cells transduced with sg-Int or sg-*ARID3B*-1/2, assessed by flow cytometry (*n* = 3 biologically independent samples). **e**, **f** T cell-mediated cytotoxicity assay evaluating the killing capacity of human CD8⁺ T cells transduced with sg-Int or sg-*ARID3B*-1/2 against Caco-2-NY-ESO-1 (**e**), and SW480-NY-ESO-1 (**f**) target cells (*n* = 3 biologically independent samples). E: T = 1:1. **g** Quantification of migrated human CD8⁺ T cells transduced with sg-Int or sg-*ARID3B*-1/2, determined by flow cytometry (*n* = 3 biologically independent samples). **h** Representative tumor image and growth curve of SW480-NY-ESO-1 tumors in NOG mice following adoptive transfer of sg-Int or sg-*ARID3B* human CD8⁺ T cells (*n* = 5 mice). **i** Frequency of adoptively transferred human CD8⁺ T cells in tumor tissues from SW480-NY-ESO-1–bearing mice (*n* = 5 mice). **j** Spatial transcriptomic analysis of ARID3B and RUNX3 expression in CD8⁺ T cells from MSS COAD. Data are shown as means ± SEM. P value was determined by one-way ANOVA with Dunnett’s multiple comparisons (**b**–**g**, **i**) or one-tailed two-way ANOVA with correction using Geisser–Greenhouse method (**h**). Source data are provided in Source Data file.

To investigate the role of *ARID3B* in human CD8⁺ T cells, we first validated efficient *ARID3B* knockout by western blot and observed a concomitant increase in RUNX3 protein expression (Fig. 7a). While *ARID3B* deletion did not affect CD69 expression (Fig. 7b), it elevated the production of both GZMB and IFN-γ (Fig. 7c, d). In co-culture assays using 1G4-overexpressing CD8⁺ T cells and NY-ESO-1–expressing Caco‑2 or SW480 target cells, *ARID3B* knockout significantly enhanced tumor cell killing (Fig. 7e, f). This was further supported by transwell migration assays, which showed that *ARID3B* deficiency promoted CD8⁺ T cell migration in vitro (Fig. 7g).

We next evaluated the in vivo antitumor function of *ARID3B*. Female NOG mice bearing subcutaneous SW480 tumors expressing NY-ESO-1 received adoptive transfer of human PBMCs together with either *ARID3B*-knockout or control CD8⁺ T cells; both CD8⁺ T cell populations were isolated from the same donor PBMCs. As a result, *ARID3B* deletion led to a significant reduction in tumor volume (Fig. 7h) and increased infiltration of CD8⁺ T cells into tumors (Fig. 7i), confirming the role of ARID3B as a key suppressor of CD8⁺ T cell-mediated antitumor immunity in vivo.

Finally, we analyzed publicly available single-cell RNA sequencing data to examine the expression of ARID3B and RUNX3 in human tumor-infiltrating lymphocytes. The results revealed a negative correlation between ARID3B and RUNX3 expression in CD8⁺ T cells (Supplementary Fig. 6a–j). Further subset analysis of CRC TILs from the pan-cancer study showed that ARID3B expression was heterogeneous among human CD8⁺ T cells: it was relatively low in naïve and effector T cells but upregulated in certain functionally specialized T-cell subsets. Notably, Trm exhibited relatively high levels of ARID3B expression^32^ (Supplementary Fig. 6k). In addition, spatial transcriptomic analysis of MSS colorectal cancer samples indicated that, within these CD8⁺ T cells, ARID3B and RUNX3 expression also exhibited a negative correlation (Fig. 7j and Supplementary Fig. 6l).

## Discussion

Microsatellite-stable/proficient mismatch repair (MSS/pMMR) colorectal cancer (CRC) remains a major clinical challenge due to its resistance to immune checkpoint inhibitors (ICIs), a feature tightly linked to the paucity of tumor-infiltrating CD8⁺ T cells within the TME^2–4^. This study addresses a critical gap in our understanding of the molecular mechanisms that limit CD8⁺ T cell infiltration in MSS CRC. Using an in vivo CRISPR/Cas9 screen in a CMT93-based murine model, we identified *Arid3b* as a key T cell-intrinsic suppressor of infiltration and antitumor activity. Genetic ablation of *Arid3b* in CD8⁺ T cells markedly enhanced tumor infiltration and cytotoxic function. Mechanistically, *Arid3b* repressed the transcription of *Runx3* by directly binding a conserved TTTTATT motif in the *Runx3* promoter via its ARID domain. *Runx3* upregulation promoted a tissue-resident memory (T_RM)-like phenotype and effector gene expression, both of which were reversed upon *Runx3* deletion. In addition, we uncovered a protein–protein interaction between ARID3B and RUNX3, suggesting a dual regulatory mechanism involving transcriptional repression and post-translational modulation.

While CRISPR-based functional genomics has previously uncovered regulators of CD8⁺ T cell function—such as *Fli1* and *Regnase-1*—these studies have primarily employed highly immunogenic models like MC38 or B16 melanoma^33,34^. Such models poorly represent the immune-cold TME of MSS CRC. Here, we leveraged a syngeneic model based on the MSS CMT93 line to perform an in vivo CRISPR screen targeting CD8⁺ T cells in an immune-excluded CRC context. The identification of *Arid3b* as a central negative regulator in this setting highlights the necessity of tumor subtype-specific immunogenomic strategies.

*Arid3b* encodes a member of the AT-rich interaction domain (ARID) family of DNA-binding transcription factors, known for roles in chromatin remodeling and transcriptional regulation during development and cancer progression^22,35–37^. This study expands its functional repertoire to include immune regulation, demonstrating that *Arid3b* acts as a transcriptional brake on CD8⁺ T cell activation, infiltration, and tissue residency. Targeting *Arid3b* in CD8⁺ T cells may offer a strategy to overcome the immunotherapy resistance characteristic of MSS CRC.

*Runx3* is a well-established transcription factor critical for CD8⁺ T cell development, cytotoxic function, and tissue-resident memory differentiation^29–31^. RUNX3 directly activates genes encoding IFN-γ, granzymes, and perforin, thereby orchestrating effector responses. Importantly, epigenetic repression of *Runx3* through promoter methylation limits its expression in exhausted or dysfunctional T cells, a barrier partially reversible by DNA hypomethylating agents like decitabine^38^. However, systemic demethylation therapies like decitabine carry risks of off-target toxicity due to genome-wide effects. Our finding that ARID3B directly represses *Runx3* transcription provides a more precise alternative to such epigenetic reprogramming. Targeting the ARID3B-RUNX3 axis allows for transcriptional reactivation of *Runx3* without causing global chromatin remodeling, thereby offering the potential for a superior safety profile.

Notably, prior studies have shown that ARID3B can form complexes with ARID3A and the histone demethylase KDM4C to modulate chromatin states via H3K9me3 demethylation^15^. In our IP-MS analysis, we confirmed interaction between ARID3B and ARID3A, but did not detect KDM4C, suggesting context-specific differences in complex formation. More strikingly, we identified a interaction between ARID3B and RUNX3, which occurs via the ARID domain of ARID3B and the Runx I domain of RUNX3. These results suggest that ARID3B may exert transcriptional repression not only through DNA binding but also by physically interacting with RUNX3 to limit its transactivation potential, possibly by recruiting corepressors or obstructing coactivator access.

Our mechanistic delineation of the ARID3B-Runx3 axis nominates ARID3B as a compelling therapeutic target with two primary translational paths. First, in the realm of cell therapy, our findings provide a strong rationale for engineering *ARID3B*-deficient CAR-T cells. Using CRISPR-Cas9 gene editing to disrupt *ARID3B* during the ex vivo manufacturing process represents a straightforward strategy to intrinsically program therapeutic T cells for enhanced Runx3-driven tissue residency, persistence, and effector function against solid tumors. Second, from a drug discovery perspective, the defined DNA-binding activity of ARID3B suggests its potential as a druggable target. Future efforts could focus on high-throughput screening to identify small-molecule inhibitors that disrupt its interaction with cognate promoters, offering a scalable pharmacological intervention. We envision that both strategies, cellular engineering and pharmacological inhibition, could be powerfully combined with immune checkpoint blockers to simultaneously potentiate T-cell intrinsic function and overcome extrinsic immunosuppression in the tumor microenvironment.

Beyond *Arid3b*, our in vivo CRISPR screen identified other top hits, including *Znf131* and *Grk2*, which may regulate CD8⁺ T cell function through distinct mechanisms. Our functional validation shows that the ablation of *Znf131*, a BTB/POZ-zinc finger transcription factor, enhances the secretion of IFNγ and GZMB and cytotoxic activity in CD8⁺ T cells. GRK2 (G protein-coupled receptor kinase 2) is a key kinase that desensitizes G protein-coupled receptors (GPCRs), including chemokine receptors critical for immune cell trafficking^39^. In T cells, GRK2-mediated phosphorylation of receptors like CXCR4 can terminate migratory signals. Notably, high GRK2 expression correlates with poorer prognosis in colon adenocarcinoma. Within the TME, elevated GRK2 activity could therefore dampen chemokine-driven recruitment of CD8⁺ T cells, contributing to the “cold” phenotype observed in MSS CRC. Future work is needed to investigate the precise mechanisms by which these genes function.

Despite these insights, we acknowledge several limitations in our study. First, we relied on subcutaneous tumor models rather than orthotopic models, which better mimic the native tumor microenvironment of colorectal cancer. Second, our study utilized OVA-specific T cells rather than polyclonal T cells, which more accurately reflect physiological anti-tumor T-cell responses. Third, while we focused on the ARID3B-RUNX3 regulatory axis, ARID3B is likely to regulate additional targets in CD8⁺ T cells that have not yet been elucidated. Fourth, we did not explore combination therapies, such as ARID3B knockdown together with anti-PD-1 treatment, which would help evaluate its potential to sensitize tumors to immune checkpoint inhibition. Finally, although the adoptive transfer model of *Arid3b*-KO CD8⁺ T cells provided functional evidence, our study lacks validation using a CD8⁺ T cell–specific conditional knockout mouse model (e.g., CD8(E8I)-Cre; Arid3b^flox/flox^). Such a model would allow for precise, cell-intrinsic assessment of Arid3b function in CD8⁺ T cells across physiological and tumor-bearing contexts within an intact immune system.

In summary, our study identifies *Arid3b* as a pivotal transcriptional repressor that limits CD8⁺ T cell infiltration and function in immune-cold CRC. Mechanistically, ARID3 B binds the *Runx3* promoter and physically interacts with the RUNX3 protein, constituting a dual-layered regulatory axis. By releasing this molecular brake, *Arid3b* deletion reprograms CD8⁺ T cells toward a highly functional, tumor-infiltrating, and tissue-resident phenotype. These findings not only deepen our understanding of immunosuppressive barriers in MSS CRC but also nominate *Arid3b* as a promising target for precision immunotherapy. Future studies should investigate the translational potential of ARID3B modulation and its integration into combination treatment regimens to enhance ICI responsiveness in resistant tumor subtypes.

## Methods

### Cell lines and mice

The CMT93, MC38, B16F10, and 293T cell lines, were gifts from Pan lab (Deng Pan, Tsinghua University), which were cultured in DMEM supplemented with 10% fetal bovine serum (FBS) and 1% penicillin/streptomycin. Caco-2 and SW480 cells, maintained in our laboratory, were cultured in DMEM supplemented with 10% or 20% FBS, respectively. All the cell lines were incubated at 37 °C with 5% CO₂.

Mice, both sexes, between the ages of 6–8 weeks of age were used for the study. OT-I; Cas9 and Rag1^−/−^ mice were gifted from Pan lab^40,41^. WT female C57BL/6 mice (NO. N000013) were purchased from the GemPharmatech, Nanjing, China. Female NOG mice (NO. 408) were purchased from the Charles River, Beijing, China. Animals of the same sex were randomly assigned to experimental groups. All mice were kept in a specific-pathogen-free facility in the Animal Resource Center at Peking University. Mice were euthanized using cervical dislocation. All animal work was approved by the Institutional Animal Care and Use Committee (IACUC) of Peking University and performed with approved protocols (AAIS-Zengzx-01).

### Isolation, transduction, and culture of mouse CD8^+^ T cell

Primary OT-1; Cas9 CD8^+^ T cells were isolated from spleens and peripheral lymph nodes of OT-1; Cas9 mice using the MojoSort™ Mouse CD8^+^ T Cell Isolation Kit according to the manufacturer’s protocol. Isolated cells were cultured in complete RPMI-1640 medium (containing 10% fetal bovine serum, 1% penicillin/streptomycin, 1% non-essential amino acids, 20 mM HEPES, 1 mM sodium pyruvate, and 0.05 mM 2-mercaptoethanol) supplemented with 1 μg/mL anti-mouse CD28 and 5 μg/mL anti-mouse CD3 antibodies for 24 h. T cells were then infected with concentrated retrovirus in complete RPMI-1640 medium supplemented with 20 ng/mL of recombinant mouse IL-2. Two days post-transduction, T cells were sorted by flow cytometry. Subsequently, T cells were cultured in complete RPMI-1640 medium supplemented with 20 ng/mL IL-2.

### Isolation, transduction, and culture of human CD8^+^ T cell

Human CD8^+^ T cells were isolated from commercial peripheral blood mononuclear cells (PBMCs) purchased from Biosource. On Day 1, frozen PBMCs were thawed and resuscitated in complete RPMI-1640 medium, supplemented with 10% fetal bovine serum, 1% penicillin/streptomycin, 1% non-essential amino acids, 1% GlutaMAX, 1 mM sodium pyruvate, and 10 ng/mL recombinant human IL-2. After 24 h of culture, PBMCs were collected, and human CD8^+^ T cells were isolated using the EasySep^TM^ Human CD8^+^ T Cell Isolation Kit according to the manufacturer’s instructions. The isolated CD8^+^ T cells were activated for 24 h in complete RPMI-1640 medium containing 5 µg/mL anti-human CD3, 1 µg/mL anti-human CD28, and 5 µg/mL Fibronectin. Following activation, the T cells were infected with concentrated lentiviral vectors in fresh complete medium. Two days post-infection, successfully infected T cells were sorted by flow cytometry. Subsequently, the sorted CD8^+^ T cells were maintained in complete RPMI-1640 medium at a concentration of 1 × 10^6^ cells/ml. The culture was sustained by performing a half-medium replenishment daily and passaging the cells every two days to maintain optimal cell density and viability.

### Library design and CRISPR screen target selection

Candidate genes for the CRISPR screen were selected through an integrated, multi-step strategy. First, genes with robust expression in CD8⁺ T cells were defined using single‑cell RNA‑seq data from The Human Protein Atlas portal (www.proteinatlas.org)^42,43^. Genes expressed in >90% of CD8⁺ T cells and ranking within the top 1500 by transcript abundance were retained. To ensure coverage of key regulators, all transcription factors annotated in the UniProt database were included regardless of expression level, given their central role in gene regulatory networks even at low abundance^44^. Further, membrane‑protein genes, immune‑related functional genes (annotated via the ImmPort Shared Data^45^), and genes differentially expressed upon CD8⁺ T‑cell activation (based on GEO datasets) were incorporated. In order to mitigate potential survival bias, essential genes and housekeeping genes annotated in the DepMap database were systematically excluded^46^. All selected human genes were mapped to their mouse orthologs. For each target gene, four high‑efficiency sgRNAs were designed using the CRISPick algorithm^47,48^. Additionally, 500 non‑targeting control sgRNAs (sg‑Intergenic#1–500) targeting intergenic regions were included. All sgRNA sequences were subjected to BLAT alignment to confirm the absence of off‑target homology. The final library consisted of 2236 genes and 9444 sgRNAs. The sequences of non‑targeting control sgRNAs were listed in Supplementary Data 1.

### In vivo CRISPR/Cas9 screening

Retrovirus was generated by co-transfecting 293 T cells with PMYs retroviral library plasmids and packaging plasmids^49^. In brief, PMYs library plasmid and packaging plasmids were co-transfected at a 1:1 ratio into low-passage 293T cells at 80% confluency in 15-cm tissue culture dishes. Viral supernatant was collected at 48 h and 72 h post-transfection, filtered using a 0.45-μm polyethersulfone syringe filter, and concentrated using PEG8000. The concentrated supernatant was aliquoted and stored at −80 °C until use. At 48 h after transfection, viral supernatants were harvested, concentrated, and frozen at −80 °C. The MSS murine colorectal cancer cell line CMT93-OVA was subcutaneously implanted into Rag1^−/−^ mice to establish xenograft tumors. On the same day, OT1; Cas9 CD8^+^ T cells were isolated from the spleens and peripheral lymph nodes of OT1; Cas9 mice. Twenty-four hours post-isolation, OT1; Cas9 CD8^+^ T cells were transduced with retrovirus at a multiplicity of infection of 0.3. After 48 h of culture, T cells expressing GFP were sorted by flow cytometry and expanded in vitro. On day 7 post-tumor implantation, 1 × 10^6^ transduced OT1; Cas9 CD8^+^ T cells were saved as input. In total, 5 × 10^6^ transduced OT1; Cas9 CD8^+^ T cells per mouse were adoptively transferred via tail vein into CMT93-OVA tumor-bearing Rag1^−/−^ mice. Seven days post-transfer, subcutaneous tumors were excised, dissociated into single-cell suspensions, and flow cytometry was performed to isolate GFP^+^ CD8^+^ T cells, representing adoptively transferred OT1; Cas9 CD8^+^ T cells. Genomic DNA from sorted GFP^+^ cells and input reference cells was subjected to sequencing. Sequencing data were analyzed using the MAGeCK algorithm to identify differentially enriched sgRNAs.

### Tumor studies

Tumor sizes were measured every 2–3 days with a vernier caliper. Tumor volumes were calculated as (length × width × width)/2. Ethical endpoints were set at tumor length reaching 20 mm. The maximal tumor size was not exceeded.

### Gene knockout and overexpression

Two sgRNAs with the highest enrichment fold of *Arid3b* and *Znf131* in the CRISPR library were selected to knock out the expression of *Arid3b* in mouse CD8^+^ T cells. Additionally, two sgRNAs each for knocking out *Runx3* in mouse CD8^+^ T cells and *ARID3B* in human CD8^+^ T cells were designed using CRISPOR^50^. For overexpression, plasmids carrying the *Arid3b* gene were constructed and transfected into mouse CD8^+^ T cells. The protein levels of ARID3B, ZNF131 and RUNX3 were assessed by western blot to confirm the knockout efficiency and overexpression levels.

The sgRNA number and sequence were as follows.

sg-Int: AGAGATGAGACAAACTACCC

sg-Arid3b-1: TGATGGGAATCCGGTTGATT

sg-Arid3b-2: TCCATCAGCATCGTCACTCC

sg-Znf131-1: AGAACCTGTGGAAATTGAGG

sg-Znf131-2: AGCAAAACTAATGATACAAG

sg-Runx3-1: GGCAAGATGGGCGAGAACAG

sg-Runx3-2: TCCATCCGGCACATCCCCCA

sg-ARID3B-1: AGTGTTTGAACGGGGCAACA

sg-ARID3B-2: AATTGATGGCAACCGCAGGG.

### Western blot

Whole cell protein extracts were prepared by lysing T cells treated with or without OVA peptide for 4 h using RIPA lysis buffer. Cell lysates were denatured by boiling at 100 °C for 5 min and loaded onto 7.5–12.5% SDS-PAGE gels before transferring to polyvinylidene difluoride (PVDF) membranes. Membranes were blocked with 5% non-fat powdered milk in Tris-buffered saline containing 0.1% Tween-20 (TBST) for 1 h at room temperature, followed by overnight incubation with primary antibodies at 4 °C under gentle agitation. After washing with TBST, membranes were incubated with horseradish peroxidase (HRP)-conjugated secondary antibodies for 1 h at room temperature. Protein bands were visualized using a ChemiDoc™ Imaging System (Bio-Rad Laboratories) with enhanced chemiluminescence substrates. The following antibodies were used: ARID3B (Bethyl Laboratory#A302-564A, 1:2000 dilution), β-actin (Proteintech#20536-1-AP, 1:5000 dilution), RUNX3 (Abmart#T55395, 1:2000 dilution), H3 (CST#4499, 1:5000 dilution), Flag (Proteintech#66008-4-Ig, 1:5000 dilution), HA (CST#3724, 1:5000 dilution), and ZNF131 (ABclonal#A15331, 1:2000 dilution).

### T-cell-mediated killing assay

Cancer cells were seeded into 12-well plates at a density of 2 × 10^5^ cells per well and allowed to adhere overnight. CD8^+^ T cells were then added to the tumor cells at effector to target (E: T) ratios ranging from 1:1 to 1:9. Following 24–48 h of co-culture, cancer cells were harvested and analyzed by flow cytometry. Dead cells were stained with DAPI.

### Flow cytometry analysis

Flow cytometry was used extensively to analyze the proportion of adoptively transferred CD8^+^ T cells in tumor-infiltrating lymphocytes, the proportion of CD69^+^CD103^+^ cells, IFN-γ^+^ T cells, and the expression levels of various markers such as CD69, GZMB, TIM3, and TIGIT in different experimental groups. The used antibodies are as follows: APC-anti-mouse-CD3 (BioLegend#100235), PE/Cyanine7-anti-mouse-CD8a (BioLegend#100721), Brilliant Violet 421-anti-mouse-CD45 (BioLegend#103133), PE-anti-mouse-Granzyme B (BioLegend#372214), Brilliant Violet 421-anti-mouse-CD69 (BioLegend#104527), Brilliant Violet 421-anti-mouse-IFN-γ (BioLegend#505829), APC-anti-mouse-CD103 (BioLegend#110905), PE-anti-mouse-TIGIT (Vstm3) (BioLegend#156103), Brilliant Violet 421-anti-mouse-CD366 (TIM-3) (BioLegend#134019), PE/Cyanine7-anti-human-CD69 (BioLegend#310911), PE/Cyanine7-anti-human-IFN-γ (BioLegend#506517), APC-anti-human/mouse-Granzyme B (BioLegend#372203), APC-Annexin-V (BioLegend#640919). All antibodies were used at 1:200 dilution. Gating strategies for all flow experiments are shown in Supplementary Fig. S7.

### T cell migration assay

For the migration assay, the supernatant of CMT93-OVA cells was placed in the lower chamber of a 5-μm transwell, and Arid3b-knockout or control OT1; Cas9 CD8^+^ T cells were added to the upper chamber. After 24 h, the number of CD8^+^ T cells in the lower chamber was counted. In the competitive migration assay, sg-Int CD8^+^ T cells carrying tdTomato (sg-Int-Tomato) were used as the control, mixed in equal proportions with either sg-Int CD8^+^ T cells carrying GFP-labeled control (sg-Int-GFP) or Arid3b-knockout cells (sg-Arid3b-1-GFP) for the transwell experiment. The ratio of tdTomato to GFP in the lower chamber was counted to evaluate the migration ability. For in vivo validation, mixed populations of sg-Int-GFP/sg-Int-Tomato or sg-Arid3b-1-GFP/sg-Int-Tomato CD8^+^ T cells were adoptively transferred into CMT93-OVA tumor-bearing mice, and tumor-infiltrating lymphocytes were analyzed by flow cytometry.

### RNA-seq

To investigate transcriptional alterations in CD8^+^ T cells under antigen-stimulated and basal conditions, RNA-seq profiling was performed on sg-Int and sg-Arid3b CD8^+^ T cells co-cultured with or without CMT93-OVA tumor cells at a ratio of 1:1, with 3 samples in each group. After 4 h of co-culture, CD8^+^ T cells were isolated via flow cytometric sorting. A minimum of 1 × 10^6^ cells per sample were collected across all groups. Total RNA was extracted from purified CD8^+^ T cells using standard protocols and subjected to quality control. High-quality RNA samples were subsequently delivered to Annoroad Gene Technology for library preparation and paired-end sequencing on an Illumina platform.

### RT-qPCR

Total RNA was extracted from T cells using TRIzol reagent (Invitrogen) following the manufacturer’s protocol. The concentration and purity of RNA were assessed by NanoDrop 2000. First-strand cDNA was synthesized from 1 μg RNA using the Evo M-MLV RT Kit (Accurate Biology) with random hexamer primers according to the manufacturer’s guidelines. qPCR amplification was performed in triplicate using SYBR Green Supermix (Bio-Rad). Relative gene expression was calculated via the ΔΔCt method, normalized to Actb (β-actin) as an endogenous control. Primer sequences used in qPCR are detailed in Supplementary Table 1.

### ATAC-seq

ATAC‑seq libraries were constructed from CD8⁺ T cells (either *Arid3b*‑knockout or control, with or without OVA antigen stimulation) using the TruePrep DNA Library Prep Kit V2 for Illumina (Vazyme, TD501) according to the manufacturer’s instructions. In brief, 50,000 viable cells per condition were washed once with cold PBS and lysed in cold ATAC‑seq lysis buffer (10 mM Tris‑HCl pH 7.4, 10 mM NaCl, 3 mM MgCl₂, 0.1% IGEPAL CA‑630) for 10 min to isolate nuclei. Nuclei were pelleted by centrifugation at 500×*g* for 5 min at 4 °C. The pelleted nuclei were resuspended in 50 μL of tagmentation mix and incubated at 37 °C for 30 min. Tagmented DNA was purified using VAHTS DNA Clean Beads (Vazyme, N411). Library amplification was performed with the TruePrep Index Kit V4 for Illumina (Vazyme, TD204) under the following thermal cycling conditions: 72 °C for 3 min; 98 °C for 30 s; followed by 12–15 cycles of 98 °C for 15 s, 60 °C for 30 s, and 72 °C for 30 s. Amplified libraries were purified again with VAHTS DNA Clean Beads and subjected to quality control by Annoroad Gene Technology before paired‑end sequencing on an Illumina platform. ATAC-seq reads were processed with the ENCODE ATAC-seq pipeline (v2.2.2). After QC and adapter trimming, reads were aligned to the GRCm38 genome to generate BAM files, and peaks were identified with Genrich in ATAC-seq mode.

### Chromatin immunoprecipitation assay

ChIP-seq was performed using HA or the corresponding IgG antibody on HA-tagged Arid3b-overexpressing CD8^+^ T cells under untreated or treated with OVA antigen peptide for 4 h conditions. Differential binding peaks were visualized by heatmap, and genomic annotation of peaks was carried out. The intersection of differential binding sites in the two conditions was analyzed, followed by motif analysis and KEGG enrichment analysis. ChIP-qPCR analysis was conducted using ChIP DNA to detect whether Arid3b protein binds to the promoter of the *Runx3* gene or the *Gapdh* gene.

The primer sequence used in ChIP-qPCR were as follows.

Runx3-F: TTCTAAGGGGCCGTGACATC

Runx3-R: AGGATGCAAGAAGCCAGCTC

Gapdh-F: GGAGGTGAAGCAGGCTCAAT

Gapdh-R: TCCTGTCCCATTGTCAAGCC.

### Dual-luciferase reporter assay

The pGL4.10-mRunx3-luc2 plasmid, in which the transcription of firefly luciferase was driven by the promoter of the mouse *Runx3* gene, was constructed. This plasmid was co-transfected into 293T cells with the pGL4.74-TK-hRluc plasmid and either the Arid3b-overexpression plasmid or the control plasmid for the dual-luciferase reporter assay. Protein truncated plasmids of HA-tagged ARID3B and Flag-tagged RUNX3, or the Runx3 promoter mutant plasmids, were also constructed and used in the dual-luciferase reporter assay to analyze the relative luciferase activity and determine the functional domains and binding elements. The HA or Flag tag is expressed at the N‑terminus of ARID3B or RUNX3, while the EGFP or tdTomato fluorescent protein is connected to the C‑terminus via a P2A linker.

### Immunoprecipitation-mass spectrometry (IP-MS)

Immunoprecipitation was performed using Dynabeads^TM^ Protein A Immunoprecipitation kit (ThermoFisher, CAT#10006D). Protein A magnetic beads (40 µL per sample) were incubated with 1 µg of anti-HA or control IgG antibody in binding buffer for 2 h at 4 °C with rotation to allow antibody conjugation. Cell pellets (>1 × 10^7^ cells) were lysed on ice for 30 min in lysis buffer supplemented with 1× protease/phosphatase inhibitor cocktail. Lysates were clarified by centrifugation at 15,000×* g* for 10 min at 4 °C, and 10% of the supernatant was retained as the input control. The remaining lysate was equally divided and incubated with HA- or IgG-conjugated beads for 3 h at 4 °C with rotation. Beads were washed three times with wash buffer, and bound proteins were eluted in 2× loading buffer followed by boiling at 95 °C for 5 min. 5 µL eluted proteins were loaded on SDS-PAGE gel and visualized via silver staining to confirm enrichment of HA-Arid3b prior to mass spectrometry analysis.

### Kyoto Encyclopedia of Genes and Genomes (KEGG) pathway enrichment analysis

KEGG pathway enrichment analysis was conducted on differentially upregulated genes identified between sg-Int and sg-Arid3b groups under tumor co-culture conditions, using the “enrichKEGG” function of the “clusterProfiler” R package^51^. Differentially upregulated genes were identified by the criteria of *P* < 0.05 and log2Fold Change ≥0.4.

### Gene set enrichment analysis (GSEA)

GSEA was performed to evaluate whether predefined gene sets exhibited statistically significant differences between sg-Arid3b and sg-Int groups using GSEA 4.3.3 software^52^. In this study, we employed the RUNX3-upregulated gene signature derived from GSE50121, along with core circulating and tissue-residency signatures defined by Milner et al.^31,53^.

### Statistical analyses

Two-way Analysis of variance (ANOVA) was used to analyze the tumor growth data. For comparisons between two groups, unpaired two-tailed Student *t* test was used. Alternatively, for comparisons involving multiple groups, one-way or two-way ANOVA was conducted, followed by Dunnett’s, Sidak’s, or Tukey’s multiple comparisons. Statistical analyses were conducted on data presented as mean ± SEM. R 4.4.1 and GraphPad Prism 10 were utilized for all graphical representations and statistical calculations. Statistical significance was determined at a *P* value less than 0.05.

### Reporting summary

Further information on research design is available in the Nature Portfolio Reporting Summary linked to this article.

## Supplementary information

Supplementary Information

Description of Additional Supplementary Files

Supplementary Data 1

Reporting Summary

Transparent Peer Review file

## Source data

Source Data

## Data Availability

The genomic and proteomic data generated in this study are publicly available as follows: The mass spectrometry proteomics data have been deposited to the ProteomeXchange Consortium (https://proteomecentral.proteomexchange.org) via the iProX partner repository^54,55^ with the dataset identifier PXD077233. The RNA-seq and ChIP-seq data generated in this study have been deposited in the Gene Expression Omnibus (GEO) under accession numbers GSE304035, GSE304750, and GSE328207. Source data are provided with this paper.

## Code availability

The code used for CRISPR/Cas9 screen analysis, RNA-seq analysis, and ChIP-seq analysis in this study is available from GitHub (https://github.com/Steve-Luo55/MSS_crispr_screen_paper).
