# Supplementary material for: Arid3b suppresses CD8 + T cell infiltration and function in microsatellite-stable colorectal cancer via Runx3
Source: Nat Commun. 2026 May 15;17:6448. doi: 10.1038/s41467-026-73241-7 (PMC13376159; doi:10.1038/s41467-026-73241-7)
Supplement: Supplementary file 1 — Supplementary Information [file 41467_2026_73241_MOESM1_ESM.pdf]

**Supplementary Information**

# Arid3b suppresses CD8+ T cell infiltration and function in Microsatellite-stable colorectal cancer via Runx3

Shuo Wang[1,2,†], Sen Hou[1,2,†], Ce Luo[3,†], Haorui Zhang[4], Yiteng Jin[3], Rui Zhang[4], Yanping Zhao[5], Xiaoyu Xiong[4], Rui Guo[3], Chao Wang[1,2], Yudi Bao[1,2], Liang Wen[6], Deng Pan[7], Yingjiang Ye[1,2,*], Zexian Zeng[3,4,8,*], Zhidong Gao[1,2,*]

[1]Department of Gastroenterological Surgery, Peking University People's Hospital, Beijing 100044, China

[2]Laboratory of Surgical Oncology, Peking University People's Hospital, Beijing 100044, China

[3]Center for Quantitative Biology, Academy for Advanced Interdisciplinary Studies, Peking University, Beijing 100084, China

[4]Peking-Tsinghua Center for Life Sciences, Academy for Advanced Interdisciplinary Studies, Peking University, Beijing 100084, China

[5]Tsinghua-Peking Center for Life Sciences, Tsinghua University, Beijing 100084, China

[6]Department of Obstetrics and Gynecology, Seventh Medical Center of Chinese PLA General Hospital, Beijing 100010, China

[7]Tsinghua-Peking Center for Life Sciences, Department of Basic Medical Sciences, Tsinghua University, Beijing 100084, China

[8]Peking University Chengdu Academy for Advanced Interdisciplinary Biotechnologies, Chengdu, Sichuan 610213, China

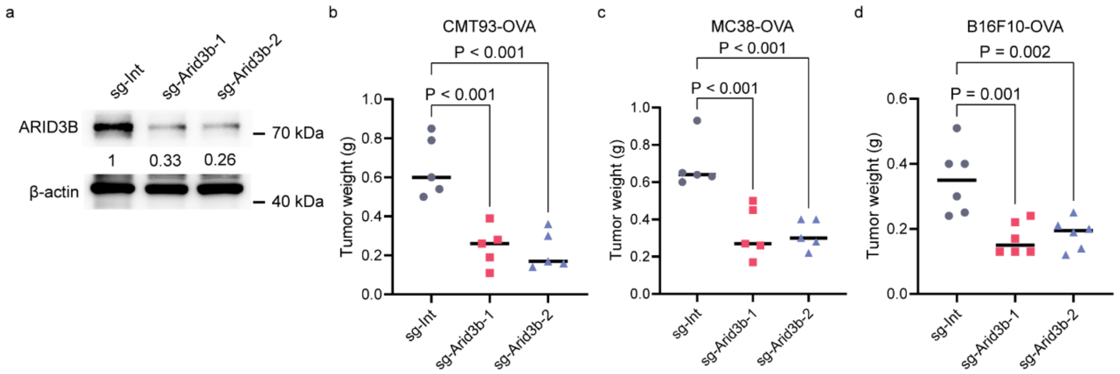


**Supplementary Figure 1. Loss of *Arid3b* enhances CD8⁺ T cell–mediated antitumor immunity.** (Related to Figure 1)

(a) Western blot analysis of ARID3B protein levels in OT-I; Cas9 $CD8^+$ T cells transduced with control sgRNA (sg-Int) or *Arid3b*-targeting sgRNAs (sg-*Arid3b*-1 or sg-*Arid3b*-2). Blot was repeated three times with similar results.

(b, c) Tumor weights of CMT93-OVA (b) and MC38-OVA (c) tumors in *Rag1*$^{-/-}$ mice following adoptive transfer of sg-Int or sg-*Arid3b* OT-I; Cas9 $CD8^+$ T cells (n = 5 mice).

(d) Tumor weight of B16F10-OVA tumors in wild-type C57BL/6 mice following adoptive transfer of sg-Int or sg-*Arid3b* OT-I; Cas9 $CD8^+$ T cells (n = 6 mice).

Data are presented as means ± SEM. P value was determined by one-way ANOVA with Dunnett's multiple comparisons (b, c, d). Source data are provided in Source Data file.

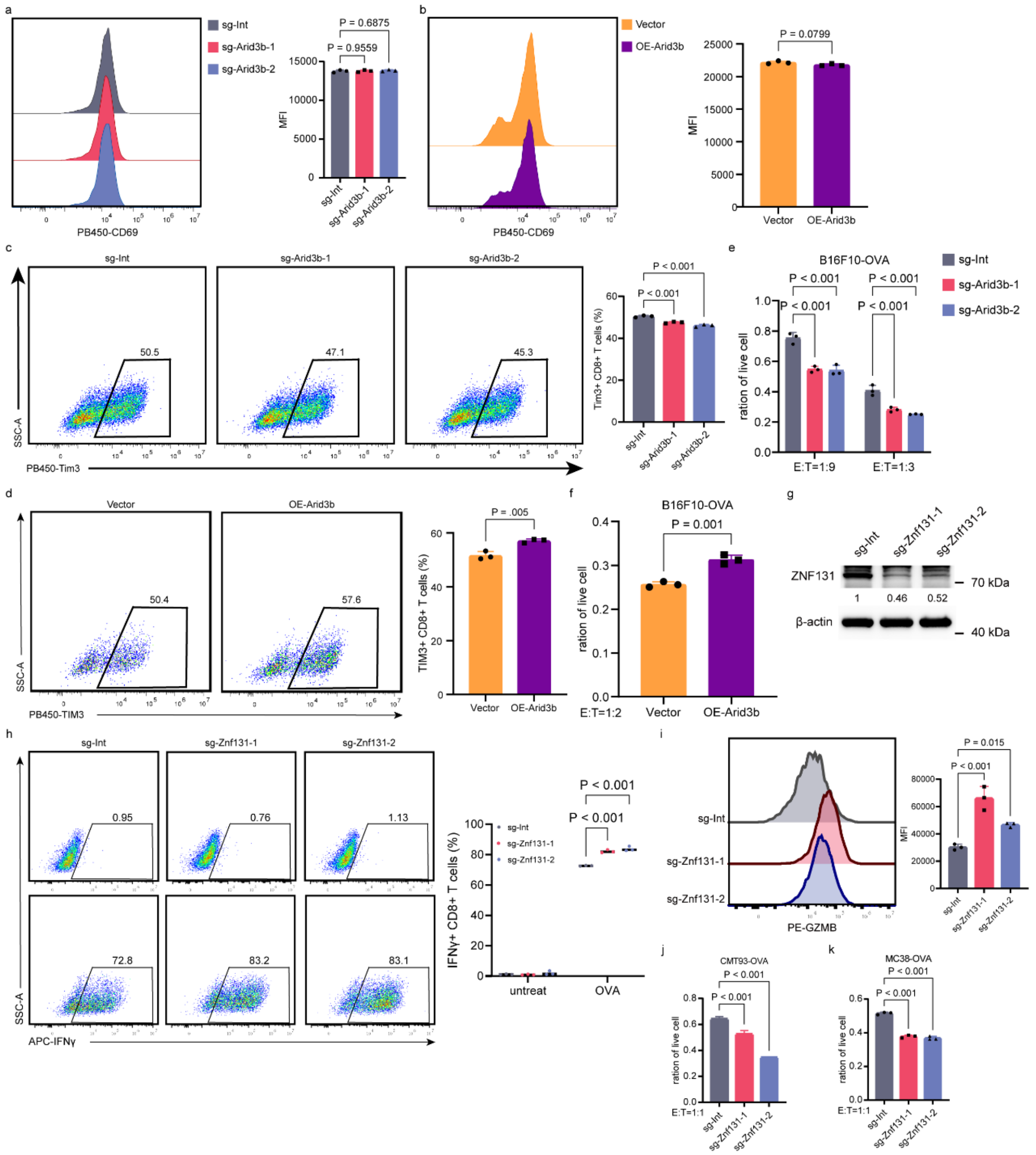


**Supplementary Figure 2. Immunological characterization of *Arid3b* and *Znf131* in mouse CD8$^+$ T cells** (Related to Fig. 2)

(a, b) Expression of CD69 in CD8$^+$ T cells following *Arid3b* knockout (a) or overexpression (b), assessed by flow cytometry. (n = 3 biologically independent samples).

(c, d) Frequency of TIM3$^+$ cells in CD8$^+$ T cells following *Arid3b* knockout (c) or overexpression (d), assessed by flow cytometry. (n = 3 biologically independent samples).

(e, f) T cell–mediated cytotoxicity assay evaluating the killing capacity of CD8$^+$ T cells against B16F10-OVA target cells following *Arid3b* knockout (e) or overexpression (f) (n = 3 biologically independent samples). E : T denotes the effector-to-target cell ratio.

(g) Western blot analysis of ZNF131 protein levels in OT-I; Cas9 CD8$^+$ T cells transduced with control sgRNA (sg-Int) or *Znf131*-targeting sgRNAs (sg-*Znf131*-1 or sg-*Znf131*-2).

(h) Flow cytometric analysis of IFN-γ production in OT-I; Cas9 $CD8^+$ T cells transduced with control sg-Int or sg-*Znf131*-1/2, with or without OVA peptide stimulation (n = 3 biologically independent samples).
(i) Expression of GZMB in OT-I; Cas9 $CD8^+$ T cells transduced with control sg-Int or sg-*Znf131*-1/2, assessed by flow cytometry (n = 3 biologically independent samples).
(j, k) T cell–mediated cytotoxicity assay evaluating the killing capacity of OT-I; Cas9 $CD8^+$ T cells against MC38-OVA (j) and CMT93-OVA (k) target cells following *Znf131* knockout (n = 3 biologically independent samples). E : T denotes the effector-to-target cell ratio.
Data are shown as means ± SEM. P value was determined by one-way ANOVA with Dunnett's multiple comparisons (a, c, e, i, j, k), unpaired two-tailed Student t-test (b, d, f) or two-way ANOVA with Sidak's multiple comparisons (h). Source data are provided in Source Data file.

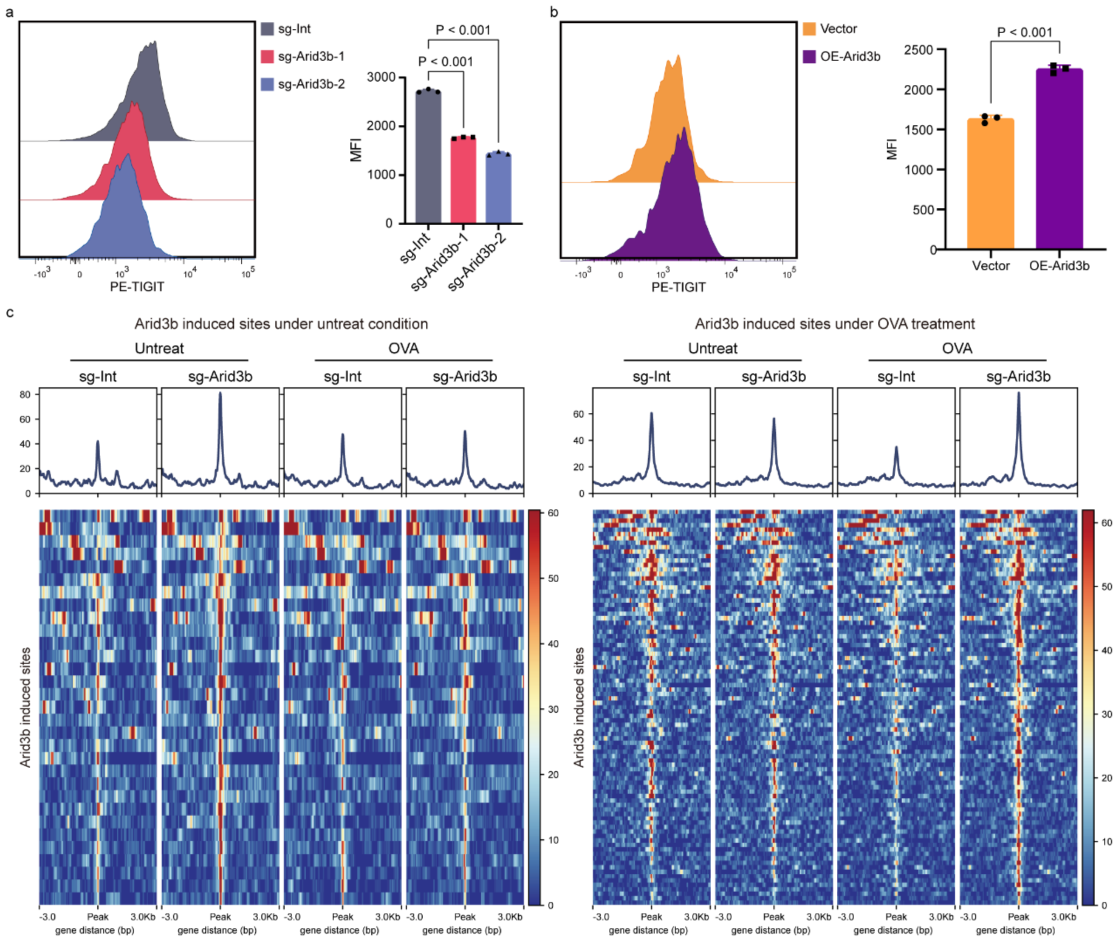


**Supplementary Figure 3. Additional analysis of Aird3b** (Related to Fig. 3)

(a, b) Expression of TIGIT in $CD8^+$ T cells following *Arid3b* knockout (a) or overexpression (b), assessed by flow cytometry (n = 3 biologically independent samples).

(c) Heatmap of ATAC-seq showing chromatin accessibility of Arid3b induced sites, defined under either untreated or OVA treatment conditions, across all experimental groups.

Data are shown as means ± SEM. P value was determined by one-way ANOVA with Dunnett's multiple comparisons (a) or unpaired two-tailed Student t-test (b). Source data are provided in Source Data file.

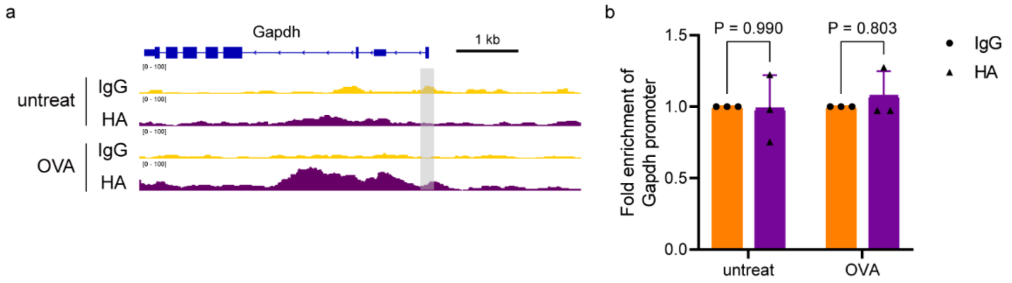


**Supplementary Figure 4. Additional analysis of ChIP-seq data** (Related to Fig. 4)
(a) ChIP-seq tracks showing ARID3B can not bind at the *Gapdh* locus in OT-I; Cas9 $CD8^+$ T cells treated with control IgG or anti-HA antibody, with or without OVA peptide stimulation.
(b) ChIP-qPCR analysis of ARID3B binding at the *Gapdh* promoter in vector or HA–*Arid3b*–overexpressing OT-I; Cas9 $CD8^+$ T cells, with or without OVA treatment (n = 3 biologically independent samples).
Data are shown as means ± SEM. P value was determined by two-way ANOVA with Sidak's multiple comparisons (b). Source data are provided in Source Data file.

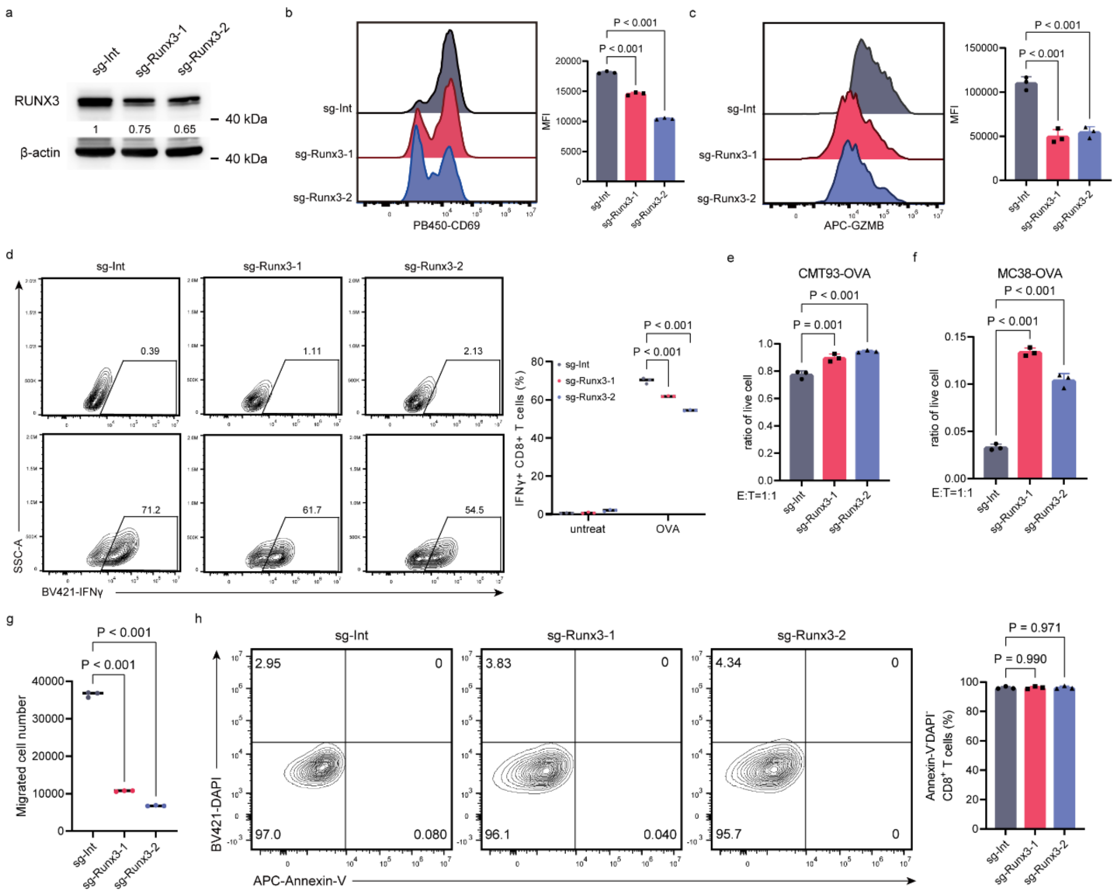


**Supplementary Figure 5. The knockout of *Runx3* suppresses mouse CD8⁺ T cell effector function and migration.**

(a) Western blot analysis of RUNX3 protein levels in OT-I; Cas9 CD8⁺ T cells transduced with control sgRNA (sg-Int) or *Runx3*-targeting sgRNAs (sg-*Runx3*-1 or sg-*Runx3*-2).

(b) Expression of CD69 in OT-I; Cas9 CD8⁺ T cells transduced with sg-Int or sg-*Runx*3-1/2, assessed by flow cytometry (n = 3 biologically independent samples).

(c) Expression of GZMB in OT-I; Cas9 CD8⁺ T cells transduced with sg-Int or sg-*Runx*3-1/2, assessed by flow cytometry (n = 3 biologically independent samples).

(d) Frequency of IFN-γ⁺ cells in OT-I; Cas9 CD8⁺ T cells transduced with sg-Int or sg-*Runx*3-1/2, assessed by intracellular cytokine staining (n = 3 biologically independent samples).

(e, f) T cell–mediated cytotoxicity assay evaluating the killing capacity of OT-I; Cas9 CD8⁺ T cells transduced with sg-Int or sg-*Runx*3-1/2 against CMT93-OVA (e), and MC38-OVA (f) target cells (n = 3 biologically independent samples). E : T = 1:1.

(g) Quantification of migrated OT-I; Cas9 CD8⁺ T cells transduced with sg-Int or sg-*Runx*3-1/2, determined by flow cytometry (n = 3 biologically independent samples).

(h) Frequency of Annexin-V⁻DAPI⁻ cells in OT-I; Cas9 CD8⁺ T cells transduced with sg-Int or sg-*Runx*3-1/2, assessed by flow cytometry (n = 3 biologically independent samples).

Data are shown as means ± SEM. P value was determined by one-way ANOVA with Dunnett's multiple comparisons (b, c, e, f, g, h) or two-way ANOVA with Sidak's multiple

comparisons (d). Source data are provided in Source Data file.

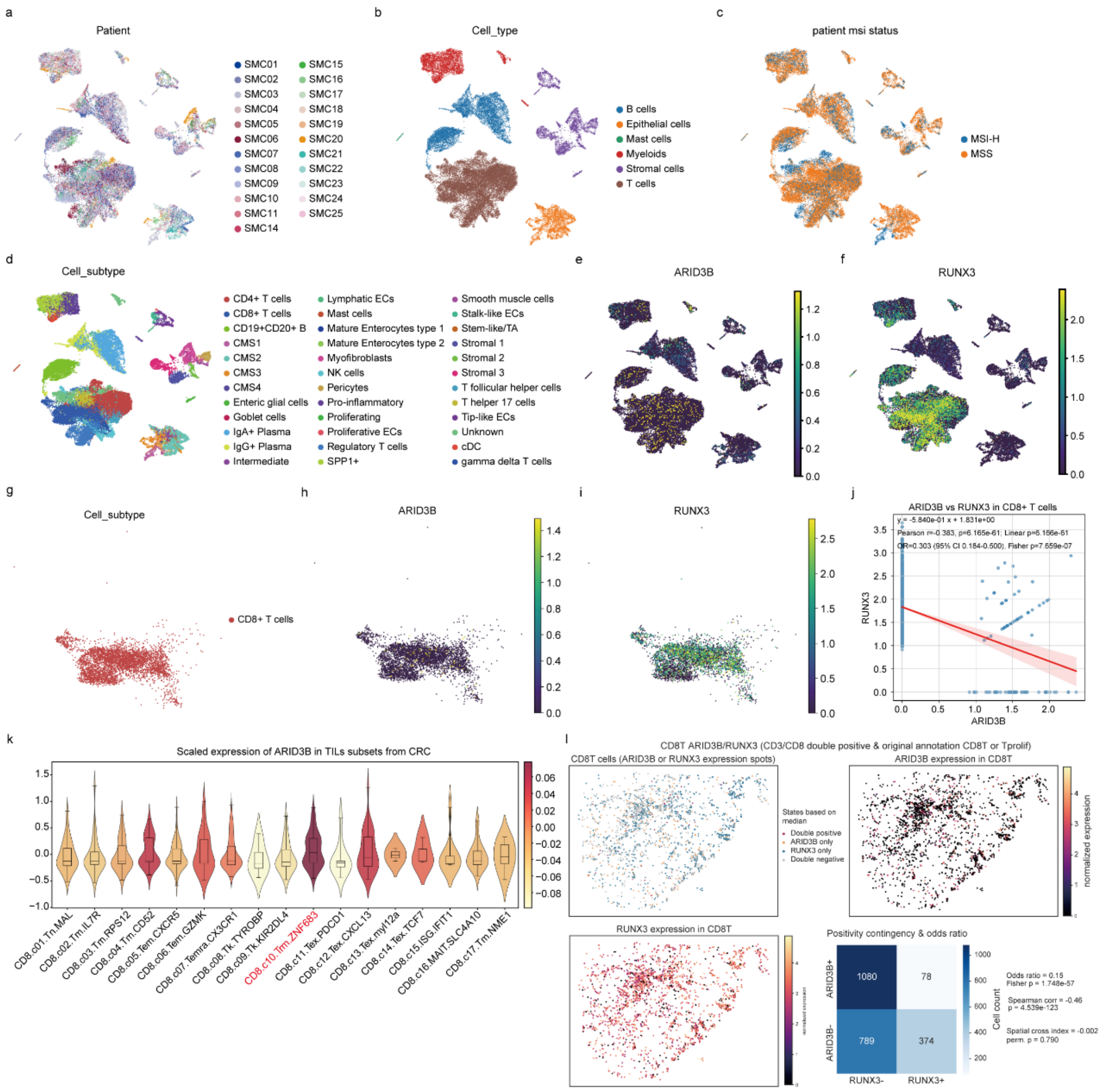


**Supplementary Figure 6. Additional analysis of human ARID3B and RUNX3** (Related to Fig. 7)

(a-d) Uniform Manifold Approximation and Projection (UMAP) plot of patient (a), cell type (b), MSI status (c), and cell subtype (d).

(e, f) The expression of ARID3B (e) and RUNX3 (f) in all cell subtypes.

(g) UMAP plot of CD8$^{+}$ T cells.

(h, i) The expression of ARID3B (h) and RUNX3 (i) in CD8$^{+}$ T cells.

(j) Correlation analysis of ARID3B and RUNX3 expression in CD8$^{+}$ T cells with nonzero expression of ARID3B or RUNX3.

(k) Scaled expression of ARID3B in tumor-infiltrating CD8$^{+}$ T cell subsets from CRC.

(l) Spatial analysis in MSS CRC revealed a negative association between ARID3B and RUNX3 in CD8+ T cells. CD8$^{+}$ T cells were identified from the original CD8T or Tprolif annotations. Using the median expression of ARID3B and RUNX3 within CD8$^{+}$ T cells as thresholds, spots were classified as double-positive, ARID3B-only, RUNX3-only, or double-negative. ARID3B and RUNX3 expression in CD8+ T cells were visualized, and odds ratios, Spearman correlations, and a spatial cross-correlation index were computed with corresponding P values.

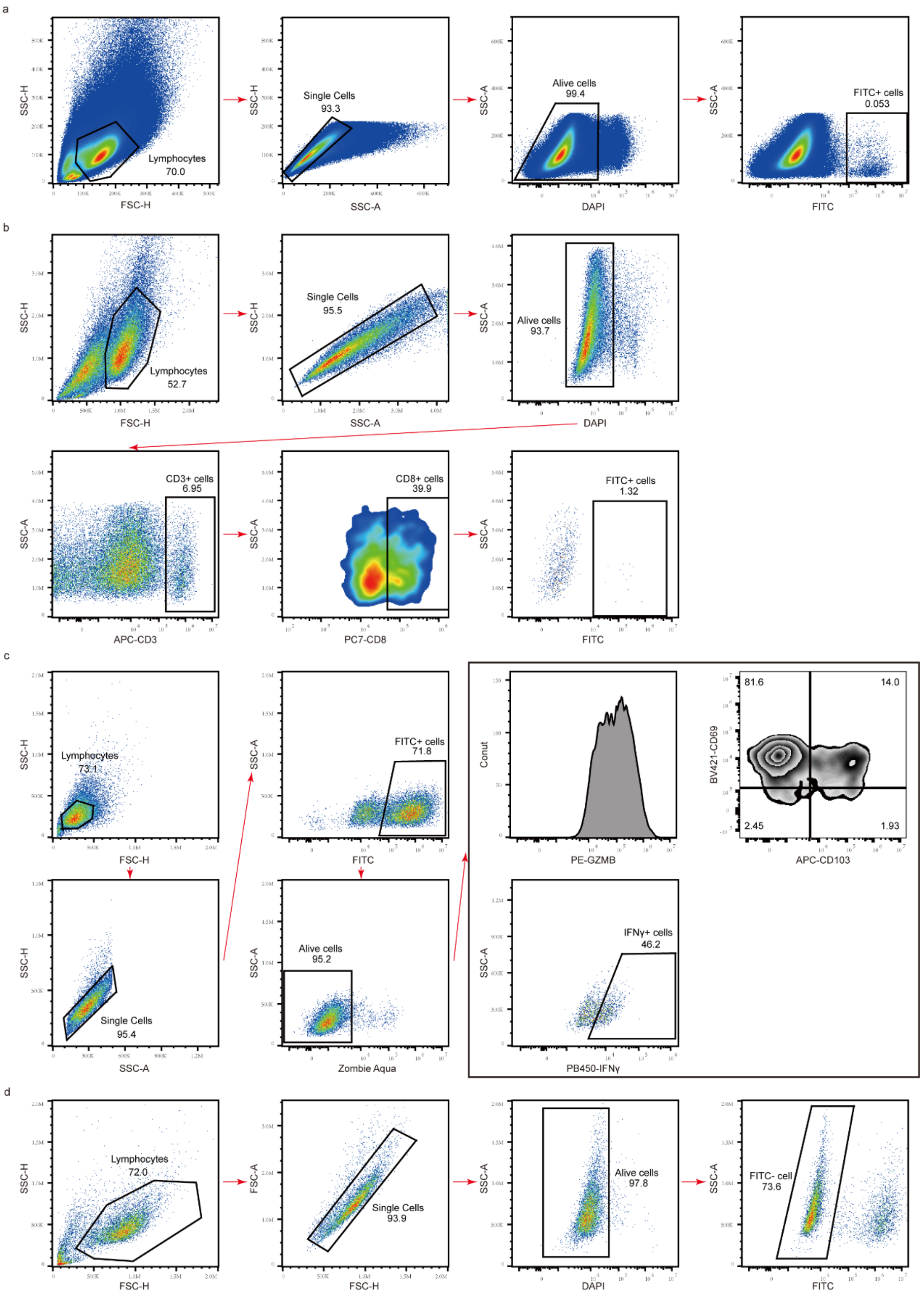


**Supplementary Figure 7. The gating strategy in this study.**

(A) The gating strategy for in vivo CRISPR screening.

(B) The gating strategy for detecting the proportion of adoptively transferred $CD8^+$ T cells in tumor samples or among tumor-infiltrating lymphocytes *in vivo*. For B16F10-OVA tumor-bearing mice, the frequency of adoptively transferred $GFP^+$ $CD8^+$ T cells within the tumor-infiltrating $CD8^+$ cells ($CD3^+CD8^+$) is measured. For CMT93-OVA or

MC38-OVA tumor-bearing mice, the proportion of adoptively transferred GFP$^{+}$ CD8$^{+}$ T cells among tumor-infiltrating lymphocytes (CD45$^{+}$) is determined. For NOG mice, the percentage of adoptively transferred GFP$^{+}$ CD8$^{+}$ T cells within the tumor sample is assessed.

(C) The gating strategy for *in vitro* assays to detect surface markers or cytokine secretion in CD8$^{+}$ T cells.

(D) The gating strategy for T-cell-mediated killing assay.

Supplementary Table 1 The primer sequence used in qPCR

| Primer name | Primer sequence |
|---|---|
| Actb-F | 5’-CGTTGACATCCGTAAAGACC-3’ |
| Actb-R | 5’-AACAGTCCGCCTAGAAGCAC-3’ |
| Cd69-F | 5’-CTGGTGAACTGGAACATTGGATTGG-3’ |
| Cd69-R | 5’-CCTCACAGTCCACAGCGGTAAC-3’ |
| Cd103-F | 5’-ATGTGGACTCTGGTGACTACTGTAG-3’ |
| Cd103-R | 5’-TGCCATCTTCCTCCTCGTCTTC-3’ |
| Il2-F | 5’-AGCAGGATGGAGAATTACAGGAACC-3’ |
| Il2-R | 5’-AATCCAGAACATGCCGCAGAGG-3’ |
| Il10-F | 5’-TGGACAACATACTGCTAACCGACTC-3’ |
| Il10-R | 5’-GCCGCATCCTGAGGGTCTTC-3’ |
| Jak2-F | 5’-GCTTGTGGTATTACGCCTGTGTATC-3’ |
| Jak2-R | 5’-TGCCTGGTTGACTCGTCTATGTG-3’ |
| Runx3-F | 5’-CGCAACGCTTCCGCTGTC-3’ |
| Runx3-R | 5’-AAACTCTTCCCTCGCCCACTG-3’ |
| Stat3-F | 5’-AATCTCAACTTCAGACCCGCCAAC-3’ |
| Stat3-R | 5’-GCTCCACGATCCTCTCCTCCAG-3’ |
| Stat5a-F | 5’-AGTCGGTGACGGAGGAGAAGTTC-3’ |
| Stat5a-R | 5’-CGGTGGCAGTAGCATTGTGGTC-3’ |
