# Supplementary material for: Arid3b suppresses CD8 + T cell infiltration and function in microsatellite-stable colorectal cancer via Runx3
Source: Nat Commun. 2026 May 15;17:6448. doi: 10.1038/s41467-026-73241-7 (PMC13376159; doi:10.1038/s41467-026-73241-7)
Supplement: Supplementary file 2 — Description of Additional Supplementary Files [file 41467_2026_73241_MOESM2_ESM.pdf]

Description of Additional Supplementary Files

Supplementary Data 1. The sequences of non-targeting control sgRNAs used in CRISPR/Cas9 library screen.
