# Supplementary material for: Arid3b suppresses CD8 + T cell infiltration and function in microsatellite-stable colorectal cancer via Runx3
Source: Nat Commun. 2026 May 15;17:6448. doi: 10.1038/s41467-026-73241-7 (PMC13376159; doi:10.1038/s41467-026-73241-7)
Supplement: Supplementary file 4 — Reporting Summary [file 41467_2026_73241_MOESM4_ESM.pdf]

nature portfolio

Corresponding author(s): Zhidong Gao

Last updated by author(s): Apr 15, 2026

Reporting Summary

Nature Portfolio wishes to improve the reproducibility of the work that we publish. This form provides structure for consistency and transparency in reporting. For further information on Nature Portfolio policies, see our Editorial Policies and the Editorial Policy Checklist.

Statistics

For all statistical analyses, confirm that the following items are present in the figure legend, table legend, main text, or Methods section.

n/a	Confirmed	
☐	☒	The exact sample size (*n*) for each experimental group/condition, given as a discrete number and unit of measurement
☐	☒	A statement on whether measurements were taken from distinct samples or whether the same sample was measured repeatedly
☐	☒	The statistical test(s) used AND whether they are one- or two-sided<br>*Only common tests should be described solely by name; describe more complex techniques in the Methods section.*
☒	☐	A description of all covariates tested
☐	☒	A description of any assumptions or corrections, such as tests of normality and adjustment for multiple comparisons
☐	☒	A full description of the statistical parameters including central tendency (e.g. means) or other basic estimates (e.g. regression coefficient) AND variation (e.g. standard deviation) or associated estimates of uncertainty (e.g. confidence intervals)
☐	☒	For null hypothesis testing, the test statistic (e.g. *F*, *t*, *r*) with confidence intervals, effect sizes, degrees of freedom and *P* value noted<br>*Give P values as exact values whenever suitable.*
☒	☐	For Bayesian analysis, information on the choice of priors and Markov chain Monte Carlo settings
☒	☐	For hierarchical and complex designs, identification of the appropriate level for tests and full reporting of outcomes
☒	☐	Estimates of effect sizes (e.g. Cohen's *d*, Pearson's *r*), indicating how they were calculated

Our web collection on statistics for biologists contains articles on many of the points above.

Software and code

Policy information about availability of computer code

Data collection	GSEA 4.3.3, R 4.4.1 and GraphPad Prism 10 were utilized for data analysis.
Data analysis	Code for CRISPR screen, RNA-seq, and ChIP-seq analysis is available at https://github.com/Steve-Luo55/MSS_crispr_screen_paper

For manuscripts utilizing custom algorithms or software that are central to the research but not yet described in published literature, software must be made available to editors and reviewers. We strongly encourage code deposition in a community repository (e.g. GitHub). See the Nature Portfolio guidelines for submitting code & software for further information.

Data

Policy information about availability of data

All manuscripts must include a data availability statement. This statement should provide the following information, where applicable:

- Accession codes, unique identifiers, or web links for publicly available datasets
- A description of any restrictions on data availability
- For clinical datasets or third party data, please ensure that the statement adheres to our policy

The genomic and proteomic data generated in this study are publicly available as follows: The mass spectrometry proteomics data have been deposited to the ProteomeXchange Consortium (https://proteomecentral.proteomexchange.org) via the iProX partner repository with the dataset identifier PXD077233 [https://proteomecentral.proteomexchange.org/cgi/GetDataset?ID=PXD077233]. The RNA-seq and ChIP-seq data generated in this study have been deposited in the Gene Expression Omnibus (GEO) under accession numbers GSE304035 [https://www.ncbi.nlm.nih.gov/geo/query/acc.cgi?acc=GSE304035], GSE304750 [https://

www.ncbi.nlm.nih.gov/geo/query/acc.cgi?acc=GSE304750], and GSE328207 [https://www.ncbi.nlm.nih.gov/geo/query/acc.cgi?acc=GSE328207]. Source data are provided with this paper.

Research involving human participants, their data, or biological material

Policy information about studies with human participants or human data. See also policy information about sex, gender (identity/presentation), and sexual orientation and race, ethnicity and racism.

Reporting on sex and gender	*Use the terms sex (biological attribute) and gender (shaped by social and cultural circumstances) carefully in order to avoid confusing both terms. Indicate if findings apply to only one sex or gender; describe whether sex and gender were considered in study design; whether sex and/or gender was determined based on self-reporting or assigned and methods used. Provide in the source data disaggregated sex and gender data, where this information has been collected, and if consent has been obtained for sharing of individual-level data; provide overall numbers in this Reporting Summary. Please state if this information has not been collected. Report sex- and gender-based analyses where performed, justify reasons for lack of sex- and gender-based analysis.*
Reporting on race, ethnicity, or other socially relevant groupings	*Please specify the socially constructed or socially relevant categorization variable(s) used in your manuscript and explain why they were used. Please note that such variables should not be used as proxies for other socially constructed/relevant variables (for example, race or ethnicity should not be used as a proxy for socioeconomic status). Provide clear definitions of the relevant terms used, how they were provided (by the participants/respondents, the researchers, or third parties), and the method(s) used to classify people into the different categories (e.g. self-report, census or administrative data, social media data, etc.) Please provide details about how you controlled for confounding variables in your analyses.*
Population characteristics	*Describe the covariate-relevant population characteristics of the human research participants (e.g. age, genotypic information, past and current diagnosis and treatment categories). If you filled out the behavioural & social sciences study design questions and have nothing to add here, write "See above."*
Recruitment	*Describe how participants were recruited. Outline any potential self-selection bias or other biases that may be present and how these are likely to impact results.*
Ethics oversight	*Identify the organization(s) that approved the study protocol.*

Note that full information on the approval of the study protocol must also be provided in the manuscript.

Field-specific reporting

Please select the one below that is the best fit for your research. If you are not sure, read the appropriate sections before making your selection.

☒ Life sciences ☐ Behavioural & social sciences ☐ Ecological, evolutionary & environmental sciences

For a reference copy of the document with all sections, see nature.com/documents/nr-reporting-summary-flat.pdf

Life sciences study design

All studies must disclose on these points even when the disclosure is negative.

Sample size	For the subcutaneous tumor formation assay in mice, 5 or 6 mice were used per group.<br>For other types of experiments, n = 3 was chosen.
Data exclusions	Data were not excluded from analysis.
Replication	All replication attempts were successful.
Randomization	Age matched mice were allocated randomly to treatment or control group.
Blinding	Investigators were not blinded for RNA-seq, ATAC-seq, and ChIP-seq, but the analysis pipelines were applied identically to all datasets.<br>Tumor volume and tumor weight was measured by an operator blinded to the treatment or control group.

Reporting for specific materials, systems and methods

We require information from authors about some types of materials, experimental systems and methods used in many studies. Here, indicate whether each material, system or method listed is relevant to your study. If you are not sure if a list item applies to your research, read the appropriate section before selecting a response.

Materials & experimental systems

n/a	Involved in the study	
☐	☒	Antibodies
☐	☒	Eukaryotic cell lines
☒	☐	Palaeontology and archaeology
☐	☒	Animals and other organisms
☒	☐	Clinical data
☒	☐	Dual use research of concern
☒	☐	Plants

Methods

n/a	Involved in the study	
☐	☒	ChIP-seq
☐	☒	Flow cytometry
☒	☐	MRI-based neuroimaging

Antibodies

Antibodies used	Anti-ARID3B (Bethyl Laboratories, A302-564A), used at 1:2000<br>Anti-RUNX3 (Abmart, T55395), used at 1:2000<br>Anti-β-actin (Proteintech, 20536-1-AP), used at 1:5000<br>Anti-H3 (Proteintech, 4499), used at 1:5000<br>Anti-Flag (Cell Signaling Technology, 66008-4-Ig), used at 1:5000<br>Anti-HA (Cell Signaling Technology, 3724), used at 1:5000<br>Anti-Znf131 (ABclonal, A15331), used at 1:2000<br>APC-anti-mouse-CD3 (BioLegend, 100235), used at 1:200<br>PE/Cyanine7-anti-mouse-CD8a (BioLegend, 100721), used at 1:200<br>Brilliant Violet 421-anti-mouse-CD45 (BioLegend, 103133), used at 1:200<br>PE-anti-mouse-Granzyme B (BioLegend, 372214), used at 1:200<br>Brilliant Violet 421-anti-mouse-CD69 (BioLegend, 104527), used at 1:200<br>Brilliant Violet 421-anti-mouse-IFN-γ (BioLegend, 505829), used at 1:200<br>APC-anti-mouse-CD103 (BioLegend, 110905), used at 1:200<br>PE-anti-mouse-TIGIT (Vstm3) (BioLegend, 156103), used at 1:200<br>Brilliant Violet 421-anti-mouse-CD366 (TIM-3) (BioLegend, 134019), used at 1:200<br>PE/Cyanine7-anti-human-CD69 (BioLegend#310911), used at 1:200<br>PE/Cyanine7-anti-human-IFN-γ (BioLegend#506517), used at 1:200<br>APC-anti-human/mouse-Granzyme B (BioLegend#372203), used at 1:200
Validation	All antibodies were validated by the manufacturers.

Eukaryotic cell lines

Policy information about cell lines and Sex and Gender in Research

Cell line source(s)	CMT93 cells (mouse, gifts from Pan lab (Deng Pan, Tsinghua University))<br>MC38 cells (mouse, gifts from Pan lab (Deng Pan, Tsinghua University))<br>B16F10 cells (mouse, gifts from Pan lab (Deng Pan, Tsinghua University))<br>293T cells (human, gifts from Pan lab (Deng Pan, Tsinghua University))<br>SW480 cells (human, maintained in our laboratory)<br>Caco-2 cells (human, maintained in our laboratory)
Authentication	Cell lines were provided by trusted researchers.
Mycoplasma contamination	Cell lines were not tested for mycoplasma contamination but no indication of contamination was observed.
Commonly misidentified lines<br>(See ICLAC register)	No commonly misidentified cell lines were used.

Animals and other research organisms

Policy information about studies involving animals; ARRIVE guidelines recommended for reporting animal research, and Sex and Gender in Research

Laboratory animals	OT-I;Cas9, Rag1−/−, wild-type C57BL/6J, and NOG mice, both sexes, between the ages of 6-8 weeks were used for the study.
Wild animals	No wild animals were used in this study.
Reporting on sex	Both sexes were used in this study.
Field-collected samples	No field-collected samples were used in this study.
Ethics oversight	The mouse work was performed under the study protocol AAIS-Zengzx-01, as approved by the Institutional Animal Care and Use Committee (IACUC) of Peking University.

Note that full information on the approval of the study protocol must also be provided in the manuscript.

Plants

Seed stocks	*Report on the source of all seed stocks or other plant material used. If applicable, state the seed stock centre and catalogue number. If plant specimens were collected from the field, describe the collection location, date and sampling procedures.*
Novel plant genotypes	*Describe the methods by which all novel plant genotypes were produced. This includes those generated by transgenic approaches, gene editing, chemical/radiation-based mutagenesis and hybridization. For transgenic lines, describe the transformation method, the number of independent lines analyzed and the generation upon which experiments were performed. For gene-edited lines, describe the editor used, the endogenous sequence targeted for editing, the targeting guide RNA sequence (if applicable) and how the editor was applied.*
Authentication	*Describe any authentication procedures for each seed stock used or novel genotype generated. Describe any experiments used to assess the effect of a mutation and, where applicable, how potential secondary effects (e.g. second site T-DNA insertions, mosiacism, off-target gene editing) were examined.*

ChIP-seq

Data deposition

☒ Confirm that both raw and final processed data have been deposited in a public database such as GEO.

☐ Confirm that you have deposited or provided access to graph files (e.g. BED files) for the called peaks.

Data access links<br>*May remain private before publication.*	ChIP-seq data is deposited at GEO (GSE304035)<br>Token code: wbyjoeuuxfyhtcr
Files in database submission	*Provide a list of all files available in the database submission.*
Genome browser session<br>(e.g. UCSC)	*Provide a link to an anonymized genome browser session for "Initial submission" and "Revised version" documents only, to enable peer review. Write "no longer applicable" for "Final submission" documents.*

Methodology

Replicates	Each experimental condition consisted of three independent replicates, with a high degree of reproducibility observed among three replicates.
Sequencing depth	Raw double-end 50-bp sequences were filtered by quality (Q >30) and length (length > 20 bp) with Trim Galore. Total filtered sequences, which ranged between 32 and 55 million per sample, were aligned against the reference genome (mm10 release) with Bowtie2.
Antibodies	Anti-HA (Cell Signaling Technology, 3724)<br>Anti-IgG (Cell Signaling Technology, 2729)
Peak calling parameters	MACS2 was used for peak calling on uniquely aligned reads for each replicate with a p-value threshold of 0.05 (-p 0.05), genome size set to 2.65e9 (-g 2652783500), and duplicates handled automatically (--keep-dup auto). Pileup signal tracks were generated using the -B option. Peak annotation was performed with ChIPseeker, GenomicFeatures and org.Mm.eg.db packages.
Data quality	MACS2 was used for peak calling on uniquely aligned reads for each replicate with a p-value threshold of 0.05.
Software	Peak annotation was performed with ChIPseeker, GenomicFeatures and org.Mm.eg.db packages. Peaks heatmaps and differential peak analysis were performed with the DiffBind package. bigWig files from biological replicates were merged using bigWigMerge and bedGraphToBigWig.

Flow Cytometry

Plots

Confirm that:

☒ The axis labels state the marker and fluorochrome used (e.g. CD4-FITC).

☒ The axis scales are clearly visible. Include numbers along axes only for bottom left plot of group (a 'group' is an analysis of identical markers).

☒ All plots are contour plots with outliers or pseudocolor plots.

☒ A numerical value for number of cells or percentage (with statistics) is provided.

Methodology

Sample preparation	1.Primary OT-1;Cas9 CD8+ T cells were isolated from spleens and peripheral lymph nodes of OT-1;Cas9 mice.

Sample preparation	2.Subcutaneous tumor tissues were obtained after euthanasia of mice and dissociated into single-cell suspensions.
Instrument	BECKMAN COULTER CytoFLEX flow cytometry
Software	CytExpert software. Analysis of all flow cytometry data was done using FlowJo v10.8.1
Cell population abundance	not relevant
Gating strategy	In general, cells were first gated on FSC/SSC. Single cells were gated using FSC-H/FSC-A or SSC-H/SSC-A. Then, use DAPI or Zombie-NIR to distinguish live and dead cells. When detecting intracellular proteins, use Zombie-NIR. When detecting membrane proteins, use either DAPI or Zombie-NIR depending on the antibody staining panel.

☐ Tick this box to confirm that a figure exemplifying the gating strategy is provided in the Supplementary Information.
