# Supplementary material for: Arid3b suppresses CD8 + T cell infiltration and function in microsatellite-stable colorectal cancer via Runx3
Source: Nat Commun. 2026 May 15;17:6448. doi: 10.1038/s41467-026-73241-7 (PMC13376159; doi:10.1038/s41467-026-73241-7)
Supplement: Supplementary file 5 — Transparent Peer Review file [file 41467_2026_73241_MOESM5_ESM.pdf]

nature portfolio

Peer Review File

Arid3b suppresses CD8+ T cell infiltration and function in Microsatellite-stable colorectal cancer via Runx3

Corresponding Author: Professor Zhidong Gao

This file contains all reviewer reports in order by version, followed by all author rebuttals in order by version.

Version 0:

Reviewer comments:

Reviewer #1

(Remarks to the Author)
In this article, the authors conducted CRISPR screening in an animal model with the MSS colorectal cancer strain CMT93 and identified Arid3b in CD8+ T cells as a factor that negatively regulates the abundance of CD8+ T cells in tumors. Furthermore, the authors identified Runx3 as an effector factor through gene expression changes caused by genetic ablation of Arid3b and ARID3B ChIP-seq, demonstrating that ARID3B negatively regulates Runx3 in both transcription and protein - protein interactions, thereby suppressing the T-RM phenotype and weakening the antitumor immune response mediated by CD8+ T cells. The identification of the domains involved in Arid3b's transcriptional regulation of Runx3 and its protein-level interactions gives the impression of a relatively thorough investigation.
Major comments
・Although it is noted as a limitation, to discuss the importance of ARID3B in human anti-tumor immunity, it would be very good to examine the expression of ARID3B and RUNX3 in human tumor-infiltrating lymphocytes (TILs) using immunohistochemistry or publicly available or their own scRNA-seq data.
・Furthermore, what kind of therapeutic clinical applications do the authors consider? ARID3B-deficient CAR-T cell therapy seems like the most straightforward application. Therefore, the author should conduct in vivo experiments to address whether ARID3B-deficient CAR-T cells generated from human lymphocytes efficiently kill human cancer cells in NSG mice.
・The regulatory factors of ARID3B expression are also of interest. It would be worthwhile to consider which T cell fractions including naïve, central memory, effector memory, or exhausted T cells highly express ARID3B. The physiological role of ARID3B should be clarified. It would be better to conduct in-vivo experiments with mice of CD8(E8I)-Cre- Arid3b flox in order to validate the role of Arid3b in effector CD8+ T cells in the TME, LNs and spleens.
・It could be addressed whether this is limited to MSS colorectal cancer or can be applied to other cancer types.
・In lines 406-407, it is stated that targeting ARID3B is “safe and specific,” but for example, in Fig. 5C, the effect of Arid3b KO appears to persist even after Runx3 KO, so it is difficult to say that it is “highly specific and safe for Runx3.”
・Since the authors focused on the expression of ARID3B/3A, it would be good to consider chromatin remodeling. Although it is stated that it was direct binding to the promoter in this case, there were also protein-protein interactions. Therefore, control via chromatin remodeling is also plausible. Additionally, there is a high possibility that effects on other genes are mediated by chromatin remodeling. For example, could the authors perform ATAC-seq or ChIP-seq of histone modifications (H3K9me3) in Arid3b KO T cells?
・In Fig. 2, it is shown that Arid3b KO increases cytokine production, cytotoxic activity, and infiltration ability, but it is doubtful whether all of these effects can be explained by Runx3. If not, what is the responsible molecule for cytokine increase? Similarly, in Fig. 3, various changes in gene expression are described, but it is unclear from the flow of this paper whether these are related to Runx3 or not.

Minor comments
・I could not find Figure S2.
・Citations are needed for the two sentences in lines 285-286 and 288-290.

Reviewer #2

(Remarks to the Author)
This manuscript addresses a critical unmet need in colorectal cancer (CRC) immunotherapy: overcoming immune checkpoint inhibitor (ICI) resistance in microsatellite-stable/proficient mismatch repair (MSS/pMMR) CRC, which accounts for about 85% of cases and is characterized by a "cold" tumor microenvironment (TME) with sparse $CD8^+$ T cell infiltration. By combining in vivo CRISPR/Cas9 screening, functional validation, and multi-omics (RNA-seq/ChIP-seq) analyses, the authors identify Arid3b as a T cell-intrinsic suppressor of $CD8^+$ T cell infiltration and antitumor activity, and delineate a novel Arid3b-Runx3 axis that regulates T cell tissue residency and effector function. The study's core hypothesis is compelling, the experimental design is generally well-structured, and the findings hold significant translational potential for developing precision immunotherapies for MSS CRC.That said, several gaps in data validation, mechanistic depth, and translational context need to be addressed to strengthen the manuscript's rigor and impact. Below are specific, actionable revision recommendations:

1. Strengthen Validation of Runx3 Knockdown to Solidify the Core Mechanistic Axis
The manuscript's central claim—Arid3b exerts its effects via repressing Runx3—relies on "rescue" experiments with Runx3 knockdown (KD), but current data lack critical evidence for effective and specific Runx3 targeting. This is essential to rule out off-target effects and confirm causality.
Recommended Revisions:
Validate Runx3 KD efficiency at mRNA and protein levels: Add Western blot and RT-qPCR data showing reduced Runx3 expression in $CD8^+$ T cells transduced with sg-Runx3 (vs. sg-Int controls). Include at least two independent sgRNAs for Runx3 to confirm phenotype consistency (avoid reliance on a single sgRNA, which could introduce off-target artifacts).
Add a Runx3 KD monotherapy control: In the in vivo tumor experiments (Fig. 5E–G), include a "sg-Int + sg-Runx3" group to test whether Runx3 KD alone impairs $CD8^+$ T cell antitumor activity. This will distinguish between "rescue due to Runx3 loss" (supporting the Arid3b-Runx3 axis) and "non-specific toxicity of sg-Runx3."
Clarify T cell viability in double KO experiments: Add flow cytometry data (e.g., DAPI/Annexin V staining) to confirm that sg-Runx3 does not induce $CD8^+$ T cell death. This ensures that reduced $CD69^+CD103^+$ or IFN-γ^+ populations (Fig. 5C–D) reflect functional changes, not decreased cell survival.

2. Improve Reproducibility and Rigor of the In Vivo CRISPR Screen
The in vivo CRISPR screen (Fig. 1A–B) is the manuscript's foundational experiment, but current data lack replication and detailed controls to confirm Arid3b's status as a "key regulator."
Recommended Revisions:
Add screen replication: Conduct at least one independent technical replicate (e.g., new virus production, new T cell isolation/transduction) and one biological replicate (new cohort of $Rag1^{-/-}$ mice) to confirm that Arid3b sgRNAs are consistently enriched in tumor-infiltrating $CD8^+$ T cells. Present RRA scores or sgRNA fold-change values for replicates to demonstrate reproducibility.
Clarify library design and quality controls: Provide supplementary details on the sgRNA library, including: (1) how sgRNAs were selected (e.g., targeting exons of functional domains for Arid3b), (2) sgRNA representation in the "input" T cell population (to rule out library bias), and (3) the number/sequence of non-targeting control sgRNAs (beyond sg-Int) used to normalize enrichment scores.
Validate additional screen candidates: To contextualize Arid3b's importance, select 1–2 other top-enriched genes from the screen (e.g., genes with RRA scores comparable to Arid3b) and perform preliminary validation (e.g., sgRNA-mediated KO in $CD8^+$ T cells, tumor growth assays). This will show that Arid3b is not an isolated artifact but part of a broader network of T cell regulators.

3. Deepen Transcriptomic and Epigenomic Evidence for Arid3b-Runx3 Regulation
The RNA-seq and ChIP-seq experiments (Figs. 3–4) link Arid3b to Runx3 transcription but lack controls to confirm direct regulation and downstream signaling.
Recommended Revisions:
Add "Arid3b KO + Runx3 KD" RNA-seq: Include this group to confirm that transcriptional changes in Arid3b KO T cells (e.g., upregulated Cxcr3, Il2, Cd103; Fig. 3B) are dependent on Runx3. Present a heatmap or GSEA plot comparing this group to "sg-Int," "Arid3b KO," and "Runx3 KD" groups—this will directly demonstrate that Runx3 mediates Arid3b's effects on T cell activation/effector programs.
Strengthen ChIP-seq specificity: For the Runx3 promoter ChIP-seq data (Fig. 4A, G): (1) Show IgG control signals for the Runx3 locus to confirm that Arid3b binding is stronger than non-specific background; (2) Perform ChIP-qPCR for 1–2 negative control loci (e.g., a gene not regulated by Arid3b, such as a housekeeping gene) to rule out non-specific antibody binding.
Validate the ARID3B binding motif in primary T cells: The authors identify a TTTTATT motif in the Runx3 promoter (Fig. 4L) but only test it in 293T cells. Perform a CRISPR-mediated deletion of this motif in the endogenous Runx3 promoter of primary $CD8^+$ T cells, then assess Runx3 expression and Arid3b binding (via ChIP-qPCR). This will confirm that the motif is functional in a physiological T cell context.

4. Enhance Translational Relevance to Human MSS CRC
The manuscript focuses exclusively on murine models, which limits its relevance to human disease. Adding human data will significantly 提升 the study's clinical impact.
Recommended Revisions:
Analyze ARID3B/Runx3 expression in human MSS CRC samples: Use public datasets (e.g., TCGA-COAD, GEO) or primary patient samples to show: (1) ARID3B expression is higher in MSS CRC vs. MSI-H CRC; (2) Runx3 expression is lower in MSS CRC vs. MSI-H CRC; (3) ARID3B and Runx3 expression are negatively correlated in MSS CRC. For primary samples, add IHC staining of tumor sections to show ARID3B/Runx3 expression in $CD8^+$ T cells (via multiplex IHC, co-

staining for CD8, ARID3B, and Runx3).
Test ARID3B KD in primary human CD8+ T cells: Isolate CD8+ T cells from healthy donors or MSS CRC patients, transduce them with ARID3B sgRNAs, and assess: (1) Runx3 expression, (2) effector functon(IFN-γ/GZMB production), and (3) migration (transwell assays using human MSS CRC cell line-conditioned medium). If possible, perform a xenograft experiment using human MSS CRC cells and ARID3B KD human CD8+ T cells to confirm in vivo activity.

5. Clarify Minor Technical and Presentation Issues
Standardize flow cytometry gating: Provide supplementary gating strategies for all flow experiments (e.g., Fig. 1D, F, H; Fig. 2A, C, H), including gating for live cells (e.g., DAPI−), CD45+CD8+ T cells, and transfected cells (e.g., GFP+). This ensures data reproducibility.
Quantify Western blot signals: For all Western blots (e.g., Fig. 4C, E; Fig. S1A), add densitometry values normalized to a loading control (e.g., β-actin, H3) to quantify protein expression changes.
Discuss limitations explicitly: In the Discussion, acknowledge limitations such as (1) reliance on subcutaneous tumor models (vs. orthotopic models that better recapitulate CRC TME), (2) use of OVA-specific T cells (vs. polyclonal T cells that reflect physiological antitumor immunity), and (3) lack of combination therapy data (e.g., ARID3B KD + anti-PD-1, which would test translational potential for ICI sensitization).

In general, current study identifies a novel Arid3b-Runx3 axis that regulates CD8+ T cell function in MSS CRC, addressing a critical gap in understanding ICI resistance. With revisions to strengthen Runx3 KD validation, screen reproducibility, mechanistic depth, and human translational data, the manuscript will be a valuable contribution to CRC immunotherapy research. We strongly support revising and resubmitting this work.

Reviewer #3

(Remarks to the Author)
Wang, Hou, Luo and colleagues investigate the role of Arid3b and its downstream signaling in tumor-infiltrating CD8+ T cells in syngeneic, subcutaneous CRC tumor models in mice. The authors start off with an in vivo CRISPR/Cas9 screen, exploring possible negative regulators of CD8+ T cell infiltration in their primarily used mouse model (transplantation of OT-1 CD8+ Ova-specific T cells in Rag1 -/- mice with s.c. transplanted Ova-expressing CMT93 cell line). The screen identifies Arid3b -/- OT-1 CD8+ T cells to possess superior tumor-infiltrating capacity and anti-tumor efficacy in comparison to Arid3b-proficient OT-1 CD8+ T cells. Runx3 is suggested as a direct downstream-target of ARID3B, which is found to act as a transcriptional repressor of Runx3, thereby impairing its ability to activate the T cells' anti-tumor potential. The interaction of both proteins is validated in various assays, including ChIP-Seq analysis and IP-MS.
The authors describe an up to this point unknown interaction of the proteins ARID3B and RUNX3. In the described scenario, ARID3B acts as a repressor of Runx3 expression, thereby limiting OT-1 CD8+ T cell activation and tumor-infiltration. Knockout of Arid3b seems to substantially improve the infiltrative capacity and anti-tumoral activity of OT-1 CD8+ T cells against Ova-expressing CMT93 s.c. tumors, suggesting Arid3b as a possible target in cancer immunotherapy.
While the study identifies a potentially interesting and novel link between Arid3b and CD8+ T cell activation, the study has various technical limitations
.
Major limitations:
1. The mouse models used in this study are intended to mimic T cell infiltration in MSS colorectal cancer, as also indicated in the manuscript title. However, an Ova-expressing s.c. CMT93 tumor in Rag1-/- mice with only adoptively transferred, pre-activated OT-1 CD8+ T cells is an artificial, highly immunogenic, monoclonal system and does not at all recapitulate the immunobiology of MSS CRC. Ova is a strong, non-physiological antigen, leading to immediate and robust OT-1 T cell activation, in complete contrast to MSS CRC, which is characterized by low neoantigen expression and correspondingly low immunogenicity. We acknowledge that the identification of Arid3b as a negative regulator of T cell infiltration in solid tumors is of potential therapeutic interest. However, more advanced mouse models are necessary to show a potential benefit of Arid3b downregulation or knockout in T cells to combat MSS CRC specifically. Currently, the study provides no evidence linking Arid3b function in T cells to meaningful anti-tumor activity in MSS CRC.
2. Further, the authors conduct transcriptomic profiling of unstimulated and Ova-stimulated Arid3b-/- and Arid3b-proficient OT-1 CD8+ T cells. Without tumor co-culture, only very little transcriptional changes are observed between wild-type and knockout T cells, whereas various differentially expressed genes come up, when T cells are co-cultured with Ova-expressing target cells. We acknowledge that the further investigated target of ARID3B, Runx3, is significantly upregulated in both stimulated and unstimulated Arid3b-/- OT-1 CD8+ T cells. Nonetheless, given the above-mentioned limitation that the employed highly artificial Ova-expressing tumor model directly activates OT-1 T cells through strong TCR engagement, which contrasts the reality of MSS CRC, this experiment raises the concern that Arid3b downregulation or knockout will not increase T cell infiltration sufficiently, in more physiological models of MSS CRC, which provide almost no immunogenicity and T cell activation.
3. The authors are employing Arid3b-/- OT-1 CD8+ T cells, using two different gRNAs for targeting the Arid3b locus and state that "Western blot analysis confirmed efficient depletion of ARID3B protein" [line 128/129]. The respective blot (Sup. Fig.1A) does not confirm an efficient knock-out, as ARID3B bands are still clearly visible. At what time point after knockout was the Western blot performed? Please provide additional qPCR data for Arid3b mRNA expression and provide Western blot and qPCR confirmation of Arid3b-overexpressing cells, employed in downstream experiments.
4. The authors initially state that "Neither knockout nor overexpression of Arid3b altered CD69 expression in CD8+ T cells" [line 149/150], as apparently observed via flow cytometry (respective Figure Sup. Fig. 2 is missing). However, this conflicts their later results from RNA seq and subsequent flow cytometry analysis, showing CD69 upregulation upon Arid3b knockout. How do the authors explain these discrepancies?
5. Although not explicitly stated, it appears from the context that the MAGeCK analysis of the CRISPR/Cas9 screen (Fig 1B)

highlights genes broadly associated with enhanced OT-1 $CD8^+$ T cell tumor infiltration, not only candidate genes that are possible negative regulators of T cell infiltration. The authors should provide a brief discussion of additional candidate genes identified in their dataset beyond Arid3b. For improved clarity, a visualization that clearly distinguishes positive from negative regulators of T cell infiltration would be of interest. Moreover, several of the reported genes (e.g Ptprc (encoding CD45), Cd3d, Cd28) are well-established positive regulators of $CD8^+$ T cell responses and anti-tumor activity. Their presence in the list therefore adds little to addressing the central question of this assay.
6. The authors repeatedly suggest that Arid3b represents a promising target for precision immunotherapy. However, the manuscript does not provide any actual strategies or ideas for how this could be translated into clinical application. For instance, one could envision approaches such as engineering CAR T cells with Arid3b knockout to enhance their function. However, no such possibilities are discussed.
7. Unfortunately, Sup. Fig.2 is not provided in the article file, thus, important data is missing from the submitted manuscript.
8. According to the methods section, OT-1 CD8+ T cells are isolated from OT-1 mouse spleens and lymph nodes, then activated ex vivo using anti-CD3, anti-CD28 and IL2, before Arid3b knockout und afterwards further cultivated in presence of IL2, until transplantation into Rag1-/- mice. Pre-activation of T cells is an important detail and needs to be mentioned in the main text, as well as included in the schematic overview Fig. 1A.

Reviewer #4

(Remarks to the Author)
I co-reviewed this manuscript with one of the reviewers who provided the listed reports. This is part of the Nature Communications initiative to facilitate training in peer review and to provide appropriate recognition for Early Career Researchers who co-review manuscripts.

Version 1:

Reviewer comments:

Reviewer #1

(Remarks to the Author)
The authors adequately addressed the issues that I raised.

Reviewer #2

(Remarks to the Author)
I have no more concern

Reviewer #3

(Remarks to the Author)
We appreciate the authors’ efforts in revising the manuscript in response to our comments. In particular, the inclusion of human data significantly strengthened the central message of the study and the manuscript has significantly improved.
We only have a few minor details that should be refined to further enhance the clarity and overall quality of the data presentation:
1. The image quality of some subfigures should be substantially improved to allow the reader to clearly see details and read annotations (e.g. Fig. 3B). The resolution of some flow cytometry plots is also rather low, particularly in Figures S5 and S7. The gate annotations and displayed percentages should be clearly legible.
2. Fig S7: The axes of flow cytometry plots should be labeled not only with the fluorescent channels but also with the respective markers detected in each channel.
3. Several important details are missing from the methods section. For example, please provide comprehensive information on the plasmids and vectors used for the CRISPR/Cas9 screen (including cloning backbone, pseudotyping strategy, and other relevant details), rather than simply referring to “standard” protocols without citing an appropriate reference. Additionally, a clear description of the HA- and Flag-tagging of ARID3B and RUNX3 proteins should be included. The methods section should be revised and completed accordingly.

Reviewer #4

(Remarks to the Author)
I co-reviewed this manuscript with one of the reviewers who provided the listed reports. This is part of the Nature Communications initiative to facilitate training in peer review and to provide appropriate recognition for Early Career Researchers who co-review manuscripts.

Open Access This Peer Review File is licensed under a Creative Commons Attribution 4.0 International License, which permits use, sharing, adaptation, distribution and reproduction in any medium or format, as long as you give appropriate credit to the original author(s) and the source, provide a link to the Creative Commons license, and indicate if changes were made.
In cases where reviewers are anonymous, credit should be given to 'Anonymous Referee' and the source.
The images or other third party material in this Peer Review File are included in the article's Creative Commons license, unless indicated otherwise in a credit line to the material. If material is not included in the article's Creative Commons license and your intended use is not permitted by statutory regulation or exceeds the permitted use, you will need to obtain permission directly from the copyright holder.
To view a copy of this license, visit https://creativecommons.org/licenses/by/4.0/

Reviewer Comments & Author Responses:

Reviewer #1:

In this article, the authors conducted CRISPR screening in an animal model with the MSS colorectal cancer strain CMT93 and identified Arid3b in CD8+ T cells as a factor that negatively regulates the abundance of CD8+ T cells in tumors. Furthermore, the authors identified Runx3 as an effector factor through gene expression changes caused by genetic ablation of Arid3b and ARID3B ChIP-seq, demonstrating that ARID3B negatively regulates Runx3 in both transcription and protein - protein interactions, thereby suppressing the T-RM phenotype and weakening the antitumor immune response mediated by CD8+ T cells. The identification of the domains involved in Arid3b's transcriptional regulation of Runx3 and its protein-level interactions gives the impression of a relatively thorough investigation.

Major comments

Although it is noted as a limitation, to discuss the importance of ARID3B in human anti-tumor immunity, it would be very good to examine the expression of ARID3B and RUNX3 in human tumor-infiltrating lymphocytes (TILs) using immunohistochemistry or publicly available or their own scRNA-seq data.

We thank the Reviewer for this valuable suggestion regarding the importance of examining ARID3B and RUNX3 expression in human tumor-infiltrating lymphocytes (TILs). In response, we performed a detailed analysis using publicly available single-cell RNA sequencing (scRNA-seq) and spatial transcriptomic datasets. The key results are summarized below and have been integrated into the revised manuscript (new Figure 7J and Figure S6A-L, line 458- line 467).

(1) Analysis of public human scRNA-seq data[1] revealed a negative correlation between the expression levels of ARID3B and RUNX3 specifically in tumor-infiltrating $CD8^+$ T cells (new Figure S6A-J). This finding strongly aligns with our observations in murine models and supports the mechanism where ARID3B may regulate $CD8^+$ T cell function through the suppression of RUNX3.

(2) Subset analysis reveals heterogeneous expression of ARID3B. Further dissection of human $CD8^+$ T cell subsets showed that ARID3B expression is highly heterogeneous[2]. It is relatively low in naïve and effector subsets but is notably upregulated in certain functionally specialized populations. Most notably, tissue-resident memory T (Trm) cells exhibited relatively high levels of ARID3B expression (new Figure S6K). This suggests a potential role for ARID3B in T cell differentiation or functional specialization, providing a new direction for future investigation.

(3) Analysis of MSS colorectal cancer samples via spatial transcriptomics[3] indicated a low proportion of $CD8^+$ T cells in the tumor microenvironment, consistent with an immunologically "cold" tumor phenotype. Critically, within these sparse $CD8^+$ T cells, the expression of ARID3B and RUNX3 again displayed a negative correlation (new Figure 7J, new Figure S6L). This provides the first in-situ evidence of their relationship in human tumor tissue and places it within the clinically relevant context of an immunosuppressive niche.

In summary, by leveraging public omics data, we have successfully validated the negative correlation between ARID3B and RUNX3 in human tumor-infiltrating $CD8^+$ T cells and gained initial insights into its cell-subset specificity and spatial context. We believe this new analysis strengthens the clinical relevance of our study and directly addresses the reviewer's insightful point regarding the importance of ARID3B in human anti-tumor immunity.

Furthermore, what kind of therapeutic clinical applications do the authors consider? ARID3B-deficient CAR-T cell therapy seems like the most straightforward application. Therefore, the author should conduct in vivo experiments to address whether ARID3B-deficient CAR-T cells generated from human lymphocytes efficiently kill human cancer cells in NSG mice.

We thank the Reviewer for this valuable suggestion. We fully agree that developing *ARID3B*-deficient CAR-T cells represents a highly promising translational strategy. In support of this direction, we have obtained preliminary evidence regarding the function of ARID3B in human $CD8^+$ T cells. Our data demonstrate that *ARID3B* knockout in human $CD8^+$ T cells leads to upregulation of RUNX3, enhances the production of GZMB and IFN-γ, and significantly improves both cytotoxic activity against tumor cells and migratory capacity *in vitro*. Furthermore, in a humanized mouse model, *ARID3B*-deficient $CD8^+$ T cells showed superior tumor control and increased tumor infiltration (new Figure 7). Spatial transcriptomic analysis of MSS colorectal cancer samples also revealed a negative correlation between ARID3B and RUNX3 expression in tumor-infiltrating $CD8^+$ T cells, further supporting the relevance of this axis in human tumors.

As elaborated in the Discussion section (line 526-line 539), we propose that targeted disruption of *ARID3B* during CAR-T manufacturing could enhance T-cell infiltration and sustain antitumor function in immunologically cold tumors such as MSS CRC. Furthermore, this approach may synergize with immune checkpoint inhibitors to overcome the immunosuppressive barriers characteristic of this malignancy. The human $CD8^+$ T cell data provide a solid rationale for pursuing *ARID3B* targeting in CAR-T engineering. Future studies will focus on optimizing *ARID3B* disruption in human T cells and evaluating the safety and efficacy of *ARID3B*-deficient CAR-T cells in advanced preclinical models.

The regulatory factors of ARID3B expression are also of interest. It would be worthwhile to consider which T cell fractions including naïve, central memory, effector memory, or exhausted T cells highly express ARID3B. The physiological role of ARID3B should be clarified. It would be better to conduct in-vivo experiments with mice of CD8(E8I)-Cre-Arid3b flox in order to validate the role of Arid3b in effector CD8+ T cells in the TME, LNs and spleens.

We sincerely thank the Reviewer for their insightful and constructive comments. The points raised regarding the expression pattern of ARID3B in different T cell subsets and the necessity for *in vivo* functional validation are crucial for deepening this study. We fully agree with these suggestions and have conducted preliminary analyses based on available data, while also explicitly identifying these key directions as priorities for future research.

1. Regarding the expression pattern of ARID3B in different T cell subsets:

We analyzed public single-cell RNA sequencing data from CRC. Preliminary analysis indicates that the expression of ARID3B in human $CD8^+$ T cells is not uniform[2]. Its expression level is relatively low in naïve and effector T cells but appears upregulated in certain specific functional T cell subsets. Notably, we observed relatively higher ARID3B expression in the tissue-resident memory T cell subset (T_{rm}) (new Figure S6L). This observation aligns with our findings in the study that ARID3B inhibits effector function and may promote a dysfunctional state. We have added this preliminary observation to the Results section of the manuscript (line 458-line 467).

2. Regarding *in vivo* validation using CD8(E8I)-Cre-Arid3b flox mice:

We agree with the Reviewer's point that conditional knockout of Arid3b specifically in $CD8^+$ T cells represents the most direct and powerful evidence for validating its physiological function within the tumor microenvironment (TME), lymph nodes (LNs), and spleen. However, due to the lengthy timeframe typically required for constructing and validating such a conditional gene knockout mouse line, along with the complex breeding strategies involved, we were unable to complete this experiment within the revision period for the current study. To provide evidence of *in vivo* function under the available conditions, we employed adoptive T cell transfer model in wild-type C57BL/6, *Rag1*$^{-/-}$, and NOG mice (new Figure 1C-H, new Figure S1B-D, new Figure 7H-I). The results showed that compared to mice receiving control T cells, those receiving Arid3b-KO $CD8^+$ T cells exhibited significantly slowed tumor growth, along with enhanced infiltration and function of these T cells within the TME. This provides indirect yet compelling support for the conclusion that loss of Arid3b in $CD8^+$ T cells enhances their anti-tumor efficacy. We have identified the construction and utilization of the CD8(E8I)-Cre-Arid3b flox mouse model as a primary task for future research. This model will allow us to precisely dissect the spatiotemporal-specific functions of Arid3b in $CD8^+$ T cell fate determination across multiple contexts, including physiological development, homeostasis maintenance, immune responses, and tumor immunity, within the context of an intact immune system. We have emphasized this crucial future direction in the Discussion section of the manuscript (line 562-line 567).

It could be addressed whether this is limited to MSS colorectal cancer or can be applied to other cancer types.

We thank the Reviewer for this insightful comment. We agree that exploring the broader applicability of our findings beyond MSS colorectal cancer is of significant translational importance. In fact, we have conducted both *in vitro* coculture killing assays and *in vivo* experiments to evaluate the effect of Arid3b-deficient $CD8^+$ T cells across different microsatellite statuses and even different cancer types. Beyond the MSS CMT93-OVA model, we extended our investigation to include: (1) The MSI-H colorectal cancer cell line MC38-OVA: This model allows us to test whether the role of Arid3b is conserved across different microsatellite statuses of colorectal cancer. (2) The melanoma cell line B16F10-OVA: This model enables us to assess if the mechanism governed by Arid3b is generalizable to non-colorectal cancer types. The *in vitro* co-culture killing assay results demonstrated that Arid3b knockout significantly enhanced the cytotoxic capacity of $CD8^+$ T cells against MC38-OVA and B16F10-OVA cells (new Figure 2E, new Figure S2E). We established subcutaneous MC38-OVA and B16F10-OVA tumors in mice and intravenously transferred Arid3b-deficient or control $CD8^+$ T cells via tail vein injection. Consistent with the *in vitro* data and our findings in the CMT93-OVA model, Arid3b deficiency potently enhanced the anti-tumor efficacy of $CD8^+$ T cells, leading to markedly slowed tumor growth and a significantly increased proportion of $CD8^+$ T cell infiltration in both MC38-OVA and B16F10-OVA models (new Figure 1E-H, S1C-D). These data demonstrate that the enhanced anti-tumor function of Arid3b-deficient $CD8^+$ T cells is not limited to the MSS subtype of colorectal cancer. The effect is robustly observed both in an MSI-H colorectal cancer model and, importantly, in a melanoma model. This strongly suggests that the Arid3b-Runx3 axis represents a fundamental and broad regulatory mechanism of $CD8^+$ T cell function, potentially applicable to a wide range of cancer types.

In lines 406-407, it is stated that targeting ARID3B is "safe and specific," but for example, in Fig. 5C, the effect of Arid3b KO appears to persist even after Runx3 KO, so it is difficult to say that it is "highly specific and safe for Runx3."

We thank the Reviewer for this insightful comment. The Reviewer is correct that the data in Fig. 5C (now in new Figure 5E) suggest there may be additional, Runx3-independent mechanisms contributing to the full phenotype of Arid3b knockout (KO). We acknowledge this possibility and have tempered our language accordingly in the revised manuscript, changing "highly specific" to more accurately reflect our findings. However, we want to emphasize that our central argument regarding the superior specificity and safety profile of targeting ARID3B versus using a global demethylating agent remains robust and is based on a direct comparison of the two strategies: Decitabine, as a DNA methyltransferase inhibitor, causes genome-wide demethylation. While this can reactivate desirable genes like Runx3, it also leads to the uncontrolled expression of numerous other genes, including potentially deleterious ones (e.g., silenced oncogenes), which constitutes a significant risk for off-target toxicity. In contrast, targeting ARID3B is a transcriptional intervention. ARID3B acts as a direct transcriptional repressor binding to the Runx3 promoter. Inhibiting ARID3B, therefore, leads to the targeted de-repression of the Runx3 locus. While we do not rule out other ARID3B targets, which could explain the residual effect in the new Figure 5E, its action is inherently more focused than the epigenetic "sledgehammer" approach of decitabine. The genetic evidence of our study unequivocally places ARID3B upstream of Runx3 in a key pathway governing T-cell function.

Our claim of a "more targeted alternative" and "potential for greater safety and specificity" was made specifically in comparison to systemic demethylation therapies. Even if ARID3B regulates a small set of genes beyond Runx3, its targeting is still vastly more precise than the thousands of stochastic, genome-wide changes induced by decitabine. Therefore, from a therapeutic development perspective, a strategy aimed at inhibiting ARID3B holds the promise of a much wider therapeutic window than the use of a hypomethylating agent.

In summary, we agree that ARID3B may have functions beyond repressing Runx3. The core of our argument is that its targeted inhibition represents a mechanistically superior and inherently safer strategy to reactivate Runx3 and enhance T-cell function than inducing global DNA demethylation. We have revised the text to ensure this comparative context is unmistakable and to acknowledge the potential for additional ARID3B-regulated pathways (line 508-line 513).

Since the authors focused on the expression of ARID3B/3A, it would be good to consider chromatin remodeling. Although it is stated that it was direct binding to the promoter in this case, there were also protein-protein interactions. Therefore, control via chromatin remodeling is also plausible. Additionally, there is a high possibility that effects on other genes are mediated by chromatin remodeling. For example, could the authors perform ATAC-seq or ChIP-seq of histone modifications (H3K9me3) in Arid3b KO T cells?

We sincerely thank the Reviewer for raising this insightful and important point. We agree that investigating whether ARID3B functions through chromatin remodeling is crucial. In fact, we have already performed experiments along this line of inquiry. Specifically, we conducted ATAC-seq analysis on *Arid3b*-knockout and control $CD8^+$ T cells (new Figure S3C). Interestingly, the results revealed that loss of Arid3b did not induce broad or dramatic changes in genome-wide chromatin accessibility. This "negative" finding itself provides important information: it suggests that ARID3B

may not act as a global 'remodeler' of chromatin architecture, but rather may exert its transcriptional repressive function through more subtle mechanisms. For instance, it could recruit modifying enzymes such as KDM4C to alter local histone modification states (e.g., H3K9me3 and H3K27me3) without significantly altering DNA accessibility[4].

In Fig. 2, it is shown that Arid3b KO increases cytokine production, cytotoxic activity, and infiltration ability, but it is doubtful whether all of these effects can be explained by Runx3. If not, what is the responsible molecule for cytokine increase? Similarly, in Fig. 3, various changes in gene expression are described, but it is unclear from the flow of this paper whether these are related to Runx3 or not.

We sincerely thank the Reviewer for raising this critical point regarding the specificity of the Runx3-mediated mechanism.

To investigate whether Runx3 serves as a primary downstream mediator of the functional changes in Arid3b-deficient $CD8^+$ T cells, we generated and analyzed $CD8^+$ T cells with a double knockout of Arid3b and Runx3. These new data, now presented in new Figure 5, provide clear mechanistic insights:

(1) Runx3 is a major but not exclusive mediator: The experiments indicate that the key functional enhancements conferred by Arid3b deletion are largely dependent on Runx3. Specifically: The significant increase in IFN-γ and GZMB production induced by Arid3b knockout was largely abrogated in the double-knockout cells. The enhanced cytotoxic activity against tumor cells and the increased migratory capacity *in vitro* were also substantially reversed by the concomitant knockout of Runx3.

(2) Runx3 mediates the primary *in vivo* anti-tumor effect: In an adoptive T cell transfer model, the superior anti-tumor capacity exhibited by Arid3b-knockout $CD8^+$ T cells was significantly attenuated in the double-knockout cells. This suggests that Runx3 is a core component within the Arid3b regulatory network driving the *in vivo* functional advantage.

(3) Runx3 is required for the transcriptional reprogramming induced by Arid3b loss: To dissect the specific contribution of Runx3, we performed RNA-seq on control, Arid3b-KO, Runx3-KO, and Arid3b/Runx3 double-knockout $CD8^+$ T cells. A heatmap of DEGs between Arid3b-KO and double-knockout cells revealed a broad reversal of the transcriptional changes caused by Arid3b deletion upon additional Runx3 loss (new Figure 5J). Strikingly, pathway-focused analysis demonstrated that the differential expression of genes associated with key functional categories—including cytotoxicity, tissue trafficking, checkpoint/exhaustion, and activation/proliferation—which was prominent in Arid3b-KO cells, was consistently abolished in the double-knockout group (new Figure 5K). These findings definitively show that Runx3 is essential for initiating and sustaining the transcriptional rewiring driven by Arid3b deficiency. These comprehensive epistasis experiments demonstrate that Runx3 is the primary downstream effector responsible for mediating the key functional and transcriptomic impacts of Arid3b loss in $CD8^+$ T cells. This finding refines our mechanistic model, making it more precise and comprehensive: Arid3b primarily constrains the anti-tumor function of $CD8^+$ T cells by repressing the transcription factor Runx3, while Runx3-independent pathways may play auxiliary or contextual regulatory roles.

Minor comments

I could not find Figure S2.

We thank the Reviewer for pointing out this omission. Due to an oversight on our part, Figure S2 was accidentally not included in the initial submission. It has now been added to the supplementary information file. We apologize for any confusion this may have caused.

Citations are needed for the two sentences in lines 285-286 and 288-290.
We sincerely appreciate the reviewer's comment regarding the need for supporting citations. We have now addressed this by incorporating the suggested references at the indicated lines. We believe this addition strengthens the support for our claims and improves the manuscript's rigor.

Reviewer #2:
This manuscript addresses a critical unmet need in colorectal cancer (CRC) immunotherapy: overcoming immune checkpoint inhibitor (ICI) resistance in microsatellite-stable/proficient mismatch repair (MSS/pMMR) CRC, which accounts for about 85% of cases and is characterized by a "cold" tumor microenvironment (TME) with sparse $CD8^+$ T cell infiltration. By combining in vivo CRISPR/Cas9 screening, functional validation, and multi-omics (RNA-seq/ChIP-seq) analyses, the authors identify Arid3b as a T cell-intrinsic suppressor of $CD8^+$ T cell infiltration and antitumor activity, and delineate a novel Arid3b-Runx3 axis that regulates T cell tissue residency and effector function. The study's core hypothesis is compelling, the experimental design is generally well-structured, and the findings hold significant translational potential for developing precision immunotherapies for MSS CRC. That said, several gaps in data validation, mechanistic depth, and translational context need to be addressed to strengthen the manuscript's rigor and impact. Below are specific, actionable revision recommendations:

1. Strengthen Validation of Runx3 Knockdown to Solidify the Core Mechanistic Axis
The manuscript's central claim—Arid3b exerts its effects via repressing Runx3—relies on "rescue" experiments with Runx3 knockdown (KD), but current data lack critical evidence for effective and specific Runx3 targeting. This is essential to rule out off-target effects and confirm causality.

Recommended Revisions:
Validate Runx3 KD efficiency at mRNA and protein levels: Add Western blot and RT-qPCR data showing reduced Runx3 expression in $CD8^+$ T cells transduced with sg-Runx3 (vs. sg-Int controls). Include at least two independent sgRNAs for Runx3 to confirm phenotype consistency (avoid reliance on a single sgRNA, which could introduce off-target artifacts).
We thank the Reviewer for the valuable suggestion regarding the validation of Runx3 knockout efficiency. We have addressed this point in our revised manuscript. Specifically, we have included western blot data demonstrating a substantial reduction in RUNX3 protein expression in $CD8^+$ T cells transduced with sgRNAs targeting Runx3 compared to the sg-Int control (new Figure S5). Furthermore, in full accordance with the reviewer's important recommendation to avoid potential off-target artifacts, we employed two independent sgRNAs against Runx3. The western blot analysis and subsequent phenotypic assays (also detailed in new Figure S3) confirmed that both sgRNAs effectively reduced RUNX3 protein levels and produced consistent phenotypic outcomes, thereby strengthening the specificity and reliability of our conclusions.
Regarding the assessment of KD efficiency at the mRNA level via RT-qPCR, we would like to note a technical consideration specific to the CRISPR/sgRNA knockout approach. As the primary

mechanism often involves frameshift mutations or early stop codons leading to nonsense-mediated mRNA decay, residual mRNA transcripts may not accurately reflect functional gene knockout. Therefore, we believe that confirming the loss of the target protein via western blot provides a more direct and functionally relevant validation of knockout efficiency for our experimental system.

Add a Runx3 KD monotherapy control: In the in vivo tumor experiments (Fig. 5E–G), include a "sg-Int + sg-Runx3" group to test whether Runx3 KD alone impairs CD8⁺ T cell antitumor activity. This will distinguish between "rescue due to Runx3 loss" (supporting the Arid3b-Runx3 axis) and "non-specific toxicity of sg-Runx3."

We thank the Reviewer for this critical suggestion. We agree that inclusion of a Runx3 knockdown monotherapy control is essential for distinguishing between specific rescue effects and non-specific artifacts.

In response, we would like to respectfully point out that the requested "sg-Int + sg-Runx3" group is already included in our *in vivo* tumor experiments in the new Figure 5L–N. This group (labeled as "sg-Int + sg-Runx3," indicated by white triangles in the figure legend) was analyzed alongside the "sg-Int + sg-Int," "sg-Arid3b + sg-Int," and "sg-Arid3b + sg-Runx3" groups.

The data from this control group could address the Reviewer's concern. Specifically, we observed that Runx3 knockout alone ("sg-Int + sg-Runx3") significantly impaired the antitumor activity of $CD8^+$ T cells. This was evidenced by accelerated tumor growth (Figure 5L), increased tumor weight (Figure 5M), and a reduced proportion of $CD8^+$ T cell infiltration in subcutaneous tumor tissues (Figure 5N) compared to the "sg-Int + sg-Int" control group. Furthermore, we conducted additional *in vitro* experiments to validate the role of Runx3 in $CD8^+$ T cell function. Specifically, compared to sg-Int control cells, Runx3 knockout (sg-Runx3) significantly reduced CD69 expression (Figure 5D), tumor cell killing capacity (Figure 5G), and secretion of cytokines such as IFN-γ and GZMB (Figure 5E, F). These results confirm that the loss of Runx3 is detrimental to T cell function both *in vivo* and *in vitro*.

In the "sg-Arid3b + sg-Runx3" double-knockout group, despite the presence of the detrimental sg-Runx3, antitumor function was partially restored. This strongly supports our conclusion that the protumorigenic effect of Arid3b is mediated, at least in part, through its suppression of Runx3. The phenotype of the "sg-Int + sg-Runx3" group provides the necessary baseline for interpreting this rescue effect.

We have now further emphasized the interpretation of this key control group in the revised text on line 383-line 389.

Clarify T cell viability in double KO experiments: Add flow cytometry data (e.g., DAPI/Annexin V staining) to confirm that sg-Runx3 does not induce $CD8^+$ T cell death. This ensures that reduced $CD69^+CD103^+$ or IFN-γ^+ populations (Fig. 5C–D) reflect functional changes, not decreased cell survival.

We thank the Reviewer for raising this important point regarding T cell viability. We agree that confirming cell health is crucial to ensure that the observed functional deficits are not merely a consequence of cell death induced by Runx3 targeting. In response, we have performed Annexin V and DAPI staining on $CD8^+$ T cells transduced with the control sgRNA (sg-Int) or *Runx3*-targeting sgRNAs (sg-Runx3-1 or sg-Runx3-2). The new data, now included as new Figure S5H, clearly demonstrate that compared with the sg-Int control, the proportion of live cell populations (Annexin

V⁻DAPI⁻) in the *Runx3* knockout groups (sg-Runx3-1 or sg-Runx3-2) exhibited no significant difference. This confirms that the knockdown of Runx3 does not compromise T cell viability under our experimental conditions. Therefore, we could state that the reduction in $CD69^{+}CD103^{+}$, and IFN-γ^{+} populations is due to a genuine impairment of T cell function upon Runx3 loss, rather than decreased cell survival.

2. Improve Reproducibility and Rigor of the In Vivo CRISPR Screen

The in vivo CRISPR screen (Fig. 1A–B) is the manuscript's foundational experiment, but current data lack replication and detailed controls to confirm Arid3b's status as a "key regulator."

Recommended Revisions:

Add screen replication: Conduct at least one independent technical replicate (e.g., new virus production, new T cell isolation/transduction) and one biological replicate (new cohort of Rag1⁻/⁻ mice) to confirm that Arid3b sgRNAs are consistently enriched in tumor-infiltrating $CD8^{+}$ T cells. Present RRA scores or sgRNA fold-change values for replicates to demonstrate reproducibility.

We thank the Reviewer for the important suggestion to strengthen the reproducibility of our *in vivo* CRISPR screen. To address this point directly, we have performed an independent replicate of the screening experiment to validate the enrichment of Arid3b-targeting sgRNAs.

Specifically, we repeated the entire *in vivo* screening workflow using a different murine colorectal cancer cell line, MC38-OVA. All steps, including T cell isolation, library transduction, adoptive transfer, and sgRNA recovery/sequencing, were conducted independently. Integrated analysis of the original CMT93-OVA screen and the new MC38-OVA replicate demonstrates consistent and significant enrichment of Arid3b sgRNAs in tumor-infiltrating $CD8^{+}$ T cells (Figure 1). The reproducible enrichment observed across two independent tumor models provides strong evidence that Arid3b is a key negative regulator of $CD8^{+}$ T cell tumor infiltration, which directly addresses the reviewer's concern regarding replication and reproducibility.

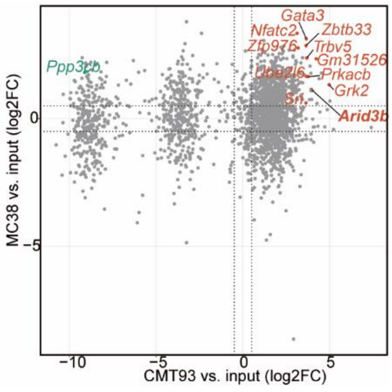

Fig 1. Integrated analysis of the CMT93-OVA screen and the MC38-OVA screen.

Clarify library design and quality controls: Provide supplementary details on the sgRNA library, including: (1) how sgRNAs were selected (e.g., targeting exons of functional domains for Arid3b), (2) sgRNA representation in the "input" T cell population (to rule out library bias), and (3) the number/sequence of non-targeting control sgRNAs (beyond sg-Int) used to normalize enrichment scores.

We thank the Reviewer for the valuable suggestions to clarify our library design and quality control. We have now provided supplementary details as requested:

(1) sgRNA selection: Our CRISPR sgRNA library was designed following an integrated, multi-step strategy to maximize biological relevance. First, genes with robust expression in $CD8^+$ T cells (genes expressed in >90% of $CD8^+$ T cells and ranking within the top 1500 by transcript abundance) were identified using single-cell RNA-seq data from The Human Protein Atlas. To include critical regulators, all transcription factors annotated in UniProt were added regardless of expression level. We further incorporated membrane-protein genes, immune-related functional genes, and genes differentially expressed upon $CD8^+$ T-cell activation. Essential and housekeeping genes were excluded to avoid survival bias. For each target gene, four high-efficiency sgRNAs were designed using the CRISPick algorithm. All sequences were validated by BLAT to ensure minimal off-target effects. Additional details have been included in the Methods section.

(2) sgRNA representation in "input" T cells: We directly assessed the evenness of sgRNA representation in the input T-cell population by calculating Gini index. A Gini index near 0 indicates perfect equality, while a value near 1 indicates maximal inequality. For the input samples, the Gini index were 0.044 in CMT93-OVA model and 0.045 in MC38-OVA model, confirming a highly uniform distribution of sgRNAs before selection. In contrast, tumor-infiltrating T-cell populations showed significantly higher Gini index (0.728 and 0.247, respectively), reflecting biologically meaningful enrichments and supporting the validity of the screen (Figure 2).

(3) Non-targeting control sgRNAs: In addition to the sg-Int control used in functional assays, the pooled library itself contains 500 non-targeting control sgRNAs (sg-Intergenic#1–500) targeting intergenic regions. These controls were used to normalize enrichment scores and define the background distribution in the CRISPR screening analysis. The full list of control sgRNA sequences is provided in Table S2.

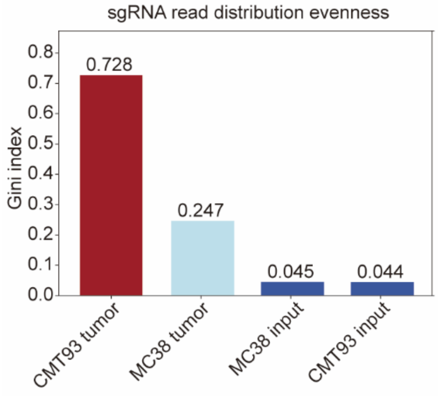

Fig. 2 Gini Index for the input and tumor groups in CMT93-OVA and MC38-OVA screen.

Validate additional screen candidates: To contextualize Arid3b's importance, select 1–2 other top-enriched genes from the screen (e.g., genes with RRA scores comparable to Arid3b) and perform preliminary validation (e.g., sgRNA-mediated KO in $CD8^+$ T cells, tumor growth assays). This will show that Arid3b is not an isolated artifact but part of a broader network of T cell regulators.

We thank the Reviewer for this constructive suggestion to contextualize the importance of Arid3b within the broader network of regulators identified in our screen. In response, we selected another top-enriched candidate, Znf131, for preliminary functional validation.

Our data show that genetic knockout of Znf131 in CD8+ T cells (with knockout efficiency confirmed by western blot) led to a significant increase in IFN-γ and GZMB production upon antigen-specific stimulation. Furthermore, Znf131-deficient CD8+ T cells exhibited markedly enhanced tumor-killing capacity against multiple OVA-expressing tumor cell lines (CMT93-OVA and MC38-OVA) in co-culture assays (new Figure S2 G-K).

These results demonstrate that: 1) the validity and robustness of our original screening approach by verifying another high-scoring hit. 2) Arid3b is not an isolated artifact but rather functions alongside other genes, such as Znf131, within a broader network of intrinsic negative regulators that constrain CD8+ T cell effector function.

3. Deepen Transcriptomic and Epigenomic Evidence for Arid3b-Runx3 Regulation

The RNA-seq and ChIP-seq experiments (Figs. 3–4) link Arid3b to Runx3 transcription but lack controls to confirm direct regulation and downstream signaling.

Recommended Revisions:

Add "Arid3b KO + Runx3 KD" RNA-seq: Include this group to confirm that transcriptional changes in Arid3b KO T cells (e.g., upregulated Cxcr3, Il2, Cd103; Fig. 3B) are dependent on Runx3. Present a heatmap or GSEA plot comparing this group to "sg-Int," "Arid3b KO," and "Runx3 KD" groups—this will directly demonstrate that Runx3 mediates Arid3b's effects on T cell activation/effector programs.

We thank the Reviewer for this excellent suggestion. In response, we have performed the requested RNA-seq experiment, which now includes the key *Arid3b*-KO + *Runx3*-KO double knockout group alongside the sg-Int (control), *Arid3b*-KO, and *Runx3*-KO groups. These analyses were conducted under both untreated conditions and after a 4-hour co-culture with CMT93-OVA tumor cells.

The new data, presented in new Figures 5J, K, provide direct evidence that *Runx3* mediates the transcriptional effects of *Arid3b* loss. Specifically:

(1) The heatmap of DEGs between the *Arid3b*-KO and double knockout groups (new Figure 5J) reveals a broad reversal of the transcriptional signature induced by *Arid3b* deletion upon concomitant *Runx3* knockout.

(2) Pathway-focused analysis (new Figure 5K) further demonstrates that the differential expression of genes associated with cytotoxicity, tissue trafficking, checkpoint exhaustion, and activation proliferation—which is prominent in *Arid3b*-KO cells, is consistently abolished in the double knockout group.

These findings demonstrate that the transcriptional rewiring and specific gene expression changes caused by *Arid3b* deficiency are critically dependent on *Runx3*.

Strengthen ChIP-seq specificity: For the Runx3 promoter ChIP-seq data (Fig. 4A, G): (1) Show IgG control signals for the Runx3 locus to confirm that Arid3b binding is stronger than non-specific background; (2) Perform ChIP-qPCR for 1–2 negative control loci (e.g., a gene not regulated by Arid3b, such as a housekeeping gene) to rule out non-specific antibody binding.

We thank the Reviewer for the critical suggestion to strengthen the specificity of our ChIP-seq data. We addressed both points raised by:

(1) IgG Control Signals: We want to point out that the IGV visualization of the ChIP-seq signals at

the Runx3 promoter locus in our original Figure 4A and 4G already included the IgG control track (as indicated in the figure legend). This was included precisely to demonstrate that the Arid3b binding signal is specific and significantly higher than the non-specific background. We apologize if this was not sufficiently clear in our initial submission and have now explicitly mentioned the presence of this control in the revised results section on line 266-271.

(2) ChIP-qPCR for Negative Control Loci: In direct response to the Reviewer's suggestion, we have performed additional ChIP-qPCR experiments using primers for the housekeeping gene Gapdh, which our RNA-seq data confirm is not regulated by Arid3b. The results, now included as Figure S4, show that Arid3b binding at this negative control loci is not significantly enriched compared to the IgG control. For completeness, we also show the IGV visualization of the ChIP-seq signals at the loci in the same supplement, confirming the absence of a peak.

Together, the pre-existing IgG control tracks and the new negative control ChIP-qPCR data provide robust and complementary evidence that the Arid3b binding at the Runx3 promoter is highly specific.

Validate the ARID3B binding motif in primary T cells: The authors identify a TTTTATT motif in the Runx3 promoter (Fig. 4L) but only test it in 293T cells. Perform a CRISPR-mediated deletion of this motif in the endogenous Runx3 promoter of primary CD8$^+$ T cells, then assess Runx3 expression and Arid3b binding (via ChIP-qPCR). This will confirm that the motif is functional in a physiological T cell context.

We thank the Reviewer for this critical suggestion to validate the functional role of the identified TTTTATT motif within its native chromatin context in primary T cells. We agree that such validation is essential for establishing direct mechanistic evidence.

In response, we have now performed the requested experiment using CRISPR/Cas9-mediated genomic editing. Specifically, we constructed a plasmid designed to express two sgRNAs that flank the TTTTATT motif in the endogenous Runx3 promoter, ensuring its complete deletion in primary CD8$^+$ T cells. Primary CD8$^+$ T cells were co-transduced with: (1) either a control sgRNA (sg-Int) or the motif-targeting sgRNAs (sg-Motif), and (2) a lentivirus expressing HA-tagged ARID3B to facilitate detection. Subsequently, we performed ChIP using an anti-HA antibody, followed by qPCR with primers amplifying the Runx3 promoter region encompassing the motif. The results, now included as new Figure 4N, provide direct physiological evidence that the motif is indispensable for ARID3B binding. In control cells (sg-Int + HA-ARID3B), we observed significant enrichment of the motif fragment. Importantly, this enrichment was completely abrogated in cells where the motif was deleted (sg-Motif + HA-ARID3B).

This experiment demonstrates that the TTTTATT sequence is necessary for ARID3B to occupy the Runx3 promoter in primary CD8$^+$ T cells. Together with our prior data showing that ARID3B represses Runx3 transcription, these new findings show the model that ARID3B directly binds to this specific motif to suppress Runx3 expression in a physiologically relevant setting.

4. Enhance Translational Relevance to Human MSS CRC

The manuscript focuses exclusively on murine models, which limits its relevance to human disease. Adding human data will significantly 提升 the study’s clinical impact.

Recommended Revisions:

Analyze ARID3B/Runx3 expression in human MSS CRC samples: Use public datasets (e.g.,

TCGA-COAD, GEO) or primary patient samples to show: (1) ARID3B expression is higher in MSS CRC vs. MSI-H CRC; (2) Runx3 expression is lower in MSS CRC vs. MSI-H CRC; (3) ARID3B and Runx3 expression are negatively correlated in MSS CRC. For primary samples, add IHC staining of tumor sections to show ARID3B/Runx3 expression in CD8$^+$ T cells (via multiplex IHC, co-staining for CD8, ARID3B, and Runx3).

We sincerely thank the Reviewer for raising this critical suggestion to validate the clinical relevance and the inverse relationship between ARID3B and Runx3 in human colorectal cancer (CRC). In direct response, we performed the following analyses:

(1) Analysis of TCGA-COAD bulk RNA-seq data: We analyzed ARID3B and RUNX3 expression in MSS versus MSI-H CRC samples from the TCGA-COAD cohort. However, our analysis revealed no significant differences in the expression levels of either ARID3B or RUNX3 between the two subtypes at the bulk tissue level (Figure 3A, B). Furthermore, a correlation analysis across MSS CRC samples did not show a significant negative correlation between ARID3B and RUNX3 expression (Figure 3C). We reason that this result is likely because bulk RNA-seq data represent an average signal from a heterogeneous mixture of cell types (including tumor cells, various stromal, and immune cells), which may obscure cell type-specific expression patterns, particularly in rare cell populations like tumor-infiltrating CD8$^+$ T cells.

(2) Spatial transcriptomics analysis in MSS CRC samples: To specifically interrogate the expression dynamics within the tumor microenvironment at a higher resolution, we performed spatial transcriptomics analysis on human MSS CRC tissue sections. This approach allowed us to resolve gene expression in situ. Our analysis confirmed that MSS CRC exhibits an immunologically "cold" phenotype, characterized by a relatively low abundance of CD8$^+$ T cell spots within the tumor microenvironment. Most importantly, within the spatially resolved CD8$^+$ T cell spots, we observed a significant negative correlation between ARID3B and RUNX3 expression levels (new Figure 7J). This cell type-specific analysis directly demonstrates the inverse relationship between ARID3B and its putative target Runx3 in the relevant immune cell population in human MSS CRC.

In summary, while bulk transcriptomic data from TCGA did not show the expected patterns, our high-resolution spatial transcriptomics analysis of MSS CRC tissues successfully revealed the predicted negative correlation between ARID3B and Runx3 specifically within CD8$^+$ T cells, thereby supporting the clinical relevance and specificity of our mechanistic model. We have included these new data and analyses as new Figure 7J and in the corresponding Results section.

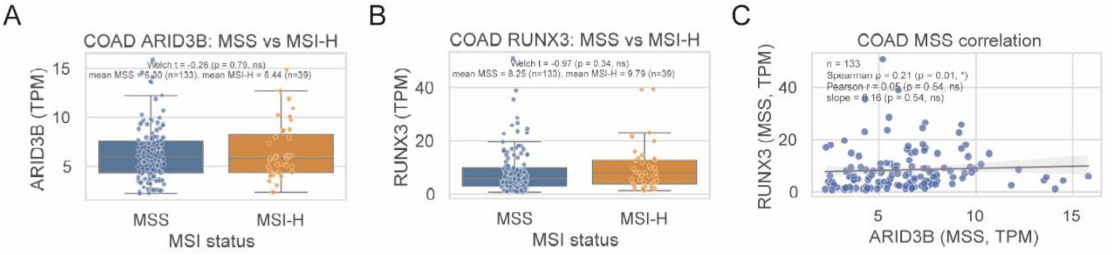

Fig. 3 Analysis of TCGA-COAD bulk RNA-seq data

(A, B) Expression of ARID3B (A) and RUNX3 (B) in MSS and MSI-H COAD.

(C) Correlation of expression between ARID3B and RUNX3 in MSS COAD.

Test ARID3B KD in primary human CD8$^+$ T cells: Isolate CD8$^+$ T cells from healthy donors or MSS CRC patients, transduce them with ARID3B sgRNAs, and assess: (1) Runx3

expression, (2) effector functon(IFN-γ/GZMB production), and (3) migration (transwell assays using human MSS CRC cell line-conditioned medium). If possible, perform a xenograft experiment using human MSS CRC cells and ARID3B KD human CD8⁺ T cells to confirm in vivo activity.

Thank you for this critical suggestion to validate our findings in human primary CD8⁺ T cells. We have now conducted a comprehensive series of experiments as recommended.

To investigate ARID3B's role in human cells, we isolated CD8⁺ T cells from healthy donor PBMCs and achieved efficient ARID3B knockout using CRISPR-Cas9, which was confirmed at the protein level by western blot. Consistent with our mouse data and your specific query, this knockout led to a clear increase in RUNX3 protein expression. Functionally, ARID3B-deficient human CD8⁺ T cells exhibited enhanced effector function, evidenced by elevated production of GZMB and IFN-γ upon stimulation. In co-culture assays with NY-ESO-1–expressing human MSS CRC cell lines (Caco-2 and SW480), these T cells showed markedly enhanced tumor-killing capacity. Furthermore, transwell migration assays confirmed that ARID3B knockout promoted the migratory capacity of human CD8⁺ T cells *in vitro* (Figure 7G). Most importantly, directly addressing your suggestion for *in vivo* validation, we performed a xenograft experiment. NOG mice bearing subcutaneous SW480 tumors (expressing NY-ESO-1) received an adoptive transfer of human PBMCs together with autologous either ARID3B-knockout or control CD8⁺ T cells. The results demonstrated that ARID3B deletion led to a significant reduction in tumor growth and a notable increase in CD8⁺ T cell tumor infiltration (new Figure 7). In summary, these results in human primary CD8⁺ T cells robustly recapitulate our key mechanistic and functional findings from mouse models: ARID3B acts as a negative regulator by repressing RUNX3, thereby constraining effector function, cytotoxicity, migration, and ultimately, anti-tumor efficacy *in vivo*.

5. Clarify Minor Technical and Presentation Issues

Standardize flow cytometry gating: Provide supplementary gating strategies for all flow experiments (e.g., Fig. 1D, F, H; Fig. 2A, C, H), including gating for live cells (e.g., DAPI⁻), CD45⁺CD8⁺ T cells, and transfected cells (e.g., GFP⁺). This ensures data reproducibility.

We thank the Reviewer for the suggestion to enhance methodological transparency. In response, we have now provided comprehensive supplementary gating strategies for all flow cytometry experiments reported in the manuscript. These strategies are included in the new Figure S7. This figure encompasses the gating logic applied to key datasets, including those presented in Figures 1D, F, H; 2A, C, H; and all other relevant flow cytometry panels.

Quantify Western blot signals: For all Western blots (e.g., Fig. 4C, E; Fig. S1A), add densitometry values normalized to a loading control (e.g., β-actin, H3) to quantify protein expression changes.

We thank the Reviewer for highlighting the importance of quantitative protein analysis. In response, we have now performed densitometric quantification for all Western blot data presented in the manuscript. The protein expression levels have been normalized to the indicated loading controls (β-actin or Histone H3). These quantitative data, presented below the respective blot images, are now included in Figure 4C, 4E, Figure S1A, and all other relevant figures.

Discuss limitations explicitly: In the Discussion, acknowledge limitations such as (1) reliance

on subcutaneous tumor models (vs. orthotopic models that better recapitulate CRC TME), (2) use of OVA-specific T cells (vs. polyclonal T cells that reflect physiological antitumor immunity), and (3) lack of combination therapy data (e.g., ARID3B KD + anti-PD-1, which would test translational potential for ICI sensitization).

We thank the Reviewer for highlighting these critical aspects for a balanced discussion. In response, we have explicitly acknowledged these limitations in the revised Discussion section (line 554-line 567). Specifically, we have incorporated the following points:

(1) The reliance on subcutaneous tumor models rather than orthotopic models that better recapitulate the native colorectal cancer tumor microenvironment.

(2) The use of OVA-specific T cells rather than polyclonal T cells to model physiological anti-tumor immunity.

(3) The absence of combination therapy data, such as testing ARID3B knockdown together with anti-PD-1 treatment to evaluate its potential for sensitizing tumors to immune checkpoint inhibition.

In general, current study identifies a novel Arid3b-Runx3 axis that regulates $CD8^+$ T cell function in MSS CRC, addressing a critical gap in understanding ICI resistance. With revisions to strengthen Runx3 KD validation, screen reproducibility, mechanistic depth, and human translational data, the manuscript will be a valuable contribution to CRC immunotherapy research. We strongly support revising and resubmitting this work.

Reviewer #3:

Wang, Hou, Luo and colleagues investigate the role of Arid3b and its downstream signaling in tumor-infiltrating CD8+ T cells in syngeneic, subcutaneous CRC tumor models in mice. The authors start off with an in vivo CRISPR/Cas9 screen, exploring possible negative regulators of CD8+ T cell infiltration in their primarily used mouse model (transplantation of OT-1 CD8+ Ova-specific T cells in Rag1 -/- mice with s.c. transplanted Ova-expressing CMT93 cell line). The screen identifies Arid3b -/- OT-1 CD8+ T cells to possess superior tumor-infiltrating capacity and anti-tumor efficacy in comparison to Arid3b-proficient OT-1 CD8+ T cells. Runx3 is suggested as a direct downstream-target of ARID3B, which is found to act as a transcriptional repressor of Runx3, thereby impairing its ability to activate the T cells' anti-tumor potential. The interaction of both proteins is validated in various assays, including ChIP-Seq analysis and IP-MS.

The authors describe an up to this point unknown interaction of the proteins ARID3B and RUNX3. In the described scenario, ARID3B acts as a repressor of Runx3 expression, thereby limiting OT-1 CD8+ T cell activation and tumor-infiltration. Knockout of Arid3b seems to substantially improve the infiltrative capacity and anti-tumoral activity of OT-1 CD8+ T cells against Ova-expressing CMT93 s.c. tumors, suggesting Arid3b as a possible target in cancer immunotherapy.

While the study identifies a potentially interesting and novel link between Arid3b and CD8+ T cell activation, the study has various technical limitations.

Major limitations:

1. The mouse models used in this study are intended to mimic T cell infiltration in MSS colorectal cancer, as also indicated in the manuscript title. However, an Ova-expressing s.c. CMT93 tumor in Rag1-/- mice with only adoptively transferred, pre-activated OT-1 CD8+ T cells is an artificial, highly immunogenic, monoclonal system and does not at all recapitulate

the immunobiology of MSS CRC. Ova is a strong, non-physiological antigen, leading to immediate and robust OT-1 T cell activation, in complete contrast to MSS CRC, which is characterized by low neoantigen expression and correspondingly low immunogenicity. We acknowledge that the identification of Arid3b as a negative regulator of T cell infiltration in solid tumors is of potential therapeutic interest. However, more advanced mouse models are necessary to show a potential benefit of Arid3b downregulation or knockout in T cells to combat MSS CRC specifically. Currently, the study provides no evidence linking Arid3b function in T cells to meaningful anti-tumor activity in MSS CRC.

We sincerely thank the Reviewer for raising this critical point regarding the translational relevance of our initial mouse models for MSS colorectal cancer. We agree that the OVA/OT-1 model represents a simplified and highly immunogenic system, which is a key limitation in directly modeling the immunosuppressive, low-antigenicity tumor microenvironment of human MSS CRC. Our primary rationale for employing this model was not to perfectly mimic the clinical TME, but to serve as a stringent and sensitive discovery platform to isolate the intrinsic role of Arid3b in regulating $CD8^{+}$ T cell function. In a model with weak endogenous immunity, the signal from a single genetic perturbation in T cells can be difficult to detect above background noise. The robust response in the OVA/OT-1 system provided the necessary dynamic range to clearly identify Arid3b as a potent negative regulator of T-cell infiltration and effector function, a discovery that would have been challenging in a more physiologically complex but less synchronized setting.

In response to this concern and to establish clear clinical relevance, we have performed a pivotal *in vivo* validation using a human tumor model. As detailed in the new Figure 7, we conducted a xenograft study wherein human ARID3B-knockout (vs. control) $CD8^{+}$ T cells were adoptively transferred into NOG mice bearing SW480 tumors, a well-characterized human MSS CRC cell line. This model, while lacking a full immune repertoire, allows for a direct assessment of the intrinsic anti-tumor capacity of human T cells in the context of a human MSS CRC microenvironment. The results are compelling: loss of ARID3B in human $CD8^{+}$ T cells led to a significant reduction in tumor growth and a concurrent increase in $CD8^{+}$ T cell tumor infiltration *in vivo*.

While more complex immunocompetent models of MSS CRC remain valuable for future mechanistic studies, this direct demonstration of enhanced anti-tumor efficacy in a model using human T cells against a human MSS CRC tumor provides strong and clinically relevant evidence that targeting the ARID3B pathway can potentiate T-cell-mediated immunity against low-immunogenicity CRC. We have also revised the Discussion to acknowledge the limitation of the initial model and to highlight the translational significance of these new *in vivo* human data.

2. Further, the authors conduct transcriptomic profiling of unstimulated and Ova-stimulated Arid3b-/- and Arid3b-proficient OT-1 CD8+ T cells. Without tumor co-culture, only very little transcriptional changes are observed between wild-type and knockout T cells, whereas various differentially expressed genes come up, when T cells are co-cultured with Ova-expressing target cells. We acknowledge that the further investigated target of ARID3B, Runx3, is significantly upregulated in both stimulated and unstimulated Arid3b-/- OT-1 CD8+ T cells. Nonetheless, given the above-mentioned limitation that the employed highly artificial Ova-expressing tumor model directly activates OT-1 T cells through strong TCR engagement, which contrasts the reality of MSS CRC, this experiment raises the concern that Arid3b

downregulation or knockout will not increase T cell infiltration sufficiently, in more physiological models of MSS CRC, which provide almost no immunogenicity and T cell activation.

We thank the Reviewer for this insightful comment, which allows us to further clarify the biological interpretation of our transcriptomic data and its relevance to MSS CRC. The Reviewer correctly notes that profound transcriptional changes between *Arid3b*-knockout and control $CD8^+$ T cells are primarily observed upon strong antigenic stimulation, rather than in the unstimulated state. This observation is, in fact, central to our mechanistic understanding: Arid3b functions not as a global repressor of basal T cell state, but as a potent activation-dependent negative regulator. Its role becomes critical precisely when T cells receive TCR signals and embark on effector differentiation. In the context of solid tumors like MSS CRC, the limiting factor is not the complete absence of T cell activation, but the suboptimal function and eventual exhaustion of the tumor-infiltrating lymphocytes that do recognize tumor antigens. Our data suggest that Arid3b acts as an internal brake on these activated cells. Therefore, its deletion could specifically enhance the function of the clinically relevant, tumor-antigen-engaged T cell pool, even within a low-immunogenicity microenvironment. Most importantly, we have now directly tested this hypothesis in a model that closely approximates human MSS CRC. As presented in the new Figure 7, ARID3B knockout in primary human $CD8^+$ T cells significantly enhanced their cytotoxic capacity against the MSS CRC cell line SW480 *in vitro*, and more critically, led to superior tumor control and increased intratumoral accumulation *in vivo*. This model uses no exogenous strong antigen and relies on the native, low-immunogenicity interaction between human T cells and a human MSS CRC tumor. Thus, while the OVA/OT-1 system provided a high-signal platform for discovery, the validation in a human MSS CRC model confirms that the Arid3b-mediated restraint pathway is operative and therapeutically targetable in the context of clinically relevant, low-immunogenicity tumors.

3. The authors are employing Arid3b-/- OT-1 CD8+ T cells, using two different gRNAs for targeting the Arid3b locus and state that "Western blot analysis confirmed efficient depletion of ARID3B protein" [line 128/129]. The respective blot (Sup. Fig.1A) does not confirm an efficient knock-out, as ARID3B bands are still clearly visible. At what time point after knockout was the Western blot performed? Please provide additional qPCR data for Arid3b mRNA expression and provide Western blot and qPCR confirmation of Arid3b-overexpressing cells, employed in downstream experiments.

We appreciate the Reviewer's careful assessment of the *Arid3b* knockout validation. In response, we have performed additional experiments and provided further clarification in the revised manuscript.

Since our approach uses CRISPR/Cas9 to create genomic edits, which can introduce frameshift or premature stop codons, the resulting transcripts may still be detectable by qPCR but often undergo nonsense-mediated decay or encode non-functional protein fragments. Therefore, we consider Western blot analysis the most direct and functionally relevant method to confirm effective protein depletion.

(1) Timing of Western Blot Analysis: The Western blot confirming *Arid3b* knockout is performed at day 7 post-transduction of the sgRNAs. The updated blot (new Figure S1A) clearly demonstrates efficient loss of ARID3B protein in $CD8^+$ T cells transduced with both sgRNAs targeting Arid3b, compared to the control.

(2) Validation of ARID3B-Overexpressing Cells:
As requested, we have also included Western blot confirmation of *Arid3b* overexpression in the gain-of-function experiments (new Figure 4E).

4. The authors initially state that "Neither knockout nor overexpression of Arid3b altered CD69 expression in CD8⁺ T cells" [line 149/150], as apparently observed via flow cytometry (respective Figure Sup. Fig. 2 is missing). However, this conflicts their later results from RNA seq and subsequent flow cytometry analysis, showing CD69 upregulation upon Arid3b knockout. How do the authors explain these discrepancies?
Thank you for raising this critical point regarding the apparent discrepancy between our RNA-seq and initial flow cytometry data for CD69 expression.
We agree that the lack of change in CD69 surface protein by flow cytometry, despite a significant upregulation in its mRNA from RNA-seq, requires a coherent explanation. We believe the most plausible explanation lies in the transient nature of CD69 expression combined with the specific timing of our assays.
CD69 is an early activation marker whose expression peaks rapidly within the first 4-24 hours post-T cell activation and subsequently declines[5]. Our experimental timeline provides crucial context: Day 0: Isolation of naïve T cells from the spleen. Day 1: Viral transduction for Arid3b knockout. Day 2-3: Flow sorting of transduced $CD8^+$ T cells. Day 7: Assessment of CD69 by flow cytometry. In this setup, the critical window for de novo CD69 induction would have been around the activation/transduction phase (Days 1-3). By Day 7, the cells were in a more sustained culture phase, and the transient wave of CD69 expression, even if it was initially enhanced by Arid3b knockout, would have likely subsided. Therefore, our flow cytometry analysis at Day 7 simply missed the temporal window to capture the potential increase in CD69 surface protein that was reflected at the transcriptional level earlier. This interpretation is consistent with the established biology of CD69 and resolves the apparent conflict. Taken together, our data demonstrate that Arid3b deficiency indeed significantly upregulates Cd69 at the transcriptional level. However, this upregulation failed to translate into a significant change in surface protein expression under our experimental conditions. We hope this explanation could addresses the Reviewer's concern and strengthens the interpretation of our data.

5. Although not explicitly stated, it appears from the context that the MAGeCK analysis of the CRISPR/Cas9 screen (Fig 1B) highlights genes broadly associated with enhanced OT-1 $CD8^+$ T cell tumor infiltration, not only candidate genes that are possible negative regulators of T cell infiltration. The authors should provide a brief discussion of additional candidate genes identified in their dataset beyond Arid3b. For improved clarity, a visualization that clearly distinguishes positive from negative regulators of T cell infiltration would be of interest. Moreover, several of the reported genes (e.g Ptprc (encoding CD45), Cd3d, Cd28) are well-established positive regulators of $CD8^+$ T cell responses and anti-tumor activity. Their presence in the list therefore adds little to addressing the central question of this assay.
We thank the Reviewer for this important observation and constructive suggestion. The Reviewer correctly notes that our loss-of-function screen inherently identifies both positive regulators (knockout of which impairs $CD8^+$ T cells infiltration, leading to depletion in tumors—representing negative selection) and negative regulators (knockout of which enhances $CD8^+$ T cells infiltration,

leading to enrichment in tumors—representing positive selection). The list in Figure 1B corresponds to the positive-selection outcomes. In direct response to the reviewer's comments, we have implemented the following revisions:

(1) We have separately visualized the enriched genes and performed corresponding pathway enrichment analyses from both the positive- and negative-selection perspectives (Figure 4A-D).

(2) In the Results section (line 184–line 193), we performed a preliminary *in vitro* functional validation of another highly enriched gene, Znf131, whose role in CD8$^+$ T cells has not been well characterized (Figure S2G-K). Furthermore, in the Discussion section, we have added a new paragraph briefly mentioning other notable candidate genes identified in the screen (e.g., Zfp131, Grk2) and highlighting the need for future studies to elucidate their specific functions (line 540–line 551).

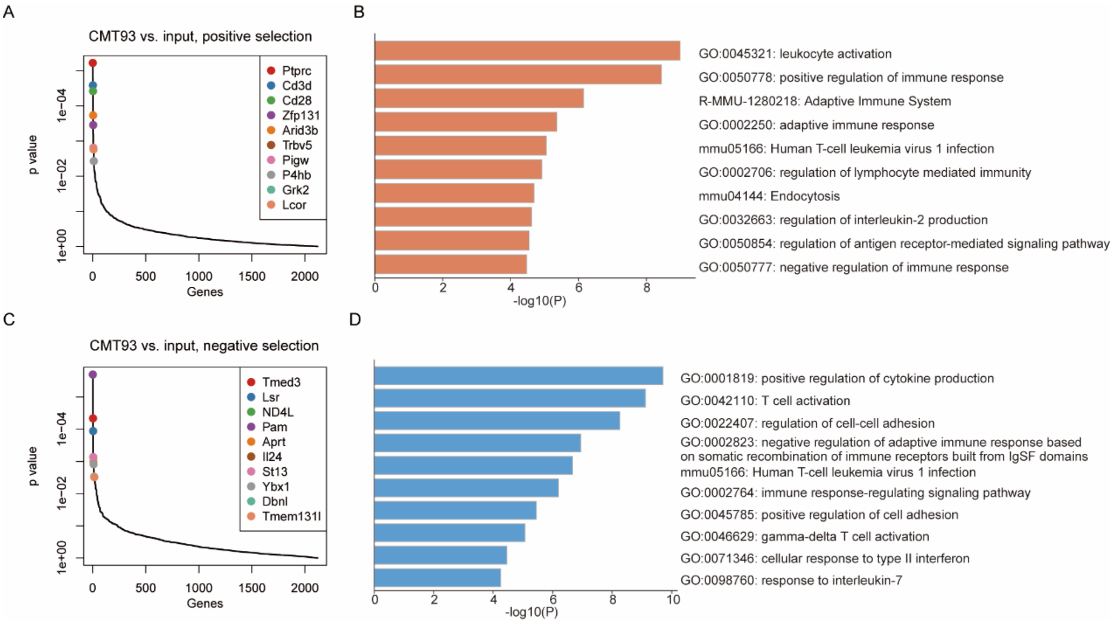

Fig. 4 Analysis of the CMT93-OVA library screening data.

(A) Top candidate genes ranked by p-value using MAGeCK analysis (positive selection).

(B) Pathway enrichment analysis of genes enriched in the positive selection.

(C) Top candidate genes ranked by p-value using MAGeCK analysis (negative selection).

(D) Pathway enrichment analysis of genes enriched in the negative selection.

6. The authors repeatedly suggest that Arid3b represents a promising target for precision immunotherapy. However, the manuscript does not provide any actual strategies or ideas for how this could be translated into clinical application. For instance, one could envision approaches such as engineering CAR T cells with Arid3b knockout to enhance their function. However, no such possibilities are discussed.

We thank the Reviewer for this insightful comment and for emphasizing the importance of discussing tangible translational strategies. We agree that moving beyond the conceptual suggestion to concrete applications is critical for the field. In response, we have now significantly expanded our Discussion section (line 526-line 539) to outline specific, actionable strategies for targeting ARID3B in precision immunotherapy. The newly added text discusses:

(1) Cell Therapy Engineering: Our newly obtained experimental data in human CD8$^+$ T cells provide a direct rationale for this approach. We found that ARID3B knockout in primary human CD8$^+$ T

cells leads to enhanced effector function (increased production of IFN-γ and GZMB), superior cytotoxic activity against tumor cells, and improved migratory capacity. These *in vitro* findings, supported by evidence of increased tumor infiltration and control in a humanized mouse model, strongly indicate that disabling ARID3B endows human T cells with favorable attributes for antitumor immunity. Therefore, we explicitly propose the generation of ARID3B-deficient CAR-T or TCR-engineered T cells. We discuss the potential of using CRISPR-Cas9 or shRNA delivered via lentiviral vectors to knock out or knock down ARID3B during the ex vivo manufacturing process of patient-specific T cell products. We hypothesize that this approach, grounded in our mechanistic and functional data, could universally enhance T-cell fitness, persistence, and effector function across a range of solid tumors.
(2) Pharmacological Inhibition: We discuss the potential for developing small-molecule inhibitors targeting the ARID DNA-binding domain of ARID3B. While no such compounds currently exist, we suggest that our findings provide a strong rationale for high-throughput screening campaigns to identify molecules that disrupt ARID3B's interaction with its target gene promoters, such as Runx3.
(3) Combination with Standard Care: We elaborate on how ARID3B-targeting strategies could be synergistically combined with existing immunotherapies, particularly immune checkpoint inhibitors (ICBs). We posit that by intrinsically reprogramming T cells to a more resilient and infiltrative state (e.g., via increased CXCR3 expression), ARID3B inhibition could help overcome the T-cell exhaustion and poor tumor infiltration that often limit the efficacy of ICBs in cancers like microsatellite-stable colorectal cancer.

7. Unfortunately, Sup. Fig.2 is not provided in the article file, thus, important data is missing from the submitted manuscript.
We thank the Reviewer for their careful reading of the manuscript and for noting the absence of Figure S2. This was an oversight in our initial submission, and we sincerely apologize for the error. The figure has now been properly included in the revised supplementary information file.

8. According to the methods section, OT-1 CD8+ T cells are isolated from OT-1 mouse spleens and lymph nodes, then activated ex vivo using anti-CD3, anti-CD28 and IL2, before Arid3b knockout und afterwards further cultivated in presence of IL2, until transplantation into Rag1-/- mice. Pre-activation of T cells is an important detail and needs to be mentioned in the main text, as well as included in the schematic overview Fig. 1A.
We thank the Reviewer for raising this important point. We agree that the pre-activation step of the $CD8^+$ T cells is a critical methodological detail that provides essential context for the experiments. In response, we have now explicitly described the pre-activation step of $CD8^+$ T cells in the Results section (lines 109-112) and correspondingly updated the schematic diagram in new Figure 1A. The revised text now states: "Concurrently, $CD8^+$ T cells were isolated from the spleens and lymph nodes of OT-1; Cas9 mice, pre-activated ex vivo with anti-CD3/CD28 antibodies, transduced with a pooled sgRNA library targeting immune regulatory genes, and supplemented with recombinant murine IL-2."

Reference

[1] LEE H O, HONG Y, ETLIOGLU H E, et al. Lineage-dependent gene expression

programs influence the immune landscape of colorectal cancer [J]. Nat Genet, 2020, 52(6): 594-603.

[2] ZHANG L, LI Z, SKRZYPCZYNSKA K M, et al. Single-Cell Analyses Inform Mechanisms of Myeloid-Targeted Therapies in Colon Cancer [J]. Cell, 2020, 181(2): 442-59.e29.

[3] REN P, ZHANG R, WANG Y, et al. Systematic benchmarking of high-throughput subcellular spatial transcriptomics platforms across human tumors [J]. Nat Commun, 2025, 16(1): 9232.

[4] LIAO T T, HSU W H, HO C H, et al. let-7 Modulates Chromatin Configuration and Target Gene Repression through Regulation of the ARID3B Complex [J]. Cell Rep, 2016, 14(3): 520-33.

[5] ANTAS P R, OLIVEIRA E B, MILAGRES A S, et al. Kinetics of T cell-activation molecules in response to Mycobacterium tuberculosis antigens [J]. Mem Inst Oswaldo Cruz, 2002, 97(8): 1097-9.

Reviewer Comments & Author Responses:

Reviewer #3:

We appreciate the authors' efforts in revising the manuscript in response to our comments. In particular, the inclusion of human data significantly strengthened the central message of the study and the manuscript has significantly improved.

We only have a few minor details that should be refined to further enhance the clarity and overall quality of the data presentation:

1. The image quality of some subfigures should be substantially improved to allow the reader to clearly see details and read annotations (e.g. Fig. 3B). The resolution of some flow cytometry plots is also rather low, particularly in Figures S5 and S7. The gate annotations and displayed percentages should be clearly legible.

We thank the reviewer for the valuable suggestions. We have enlarged the annotation font size in Figure 3B and improved the resolution of the flow cytometry plots in Figures S5 and S7. All gate annotations and percentage values are now clearly legible.

2. Fig S7: The axes of flow cytometry plots should be labeled not only with the fluorescent channels but also with the respective markers detected in each channel.

We apologize for this oversight. In the revised Supplementary Figure S7, we have now added the respective marker names alongside the fluorescent channel labels on both axes. This modification makes the figure self-explanatory and easier to interpret.

3. Several important details are missing from the methods section. For example, please provide comprehensive information on the plasmids and vectors used for the CRISPR/Cas9 screen (including cloning backbone, pseudotyping strategy, and other relevant details), rather than simply referring to "standard" protocols without citing an appropriate reference. Additionally, a clear description of the HA- and Flag-tagging of ARID3B and RUNX3 proteins should be included. The methods section should be revised and completed accordingly.

We agree with the reviewer that methodological transparency is critical. Accordingly, we have substantially expanded the Methods section to include the missing details:

(1) CRISPR/Cas9 screen: The plasmid used for CRISPR/Cas9 screening and for knockout of genes such as Arid3b, Runx3, and Znf131 in subsequent experiments is PMYs, which is a retroviral backbone[1]. The key elements are shown in Figure 1A. Virus production is described in the Methods (lines 649–655).

(2) HA and Flag tagging of ARID3B and RUNX3: The plasmids expressing HA-tagged ARID3B and Flag-tagged RUNX3 were also constructed using the PMYs vector. The HA or Flag tag is expressed at the N-terminus of ARID3B or RUNX3. To facilitate fluorescence-based selection, EGFP or tdTomato fluorescent protein is fused to the C-terminus via a P2A linker (lines 809-811).

We believe these additions fully address the reviewer's concerns and make the study reproducible.

We thank the reviewers again for their time and insightful comments, which have helped us to significantly improve the quality of our manuscript.

Reference

[1] KITAMURA T, KOSHINO Y, SHIBATA F, et al. Retrovirus-mediated gene transfer and

expression cloning: powerful tools in functional genomics [J]. Exp Hematol, 2003, 31(11): 1007-14.
